# Cancer-associated fibroblast-derived POSTN as a metabolic “switch” shapes lipid-stressed macrophage polarization in gastric cancer

**DOI:** 10.1016/j.redox.2026.104389

**Published:** 2026-09-09

**Authors:** Qin Ding, Ziteng Li, Ruiwen Liu, Wanjing Feng, Jiayi Wei, Chengye Liu, Masami Yamamoto, Tetsuya Tsukamoto, Sachiyo Nomura, Shenglin Huang, Xiaodong Zhu

**Affiliations:** a Department of Medical Oncology, Fudan University Shanghai Cancer Center, and Shanghai Key Laboratory of Medical Epigenetics, Institutes of Biomedical Sciences, Fudan University, Shanghai, China; b Department of Oncology, Shanghai Medical College, Fudan University, Shanghai, China; c Laboratory of Physiological Pathology, School of Veterinary Nursing and Technology, Nippon Veterinary and Life Science University, Tokyo, Japan; d Department of Diagnostic Pathology, Fujita Health University School of Medicine, Toyoake, Aichi, Japan; e Department of Gastrointestinal Surgery, Graduate School of Medicine, The University of Tokyo, Tokyo, Japan; f State Key Laboratory of Genetics and Development of Complex Phenotypes, Fudan University, Shanghai, China

**Keywords:** Gastric cancer, POSTN, Fatty acid metabolism, Lipid-stressed macrophage, Immunotherapy response

## Abstract

**Background:**

Cancer-associated fibroblasts (CAFs) foster an immunosuppressive tumor microenvironment (TME) and confer resistance to immune checkpoint blockade (ICB) in gastric cancer (GC). However, the mechanisms by which specific CAF subsets regulate metabolic crosstalk and immune evasion remain poorly defined.

**Methods:**

We integrated bulk and single-cell RNA-sequencing data, spatial transcriptomics, and clinical cohorts of GC patients treated with immunotherapy. Functional validation was performed using in vitro co-culture systems, multiple murine models, and lipid nanoparticle (LNP)-encapsulated siRNA targeting Postn.

**Results:**

We identified POSTN ^+^ CAFs as a key subset enriched in ICB non-responders and associated with poor prognosis. Mechanistically, POSTN secreted by CAFs engaged integrin β1 (ITGB1) on tumor cells and macrophages, activating the PI3K/AKT/mTOR signaling axis and upregulating PPARγ. This signaling cascade drove lipid metabolic reprogramming, characterized by increased lipid accumulation and oxidative stress, and promoted the polarization of macrophages toward an immunosuppressive, lipid-stressed M2 phenotype. Targeting POSTN signaling with LNP-formulated siPostn attenuated tumor growth, suppressed lipid metabolism, and reduced M2 macrophage infiltration in the TME.

**Conclusion:**

This work reveals that POSTN ^+^ CAFs establish an immunosuppressive TME and are associated with ICB non-response in GC by remodeling lipid metabolism through the ITGB1-PI3K/AKT/mTOR-PPARγ axis, a finding that underscores the therapeutic value of intervening in this specific CAF-macrophage metabolic crosstalk.

## Highlights

• POSTN ^+^ cancer-associated fibroblasts are enriched in gastric cancer patients who do not respond to immunotherapy and are associated with poor clinical outcomes.
• CAF-derived POSTN enhances tumor cell proliferation and establishes an immunosuppressive microenvironment by promoting M2-like lipid-stressed macrophage polarization.
• POSTN activates ITGB1–PI3K/AKT/mTOR–PPARγ signaling to reprogram lipid metabolism in both tumor cells and macrophages.
• Silencing POSTN with lipid nanoparticle-delivered siRNA reduces lipid metabolic activity, remodels the immune microenvironment, and suppresses tumor growth.
• Multi-omics, single-cell, and spatial transcriptomics were integrated from large clinical cohorts with in vitro and in vivo functional validation to establish mechanistic causality.

## Graphical abstract

**Figure:**
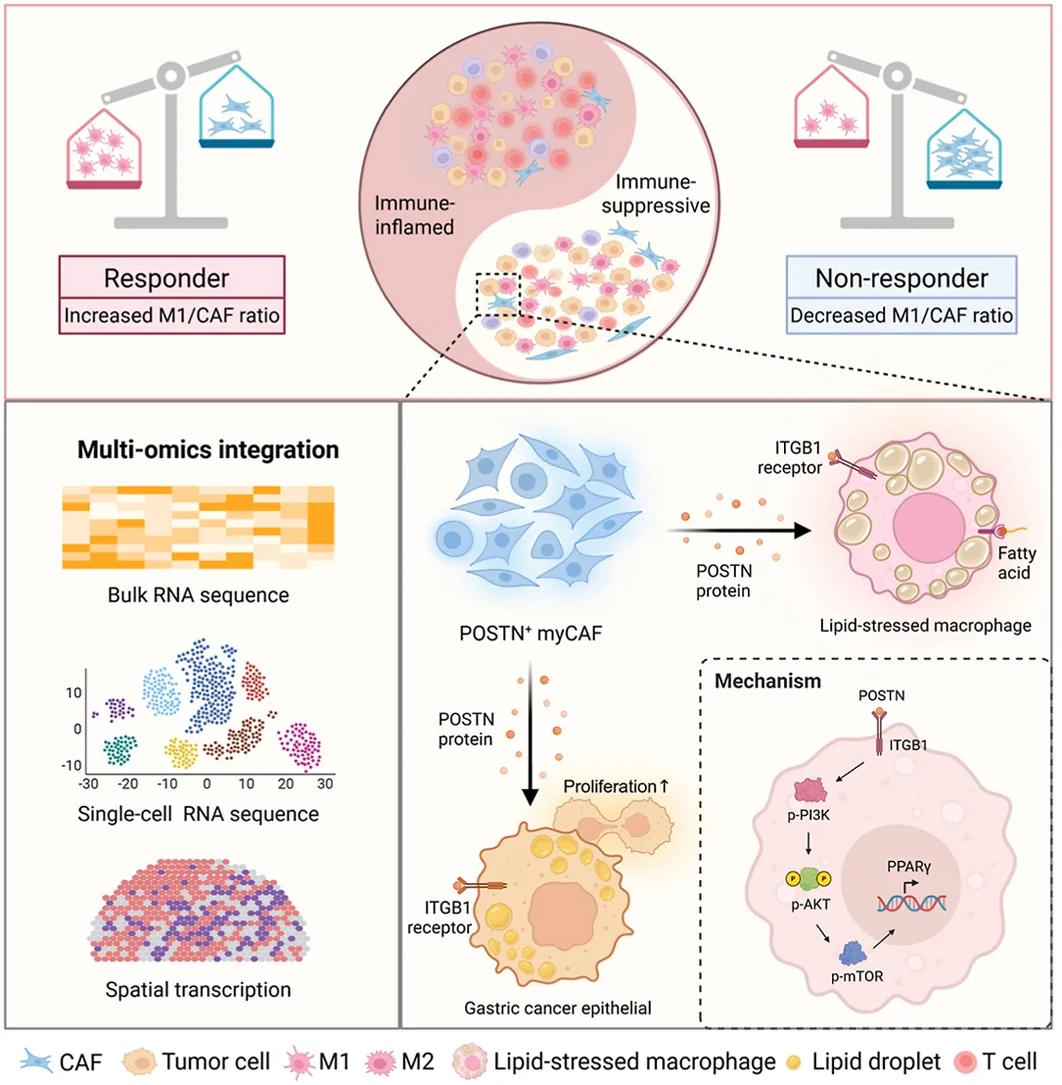

## 1 Introduction

Gastric cancer (GC) remains recognized as a prominent contributor to cancer-associated mortality on a global scale, with limited benefit from immune checkpoint blockade (ICB) despite recent therapeutic advances [1,2]. The immunosuppressive tumor microenvironment (TME), made up of stromal cells, immune cells, and extracellular matrix, serves as a pivotal factor in regulating tumor progression and therapeutic response [3,4]. Among the stromal components, cancer-associated fibroblasts (CAFs) have become crucial orchestrators of tumor biology [5]. Notably, CAF subpopulations have distinct roles in matrix remodeling, angiogenesis, and immune modulation [6,7]. For instance, myCAFs have been shown to deposit extracellular matrix proteins that enhance tumor stiffness and impede T-cell infiltration, while iCAFs secrete cytokines such as IL6 and CXCL12 to recruit immunosuppressive myeloid cells [8,9]. Although CAFs are heterogeneous and may display both tumor-promoting and tumor-restraining features across cancers [10], their enrichment in GC is predominantly associated with aggressive disease, poor prognosis, and resistance to ICB [11]. Thus, delineating the mechanisms through which CAFs shape the TME is critical for the development of effective therapeutic strategies in GC.

In parallel, metabolic reprogramming is acknowledged as a hallmark of cancer, allowing tumor cells to sustain proliferation and adapt to nutrient-deprived conditions [12]. Among the diverse metabolic alterations, lipid metabolism has gained increasing attention for its dual role in fueling tumor growth and suppressing antitumor immunity [13]. Accumulating evidence indicates that aberrant lipid metabolism skews macrophage polarization toward immunosuppressive phenotypes, thereby reinforcing an immune-evasive milieu [14]. While most studies have focused on tumor-intrinsic metabolic changes, stromal cells within the TME, including CAFs, are now recognized as active metabolic players that can modulate nutrient availability and metabolic signaling [15,16]. However, whether and how CAF-derived signals regulate lipid metabolism to influence immune cell function and immunotherapy response in GC remains poorly understood.

In this study, we integrated bulk transcriptomic, single-cell, spatial, and metabolomic analyses to comprehensively investigate the role of periostin (POSTN)-expressing CAFs in GC. We find that POSTN^+^ CAFs are enriched in immunotherapy non-responders and correlate with poor prognosis. Mechanistically, POSTN promotes lipid metabolic dysregulation and drives macrophage polarization toward an immunosuppressive phenotype, thereby fostering therapeutic non-response. Moreover, we identify integrin β1 (ITGB1) as the key receptor mediating POSTN-driven signaling and show that silencing Postn using lipid nanoparticle (LNP)-siRNA effectively remodels the TME and attenuates tumor progression.

## 2 Methods and materials

### 2.1 Clinical samples

A total of 41 primary GC tissue samples were collected from patients who underwent immunotherapy (PD-1 or PD-L1 inhibitors) at the Fudan University Shanghai Cancer Center (FUSCC). All enrolled patients underwent baseline radiological assessment, with efficacy evaluations conducted every 6 weeks in accordance with the Response Evaluation Criteria in Solid Tumors (RECIST 1.1, Fig. S1). The demographic and clinicopathological information with clinical outcomes of each patient is listed in Table S1.

### 2.2 Cell culture

HGC27 cells (Ethephon Co. Ltd, Cat# YCL-0123), AGS cells (Ethephon Co. Ltd, Cat# YCL-0348), MKN45 cells (BDBIO Co. Ltd, Cat# C5109), the murine GC cell line YTN16 established through the previously published N-methyl-N-nitrosourea (MNU) carcinogenesis model in p53 +/- mice [17], THP-1 monocytes (Ethephon Co. Ltd, Cat# YCL-0351), the human fibroblast cell line HSF (BDBIO Co. Ltd, Cat #C6028), and the murine fibroblast HCAF cell line (derived from primary mouse cancer) were all cultured in accordance with standard cell culture procedures. Detailed steps are provided in the supplementary materials.

### 2.3 Tumor challenge and treatment

YTN16 cells were injected subcutaneously into C57BL/6 mice. MKN45 cells mixed with HCAFs (3:1) were implanted into BALB/c nude mice, with POSTN-overexpressing HCAFs used for the experimental group and POSTN-NC HCAFs used as controls. Tumor size was measured with calipers at 3-day intervals, and the volume was calculated using the formula: (length × width^2^)/2, and growth curves were plotted over time. For therapeutic studies, C57BL/6 mice bearing subcutaneous YTN16 tumors were randomized when tumor volumes reached approximately 50 mm^3^. Mice were randomly assigned to four treatment groups: LNP-siNC, LNP-siPostn, anti-PD-1 plus LNP-siNC, and anti-PD-1 plus LNP-siPostn. LNP formulations were administered intravenously via the tail vein at a dose of 0.2 mg/kg siRNA every 3 days for a total of three doses. Anti-PD-1 antibody (200 μg per mouse) was administered intraperitoneally twice weekly for 2 weeks in the designated combination groups. Tumor growth was monitored throughout the treatment period, and mice were sacrificed at the experimental endpoint for collection of tumor tissues for downstream analyses. Detailed steps are provided in the supplementary materials.

### 2.4 POSTN depletion from conditioned medium

To specifically remove secreted POSTN from fibroblast-conditioned medium, an antibody-mediated immunoprecipitation strategy was employed. Briefly, conditioned medium collected from POSTN-overexpressing fibroblasts was incubated with anti-POSTN antibody overnight at 4 °C, followed by Protein A/G magnetic beads. After removal of antibody–antigen complexes, the supernatant was collected as POSTN-depleted conditioned medium. For rescue experiments, recombinant human POSTN was added back to the depleted conditioned medium before subsequent cellular assays.

### 2.5 Data retrieval and preprocessing

A total of 210 single-cell sequencing samples, along with corresponding clinical data, were used in this study. This included 123 GC tumor samples, 79 normal/tumor-adjacent tissue samples, and several additional samples from liver, peritoneum, ovary, lymph node, and peripheral blood mononuclear cells (PBMCs). The storage locations of each dataset are summarized in Table S2.

Spatial transcriptome (ST) sequencing data of adjacent-tumor, tumor, and tumor leading edge from a total of 9 primary GC samples (including 1 primary-metastasis pair, GC6) were analyzed using the Visium 10X platform and obtained from the study of Tae-Min Kim et al. [18]. Additionally, RNA-seq and clinical data for stomach adenocarcinoma (STAD) (n = 443) were downloaded from The Cancer Genome Atlas (TCGA, https://portal.gdc.cancer.gov/) and normalized. The TCGA-STAD RNA-seq dataset includes 443 samples in total, of which 32 are cancer-adjacent tissues. However, some clinical data did not correspond to RNA-seq samples, and 407 RNA-seq samples had complete clinical information.

### 2.6 Using the Scissor algorithm to identify immunotherapy response-associated single cells

The Scissor (Single-cell Identification of Subpopulations with Bulk Sample Phenotype Correlation) method was applied to pinpoint cell subsets correlated with bulk sample phenotypes [19]. Scissor-selected cells exhibited distinct molecular features linked to the biological processes of the corresponding phenotypes. In this study, the Scissor workflow integrated the phs001818 scRNA-seq dataset, the expression matrix from the FUSCC cohort, and corresponding immunotherapy response information. The response status to immunotherapy was designated as the phenotypic variable for FUSCC samples. A correlation matrix was generated to evaluate the relationship between the single-cell data from phs001818 and the bulk transcriptomic profiles of the FUSCC cohort. According to the direction of the regression coefficients, cells were categorized as Scissor-positive (Scissor^+^) or Scissor-negative (Scissor^−^), indicating positive or negative associations with immunotherapy response, respectively.

**Fig. 1:**
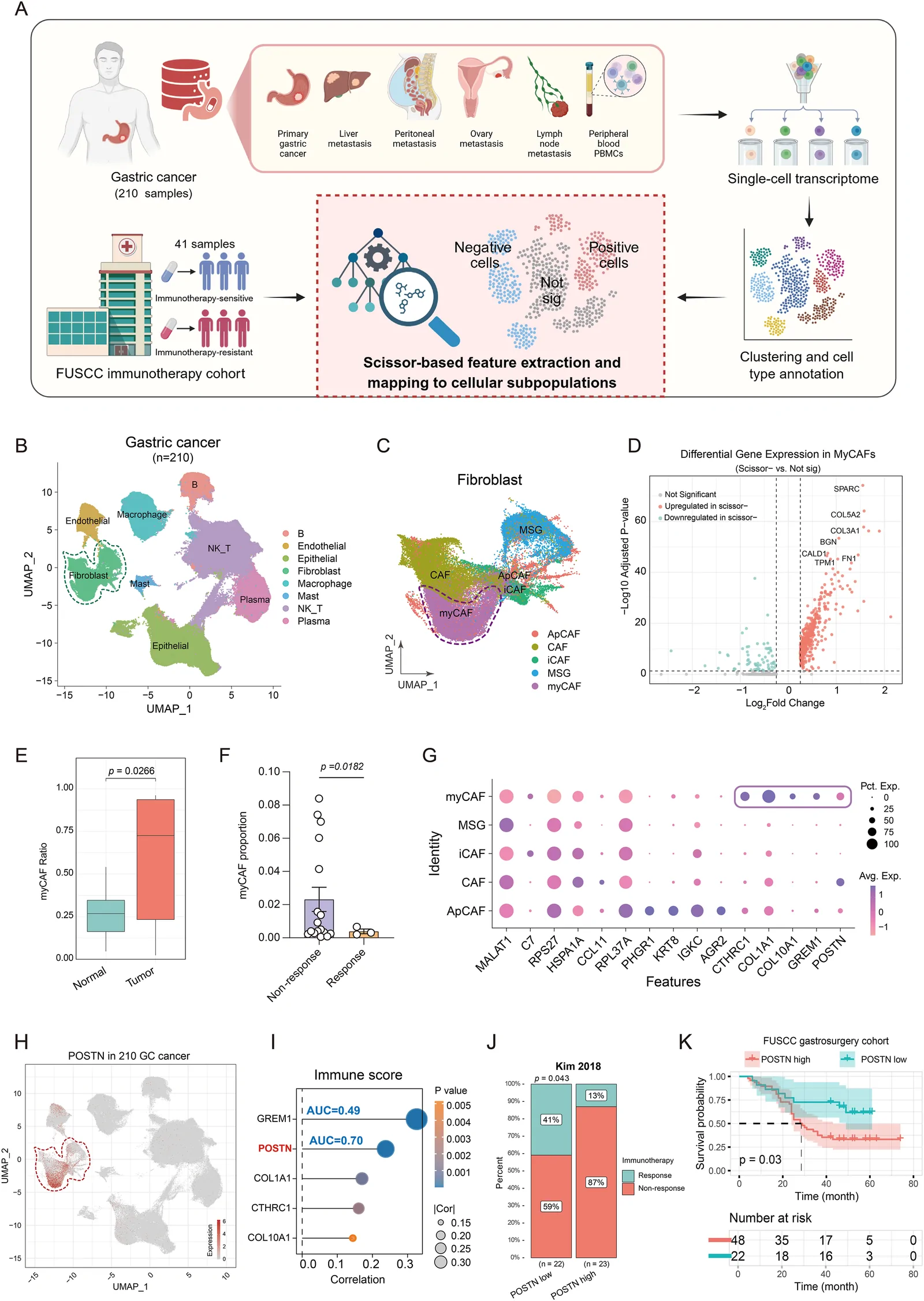
**Integrated single-cell analysis identifies POSTN as a key fibroblast-derived immunoregulatory factor in gastric cancer (GC). (A)** Schematic workflow of data processing. It involves 210 gastric cancer samples, including primary GC and metastases to liver, peritoneum, ovary, lymph nodes, as well as peripheral blood mononuclear cells (PBMCs), and transcriptional features of responders versus non-responders to immunotherapy were extracted from the FUSCC immunotherapy cohort and projected onto the integrated single-cell atlas to identify treatment-associated cell subpopulations. The workflow includes steps such as single-cell transcriptome analysis, clustering and cell type annotation, and Scissor-based feature extraction and mapping to cellular subpopulations (distinguishing positive cells, negative cells, and non-significant cells). **(B)** UMAP visualization of 210 single-cell samples with cell type annotations. **(C)** Sub-clustering and refined annotation of fibroblast subsets. **(D)** Volcano plot of differentially expressed genes between Scissor^−^ (MyCAF-enriched) and non-significant cells. Red, upregulated genes; blue, downregulated genes; gray, non-significant genes. Highlighted genes represent established MyCAF markers. **(E)** Box plot comparing the proportion of MyCAFs between normal gastric tissue and tumor tissue. **(F)** Bar plot comparing MyCAF proportions between immunotherapy responders and non-responders. **(G)** Bubble plot showing the top five marker genes of myCAFs. **(H)** UMAP plot showing the distribution of POSTN expression across cell populations. **(I)** Correlation between POSTN expression and immune score. **(J)** Comparison of immunotherapy response rates between high- and low-POSTN groups in the KIM 2018 (PRJEB25780) cohort. **(K)** Kaplan–Meier analysis of overall survival stratified by POSTN expression in the FUSCC gastric surgery cohort.

The parameter α was set to 0.2 for this analysis. The immune microenvironmental activity of Scissor-selected cells was further assessed using integrated single-cell data from a multicenter GC cohort (n = 210, Fig. 1A).

### 2.7 Spatial transcriptome (ST) analysis

Quality control (QC), clustering, and gene expression profiling of the ST dataset were performed with the R package stCancer. For mapping the spatial arrangement of single-cell subclusters, the ST and scRNA-seq matrices were integrated and jointly embedded by the CellTrek package [20]. A sparse network was constructed via random forest, followed by generation of a spot-cell similarity matrix incorporating spatial coordinates. Co-localization patterns were examined using the scoloc function, while co-expression intensity was quantified by Kullback-Leibler divergence (KLD), with larger KLD values reflecting stronger co-localization. Deconvolution analysis using CARD identified large co-localizing subclusters, followed by distributional correlation analysis [21]. Cottrazm was applied to delineate malignant (Mal), boundary (Bdy), and non-malignant (nMal) regions, while cell enrichment was evaluated through a deconvolution approach [22]. Spatial pathway enrichment analyses were conducted using GSVA, and multiple testing correction was performed using the Benjamini–Hochberg method.

### 2.8 RNA isolation, cDNA synthesis, and RT-qPCR

Primer sequences are provided in Tables S3–S5. Detailed steps are provided in the supplementary materials.

### 2.9 His pull-down assay

Detailed steps are provided in the supplementary materials.

### 2.10 Macrophage-T cell co-culture assay

Detailed steps are provided in the supplementary materials.

### 2.11 siRNA library screening by parallel transfection

siRNA-mediated knockdown of nineteen integrin genes (ITGA1, ITGA2, ITGA3, ITGA4, ITGA5, ITGA6, ITGA7, ITGAE, ITGAL, ITGAM, ITGAV, ITGAX, ITGB1, ITGB2, ITGB3, ITGB4, ITGB5, ITGB7, ITGB8) was performed in parallel using a 96-well plate format. The corresponding siRNA sequences are provided in Table S6. Detailed steps are provided in the supplementary materials.

### 2.12 Multiplex immunofluorescence (mIF) staining

Tissue microarrays (TMAs) were constructed by Shanghai Outdo Biotech Company (Shanghai, China) according to standard protocols. Sections were incubated sequentially with primary antibodies against POSTN, ITGB1, PPARG, CD163, and α-SMA (dilutions and clones listed in Table S7). Detailed steps are provided in the supplementary materials.

### 2.13 siRNA-LNP formulation

LNPs were prepared using a microfluidic-based method (MicroANNA system) with four lipid components: SM-102, cholesterol, DSPC, and DMG-PEG, dissolved in anhydrous ethanol. The lipids were combined at a molar ratio of 50:38.5:10:1.5 (SM-102:cholesterol:DSPC:DMG-PEG), respectively. For targeted LNPs, DMG-PEG was partially replaced with DSPE-PEG-cRGD at a 20% molar percentage. The lipid mixture (organic phase) was injected at a flow rate ratio of 1:3 against an aqueous phase containing siRNA in 10× buffer and DEPC water. The resulting nanoparticles were collected, buffer-exchanged using a 100 kDa centrifugal filter, and purified via three rounds of centrifugation at 1200 × g for 45 min. Detailed experimental procedures are provided in the supplementary materials.

### 2.14 Flow cytometry of tumor-infiltrating immune cells in mice

Live CD45^+^ leukocytes were selected for analysis, with CD3^+^CD8a^+^ and CD3^+^CD4^+^ populations gated as tumor-infiltrating T cells, CD25^+^ cells within the CD4^+^ compartment identified as activated CD4^+^ T cells, and CD11b^+^F4/80^+^ cells defined as macrophages, which were further subdivided into M1 (CD86^+^) and M2 (CD206^+^) subsets. Detailed steps are provided in the supplementary materials.

### 2.15 Untargeted metabolomics

For untargeted metabolomics of mouse subcutaneous xenograft tumors, 100 mg of tissue was pulverized under liquid nitrogen and resuspended in prechilled 80% methanol. Samples were vortexed, incubated on ice for 5 min, and centrifuged at 15,000×g, 4 °C for 20 min. The supernatant was diluted to 53% methanol with LC-MS grade water, transferred to a fresh tube, centrifuged again under identical conditions, and analyzed using an LC-MS/MS system. Chromatographic separation employed a Hypersil Gold column with a 12-min linear gradient at 0.2 mL/min between eluent A (0.1% formic acid in water) and eluent B (methanol). The mass spectrometer operated in positive/negative mode with spray voltage 3.5 kV, capillary temperature 320 °C, sheath gas 35 psi, auxiliary gas 10 L/min, S-lens RF 60, and auxiliary gas heater 350 °C. Detailed steps are provided in the supplementary materials.

### 2.16 Data analysis

Statistical analyses were performed using GraphPad Prism 9 and R software. Data are presented as mean ± SD unless otherwise indicated. Comparisons between two groups were performed using appropriate parametric or nonparametric tests, whereas multiple-group comparisons were analyzed using ANOVA or Kruskal-Wallis tests with corresponding post hoc corrections when applicable. Survival analyses were performed using the Kaplan-Meier method with log-rank testing. For high-dimensional omics analyses, multiple testing corrections were applied where appropriate. Detailed statistical procedures, including statistical tests, effect size estimation, and correction methods for multi-omics analyses, are provided in the Supplementary Methods. A two-sided P value < 0.05 was considered statistically significant.

## 3 Results

### 3.1 Identification of POSTN^+^ CAFs associated with immunotherapy non-response through integration of 210 single-cell transcriptomes

Distinct subsets of infiltrating immune cells exhibit context-dependent effects on tumor progression [23]. To comprehensively investigate the GC microenvironment in relation to immunotherapy response, we constructed a data analysis workflow (Fig. 1A) that integrated 210 single-cell samples collected from GC patients. In parallel, bulk transcriptomic profiles and corresponding clinical outcomes from 41 GC patients treated with immunotherapy at FUSCC (FUSCC immunotherapy RNA-seq cohort, n = 41) were incorporated for feature extraction.

From the 210 single-cell samples, eight major cell subtypes were identified: B cells, endothelial cells, epithelial cells, fibroblasts, macrophages, mast cells, NK_T cells, and plasma cells (Fig. 1B). Application of the Scissor algorithm then mapped therapeutic response to the single-cell landscape and revealed that fibroblasts were negatively correlated with immunotherapy efficacy (Fig. S2a–c). Fibroblast subpopulation re-annotation based on canonical markers showed that Scissor-negative fibroblasts corresponded to myofibroblastic CAFs (myCAFs) (Fig. 1C, S2d–f, S3a–b). Differential expression profiling revealed that myCAFs were enriched for pro-tumorigenic genes such as SPARC, COL5A2, COL3A1, and CALD1 (Fig. 1D), all of which have been implicated in cancer progression [[24], [25], [26], [27]]. MyCAFs displayed elevated infiltration within tumor tissues (Fig. 1E), with marked heterogeneity (Fig. S3c), and were preferentially enriched in non-responders to immunotherapy (Fig. 1F). Furthermore, myCAF infiltration positively correlated with TIDE scores, indicating impaired response to immune checkpoint inhibitors (Fig. S3d).

To further characterize myCAFs, we identified the top five associated genes (Fig. 1G). Among these, POSTN showed a fibroblast-specific expression pattern (Fig. 1H). Although GREM1 had the strongest correlation with immune score in the Kim2018 (PRJEB25780) cohort, its predictive accuracy for immunotherapy response was limited (AUC = 0.49, Fig. 1I, S3e). In contrast, POSTN maintained a strong correlation with immune score and achieved higher predictive accuracy (AUC = 0.70, Fig. 1I, S3f). Consistently, high POSTN expression was associated with markedly reduced immunotherapy response rates (Fig. 1J).

To further determine the cellular origin of POSTN within the gastric cancer microenvironment, we integrated multiple independent public and in-house single-cell RNA sequencing datasets. Across all datasets, POSTN-expressing cells were overwhelmingly enriched in fibroblast populations, whereas only minimal expression was detected in epithelial or immune compartments (Fig. S4a–g). Consistently, fibroblast cell lines expressed substantially higher POSTN mRNA levels than gastric cancer cell lines, and secreted markedly greater amounts of POSTN protein into conditioned medium (Fig. S4h–i). Computational deconvolution further demonstrated that POSTN expression positively correlated with CAF abundance and inversely correlated with tumor purity (Fig. S4j–k). Moreover, CellChat analysis identified fibroblasts as the dominant source of POSTN-mediated ligand–receptor interactions within the tumor microenvironment (Fig. S4l). Collectively, these findings establish fibroblasts as the predominant cellular source of POSTN in gastric cancer.

We next explored the potential of POSTN as a biomarker for predicting immunotherapy efficacy. At both the single-cell and bulk levels, POSTN expression was strongly correlated with stromal and ESTIMATE scores, suggesting its value in assessing immune status (Fig. S5a–b). In the independent cohort PRJEB25780, patients with poor prognosis (PD/SD) exhibited higher POSTN expression (Fig. S5c). Similarly, high POSTN expression was associated with shorter overall survival (OS) in the FUSCC gastrosurgery cohort (Fig. 1K). Moreover, in the TCGA-STAD cohort, patients with high POSTN + CAF infiltration demonstrated shorter OS and progression-free survival (PFS) (Fig. S5d–e). Collectively, these findings indicate that increased POSTN^+^ CAF infiltration is associated with adverse prognosis in GC patients.

### 3.2 POSTN enhances tumor cell proliferation and shapes an immunosuppressive TME

**Fig. 2:**
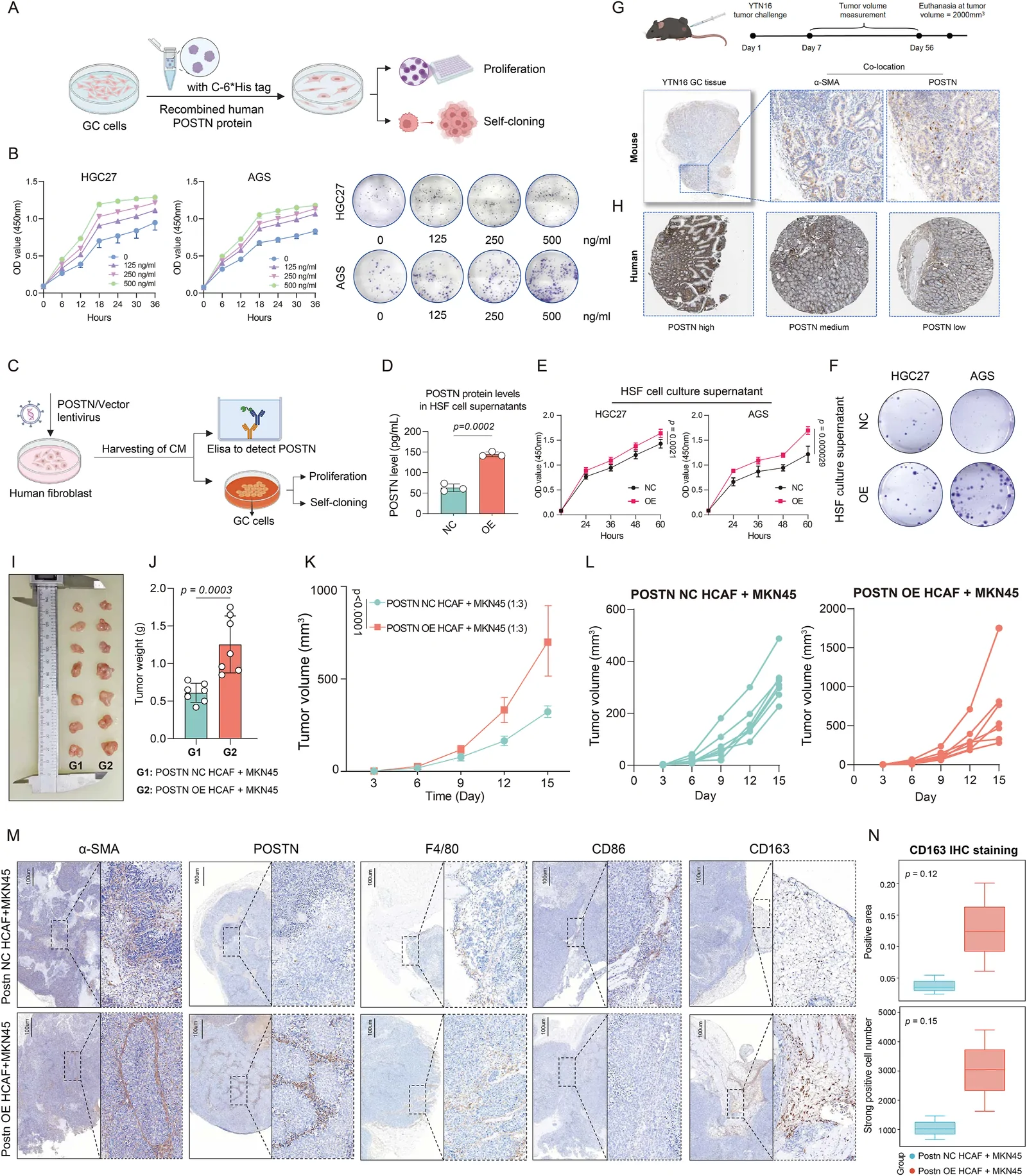
**POSTN enhances GC cell proliferation and promotes tumor growth *in vitro and in vivo*. (A)** Schematic diagram of experimental design. Rh POSTN (C-6×His tag) was added to GC cell cultures, followed by assessment of proliferation and colony formation. **(B)** Proliferation curves of HGC27 and AGS cells treated with different concentrations of rhPOSTN; right panels show colony formation assays under increasing POSTN concentrations. **(C)** Schematic of fibroblast POSTN overexpression via lentiviral transduction, followed by conditioned medium (CM) collection and functional testing in GC cells. **(D)** ELISA quantification of POSTN protein levels in CM from control (NC) and POSTN-overexpressing (OE) fibroblasts (P = 0.002). **(E)** Proliferation curves of HGC27 (left) and AGS (right) cells cultured in CM from NC or POSTN-OE fibroblasts. **(F)** Colony formation assays of HGC27 and AGS cells cultured in CM from NC or POSTN-OE fibroblasts. **(G)** Schematic of in vivo tumorigenesis model. GC cells were implanted into mice, followed by tumor monitoring and IHC staining for POSTN. **(H)** Representative IHC images of POSTN expression in human GC specimens categorized as high, medium, or low. **(I)** Photographs of excised tumors from different experimental groups (n = 7 mice per group). **(J)** Tumor weights of excised tumors from NC and POSTN-OE groups (P = 0.003). **(K-L)** Tumor growth curves over time in NC versus POSTN-OE groups. **(M)** Representative IHC images of α-SMA, Postn, F4/80, Cd86, and Cd163 in tumors from NC and POSTN-OE groups. **(N)** Quantification of Cd163 immunohistochemical staining, including the percentage of positive staining area (upper) and the number of strongly stained Cd163-positive cells (lower). Unless otherwise indicated, data are presented as mean ± SD from three independent biological replicates (n = 3).

Following the identification of POSTN as a potential predictive marker for immunotherapy response, we sought to delineate its functional contributions to tumor cell biology. To investigate its direct effects, GC cells were treated with increasing concentrations of recombinant POSTN protein (rhPOSTN) (Fig. 2A). This treatment resulted in a dose-dependent enhancement of both cell proliferation and clonogenic capacity, a key indicator of tumorigenic potential (Fig. 2B). Based on these dose–response experiments, 500 ng/mL rhPOSTN was selected for all subsequent in vitro studies unless otherwise specified, as this concentration consistently elicited robust biological effects without evidence of overt cytotoxicity. Consistent with these findings, overexpression of POSTN within GC cells themselves accelerated their proliferative rate (Fig. S6a–f).

To further validate the tumor-promoting role of POSTN within the TME, GC cells were exposed to conditioned medium from POSTN-overexpressing fibroblasts (Fig. 2C–D). This treatment recapitulated the pro-tumorigenic phenotypes, resulting in markedly increased proliferation and clonogenic capacity (Fig. 2E–F). We next investigated the spatial distribution of POSTN in human gastric cancer tissues using spatial transcriptomic datasets. POSTN expression was predominantly confined to fibroblast-rich regions, where it exhibited substantial spatial overlap with the canonical fibroblast markers FAP, COL1A1, and ACTA2, while remaining largely absent from EPCAM-positive epithelial compartments (Fig. S7a–c). This stromal localization was further corroborated by multiplex immunofluorescence, which demonstrated extensive co-localization of POSTN with α-SMA-positive fibroblasts (Fig. S7d). Similar findings were obtained in a murine GC model, where POSTN was likewise enriched in stromal fibroblasts (Fig. 2G). In parallel, clinical gastric cancer specimens displayed marked intertumoral heterogeneity in POSTN expression (Fig. 2H). To determine whether the cellular origin of POSTN influences its biological activity, we directly compared tumor cell-derived and CAF-derived POSTN after normalizing protein levels. Both sources exerted broadly comparable effects on GC cell proliferation and clonogenic growth (Fig. S8a), suggesting that the biological activity of POSTN is largely independent of its cellular origin. Nevertheless, because POSTN is predominantly localized to stromal fibroblasts in both human and murine tumors, CAFs are likely to represent its principal physiological source within the GC microenvironment.

We next extended our investigation to an in vivo setting. Tumors supported by CAFs engineered to overexpress POSTN exhibited significantly accelerated growth kinetics. Notably, the pro-tumorigenic effects were more pronounced in the fibroblast-specific POSTN overexpression models compared to tumor cell-specific models (Fig. 2I–L), highlighting the critical importance of stromal-derived POSTN. To further exclude potential confounding effects arising from the mixed-species xenograft model, we established a species-matched human stromal model by co-implanting MKN45 cells with control or POSTN-overexpressing human fibroblasts (HSFs). Consistent with our original findings, POSTN-overexpressing HSFs significantly accelerated tumor growth and increased tumor volume compared with control fibroblasts (Fig. S8b–c), further supporting the tumor-promoting role of stromal-derived POSTN. Beyond driving proliferation, POSTN significantly remodeled the immune landscape of the TME. Immunohistochemical analysis of tumor tissues from the aforementioned mouse models demonstrated distinct macrophage infiltration patterns. Specifically, tumors supported by POSTN-overexpressing CAFs displayed a significant increase in the infiltration of M2-polarized macrophages, which are widely associated with immunosuppressive functions (Fig. 2M).

Collectively, these findings demonstrate that POSTN facilitates GC progression through a dual mechanism: it directly enhances the proliferative and clonogenic capacities of tumor cells, and it indirectly fosters an immunosuppressive TME, characterized by the enrichment of pro-tumorigenic M2 macrophages.

### 3.3 POSTN drives lipid metabolic remodeling and promotes oxidative stress

**Fig. 3:**
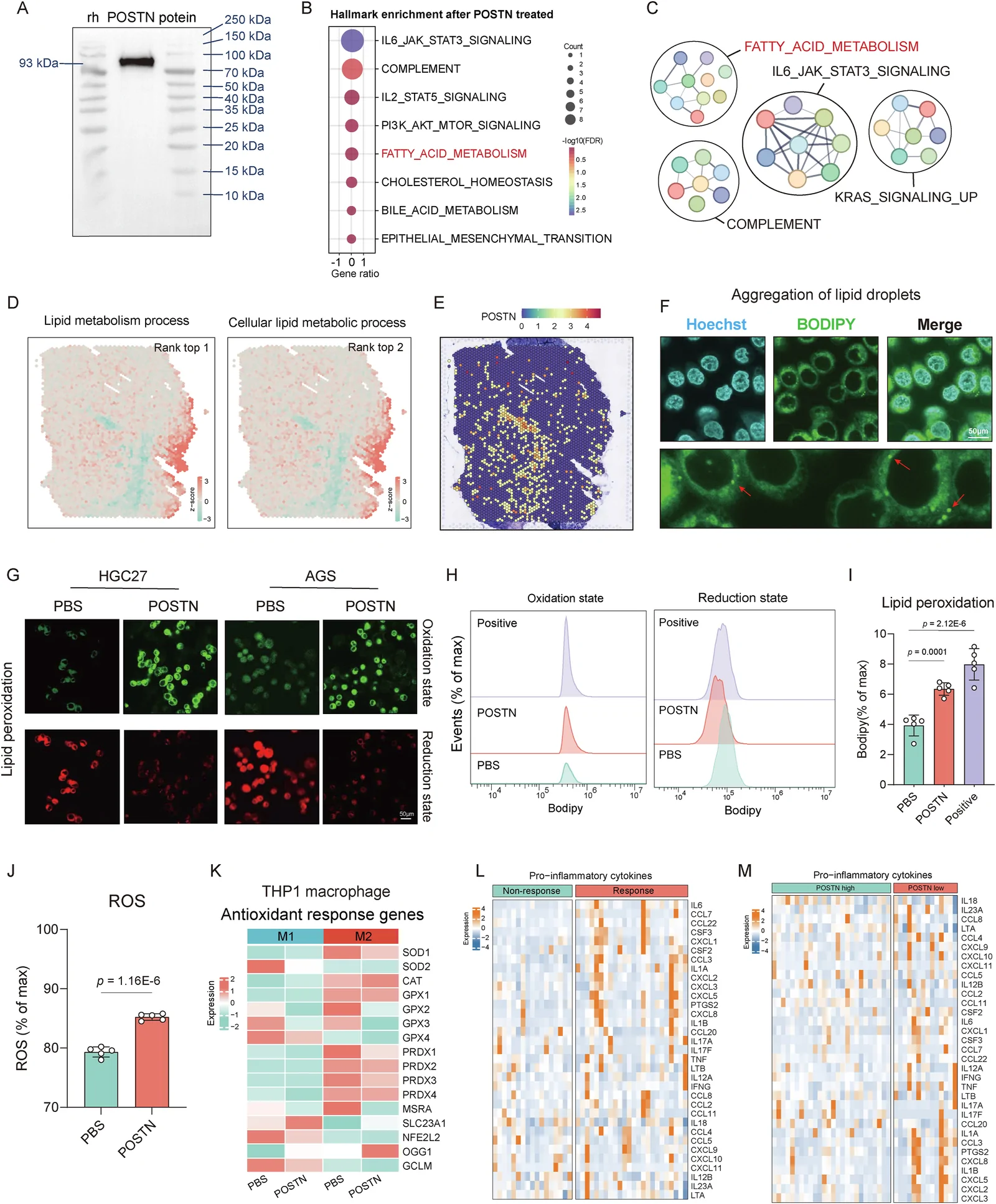
**POSTN regulates lipid metabolic remodeling and redox-associated responses in gastric cancer cells and macrophages. (A)** Western blot analysis of recombinant human POSTN (rh POSTN) protein, with a band at approximately 93 kDa. **(B)** Hallmark pathway enrichment analysis after POSTN treatment. The horizontal axis represents the gene ratio, and the vertical axis lists enriched pathways. **(C)** Network visualization of enriched pathways and their interconnections, showing the regulatory network involved after POSTN treatment. **(D)** Spatial mapping of lipid metabolism pathway enrichment scores in GC tissue sections. **(E)** Localization of POSTN expression in GC slices. **(F)** Accumulation of intracellular lipid droplets in GC cells. The lower panel is a magnified view, with red arrows pointing to aggregated lipid droplets. **(G–I)** Increased levels of lipid peroxides upon POSTN treatment, visualized by confocal microscopy (G), detected by flow cytometry (H), and quantification (I, n = 5). **(J)** Quantitative analysis of reactive oxygen species (ROS) by flow cytometry (n = 5). **(K)** Heatmap of antioxidant response gene expression in macrophages. **(L–M)** Elevated expression of pro-inflammatory cytokines in immunotherapy responders (L) and in patients with low POSTN expression (M) of TCGA-STAD cohort. Data are presented as mean ± SD.

Since POSTN was shown to enhance tumor cell proliferation, we next examined the mechanisms underlying this effect. Metabolic reprogramming represents a key driver of tumor growth by supplying biosynthetic precursors, energy, and signaling molecules required for sustained proliferation [28,29]. To determine whether POSTN influences metabolic pathways, we treated GC cells with recombinant POSTN protein, confirming its purity by immunoblotting (Fig. 3A). Transcriptomic profiling followed by enrichment analysis revealed significant alterations in multiple metabolic processes, with fatty acid metabolism ranking among the most prominently enriched pathways (Fig. 3B). Network analysis of enriched pathways further illustrated extensive interactions among metabolism-related processes (Fig. 3C). Consistently, spatial transcriptomic data from human GC tissues identified lipid metabolism-related pathways as the top enriched categories (Fig. 3D). Notably, regions with high lipid metabolism scores were predominantly localized to epithelial compartments, whereas fibroblast-rich areas displayed strong POSTN expression (Fig. 3E, S9a–f), suggesting that POSTN secreted by CAFs may act in a paracrine manner to reprogram lipid metabolism in adjacent tumor cells. Furthermore, areas with high POSTN expression were populated not only by fibroblasts but also by macrophages (Fig. 3E, S9d), prompting us to investigate whether POSTN-induced metabolic alterations extend to these key immune cells within the TME.

Fatty acid metabolism, which requires more oxygen and results in intracellular lipid droplet accumulation (Fig. 3F), generates higher levels of reactive oxygen species (ROS) than glucose metabolism, and causes a notable decrease in mitochondrial membrane potential [[30], [31], [32]]. To investigate this, we treated GC cells with rhPOSTN, which promoted the accumulation of lipid oxidation products (Fig. 3G–I). Moreover, the POSTN-treated group exhibited elevated ROS levels and reduced mitochondrial membrane potential compared to the control (Fig. 3J, S9g-i). These findings collectively suggest an underlying disruption in lipid metabolism. To further verify that these effects were specifically mediated by POSTN, we performed complementary loss-of-function and rescue experiments. Knockdown of POSTN significantly attenuated tumor cell clonogenic capacity, whereas supplementation with recombinant POSTN effectively restored colony formation (Fig. S10a). Likewise, POSTN depletion reduced intracellular lipid oxidation and oxidative stress, both of which were rescued by rhPOSTN treatment, as demonstrated by flow cytometric quantification and fluorescence imaging (Fig. S10b–e).

To further establish the specificity of fibroblast-derived POSTN, we depleted POSTN from conditioned medium using an antibody-mediated immunoprecipitation strategy (Fig. S10f). Removal of POSTN markedly diminished the ability of conditioned medium to induce intracellular lipid oxidation, whereas reconstitution with recombinant POSTN largely restored this phenotype (Fig. S10g–h).

Given the spatial proximity of POSTN^+^ niches to macrophages, we next assessed the oxidative state in these immune cells. Application of rhPOSTN protein to M1/M2 macrophages revealed a more pronounced antioxidant alteration in M2 macrophages compared with M1 macrophages (Fig. 3K). Furthermore, cytokine expression patterns differed between immunotherapy responder and non-responder groups, as well as between POSTN-high and POSTN-low states, suggesting broader immune microenvironment remodeling associated with POSTN-related metabolic and redox alterations (Fig. 3L and M).

Collectively, these findings demonstrate that POSTN drives profound lipid metabolic reprogramming, resulting in excessive lipid peroxidation and oxidative stress in GC cells. Importantly, these redox alterations were not restricted to tumor cells but also extended to macrophages, particularly the M2 subset, suggesting that POSTN-mediated metabolic remodeling may contribute to the establishment of an immunosuppressive TME.

### 3.4 POSTN^+^ CAFs drive lipid-stressed macrophage polarization via lipid metabolism dysregulation

**Fig. 4:**
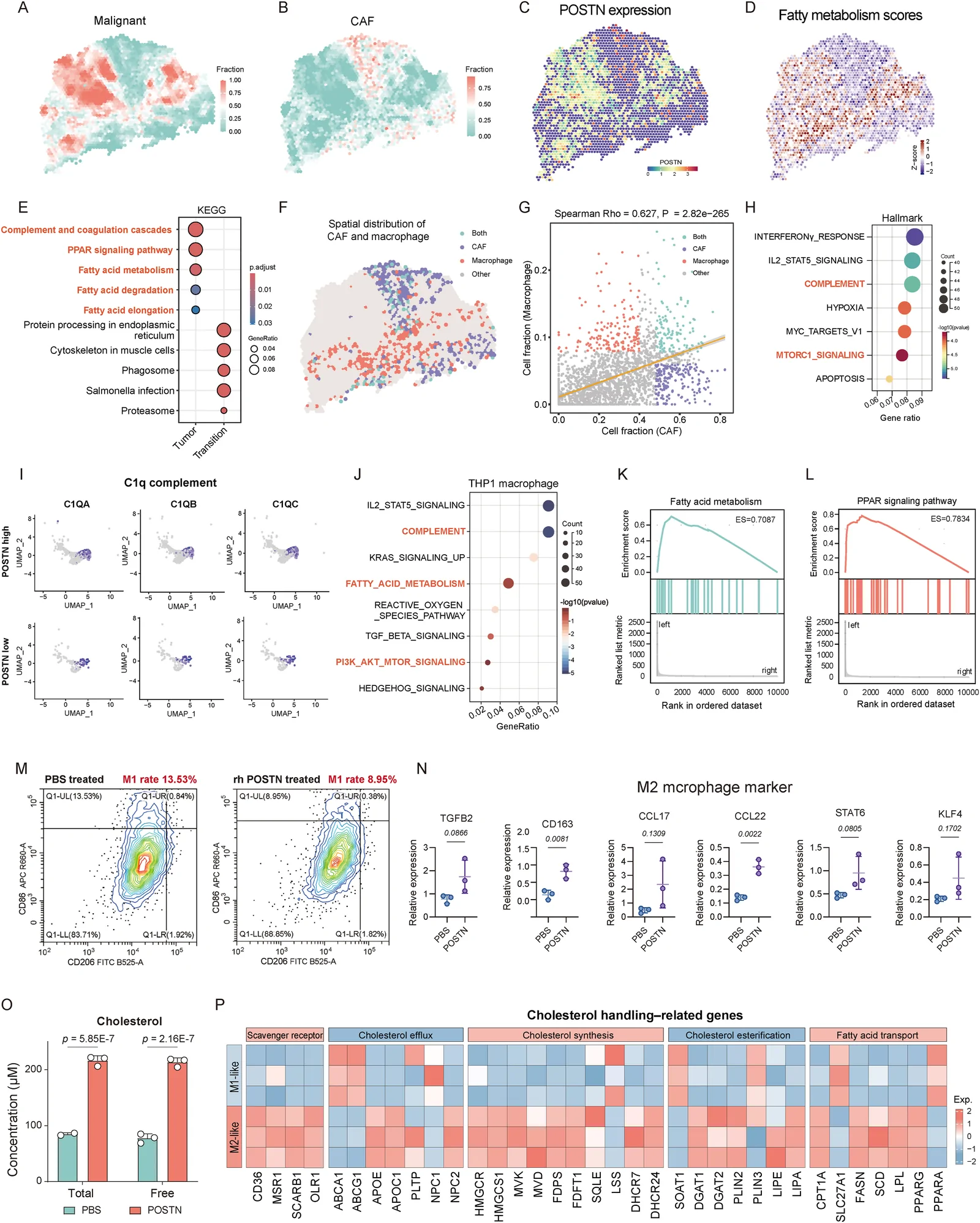
**POSTN reprograms macrophage polarization and lipid metabolism in the GC microenvironment. (A–B)** Spatial distribution of malignant cells (A) and cancer-associated fibroblasts (CAFs) (B) in GC tissue sections, with color intensity indicating cell density. **(C)** Expression pattern of POSTN across the tissue, visualized via a color scale. **(D)** Spatial distribution of lipid metabolism pathway scores across tissue slices, with color variation representing score levels. **(E)** Regional enrichment of distinct gene signatures within GC sections. **(F)** Spatial overlap of CAFs and macrophages in the tissue. Blue dots indicate the spatial co-localization of CAFs and macrophages, purple dots represent CAFs, red dots represent macrophages, and gray dots represent other cell types. **(G)** Scatter plot illustrating the correlation between CAF cell fraction and macrophage density, with a Spearman correlation coefficient of 0.627. **(H)** Bar plot of Hallmark pathway enrichment of macrophages in GC tissues. **(I)** Expression of C1q complement component (C1QA, C1QB, C1QC) in macrophages from POSTN-high versus POSTN-low samples. **(J)** Dot plot of enriched pathways in differential genes of THP1 macrophages treated with rhPOSTN or not. **(K–L)** Upregulation of fatty acid metabolism (K) and PPAR signaling (L) pathways in rhPOSTN-treated THP1 macrophages. **(M)** Flow cytometry plots of M1 macrophage (CD86^+^) frequency in PBS-treated (left) and rhPOSTN-treated (right) groups. **(N)** Increased expression of M2 markers in rhPOSTN-treated THP1 macrophages. **(O)** Quantification of intracellular total cholesterol and free cholesterol levels in macrophages treated with PBS or rhPOSTN. **(P)** Heatmap showing expression changes of cholesterol handling-related genes across macrophage polarization states (M1-like, M2-like) following rhPOSTN treatment, including upregulation of lipid or cholesterol uptake genes (CD36, MSR1, SCARB1, and OLR1) and dysregulation of cholesterol efflux-associated genes (ABCA1 and ABCG1). Unless otherwise indicated, data are presented as mean ± SD from three independent biological replicates (n = 3).

Spatial transcriptomics enables precise mapping of gene expression within tissue sections, providing insights into cellular localization and interactions within the TME [31]. Here, we delineated the spatial distribution of tumor cells, CAFs, and macrophages in GC tissues (Fig. 4A and B, S11a). While POSTN was expressed at detectable levels in tumor cells, its high expression was predominantly localized within CAF-rich regions (Fig. 4C, S11b). Notably, tumor regions exhibited elevated fatty acid metabolism scores, suggesting heightened lipid metabolic activity in GC cells (Fig. 4D). Regional segmentation analysis further revealed that DEGs in tumor regions were primarily enriched in complement-related pathways (Complement and coagulation cascades) and lipid metabolism pathways, including the PPAR signaling pathway, fatty acid metabolism, fatty acid degradation, and fatty acid elongation (Fig. 4E). Given our previous bulk-level findings of a strong positive correlation between POSTN and macrophages, we further assessed the spatial co-localization of CAFs and macrophages. The significant correlation (R = 0.627) suggests substantial spatial overlap, indicating potential direct interactions between these two cell populations (Fig. 4F–G, S11c-d). Consistent results were observed in the TCGA-STAD cohort, where POSTN expression correlated positively with multiple immune cell subsets, most notably macrophages (Fig. S11e–f). Furthermore, considering that POSTN^+^ CAFs were more prevalent in patients with poor immunotherapy response (Fig. S3d), we hypothesized that macrophages would also be enriched in non-responders. Supporting this, expression of colony-stimulating factor 1 (CSF1), a marker of macrophage activation, was significantly elevated in non-responders compared to responders (Fig. S11g).

To characterize macrophage heterogeneity associated with POSTN, we re-clustered macrophages from both the in-house and multicenter single-cell datasets. Consistent macrophage subpopulations were identified across datasets based on canonical marker expression (Fig. S12a–d). Among the identified macrophage subpopulations, APOE^+^ macrophages, a subset characterized by the expression of apolipoprotein E, a key regulator of lipid transport and cholesterol homeostasis, were significantly enriched in POSTN-high tumors compared with POSTN-low tumors (Fig. S12e). Given the established role of APOE in macrophage lipid metabolism and its association with immunosuppressive immunosuppressive tumor-associated macrophages (TAMs), these findings suggest that POSTN-driven lipid metabolic reprogramming may promote the accumulation of lipid-laden macrophages within the TME. Differential expression analysis further demonstrated that macrophages from POSTN-high tumors preferentially activated lipid metabolism, oxidative stress, and immune suppressive pathways (Fig. S12f). Importantly, Oil Red O staining demonstrated marked intracellular lipid accumulation following rhPOSTN stimulation or treatment with conditioned medium derived from POSTN-overexpressing fibroblasts (Fig. S12g), further supporting the lipid-stressed phenotype induced by POSTN.

To further investigate the impact of POSTN^+^ CAFs on macrophage phenotype, we analyzed macrophage-specific DEGs in samples with high versus low POSTN expression. Complement-related pathways were enriched, along with the mTORC signaling pathway (Fig. 4H). Consistently, we observed pronounced upregulation of complement components C1QA, C1QB, and C1QC in the high-POSTN group (Fig. 4I). Notably, C1QA has been implicated in lipid metabolic regulation by inducing inflammatory responses in adipocytes and altering fatty acid synthesis and degradation [32]. To further characterize the spatial organization of POSTN-associated macrophage remodeling, we integrated cell-type deconvolution with spatial transcriptomic analysis. Fibroblasts and macrophages exhibited adjacent spatial distributions across gastric cancer tissue sections (Fig. S13a–b). Regions enriched for POSTN^+^ CAF signatures spatially coincided with areas exhibiting elevated macrophage fatty acid metabolism scores (Fig. S13c–d), providing additional evidence that POSTN-rich stromal niches are closely associated with macrophage metabolic reprogramming in situ. In line with this, macrophages treated with rhPOSTN showed enrichment of fatty acid metabolism pathways, further accompanied by activation of PI3K-AKT-mTOR signaling (Fig. 4J and K), as well as induction of the lipid metabolic regulator PPAR signaling (Fig. 4L). Functionally, rhPOSTN treatment reduced the proportion of M1 macrophages, increased M2 markers, and decreased M1 markers (Fig. 4M − N, S14a). Similarly, conditioned medium from POSTN-overexpressing fibroblasts showed diminished M1 induction (Fig. S14b). Bulk RNA sequencing of rhPOSTN-treated macrophages confirmed these findings (Fig. S14c).

Beyond polarization, POSTN also induced profound metabolic remodeling in macrophages. rhPOSTN treatment markedly increased lipid peroxidation (Fig. S14d) and intracellular oxidative status (Fig. S14e), accompanied by significant accumulation of both total and free cholesterol (Fig. 4O). Moreover, expression of cholesterol handling–related genes was substantially altered, characterized by upregulation of lipid and cholesterol uptake receptors, including CD36, MSR1, SCARB1, and OLR1, together with dysregulation of the cholesterol efflux transporters ABCA1 and ABCG1 (Fig. 4P). Collectively, these findings indicate that POSTN promotes extensive lipid and cholesterol metabolic reprogramming in macrophages.

To determine whether macrophage reprogramming was specifically dependent on fibroblast-derived POSTN, we performed complementary depletion and rescue experiments. Macrophages exposed to conditioned medium from POSTN-knockdown fibroblasts exhibited significantly reduced expression of M2-associated markers, whereas supplementation with recombinant POSTN effectively restored the polarization phenotype (Fig. S14f). Similarly, depletion of POSTN from fibroblast-conditioned medium by antibody-mediated immunoprecipitation markedly attenuated M2-like polarization, and reconstitution with recombinant POSTN largely rescued this effect (Fig. S14g).

Together, these data provide convergent evidence that fibroblast-derived POSTN is both sufficient and functionally required for driving macrophage lipid metabolic remodeling and M2-like polarization.

### 3.5 POSTN engages ITGB1 to trigger PI3K/AKT/mTOR-PPARγ signaling, coupling lipid metabolism to immune suppression in the TME

**Fig. 5:**
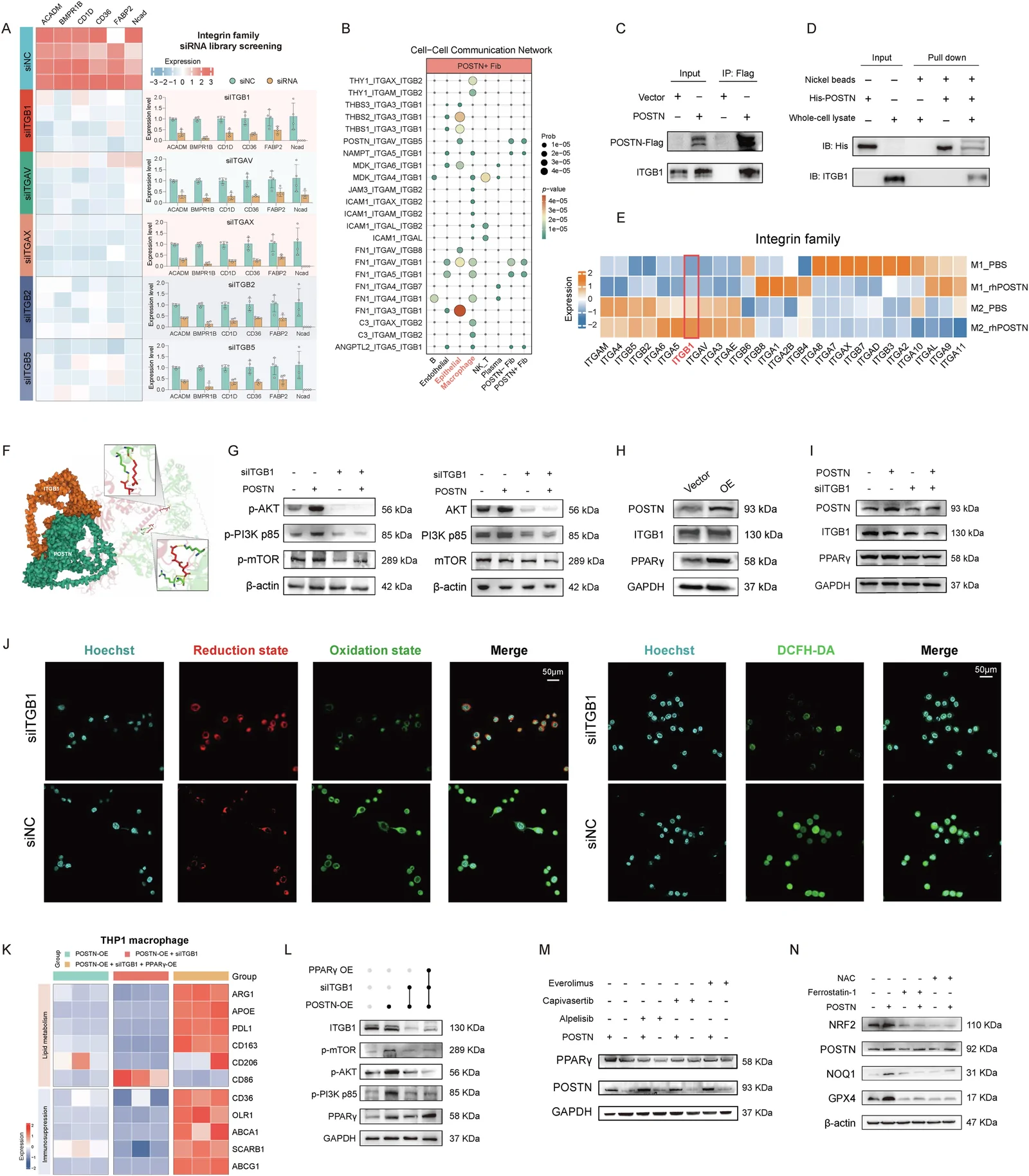
**ITGB1 mediates POSTN-driven lipid metabolic reprogramming in tumor and macrophage cells. (A)** Heatmap (left) showing expression of lipid metabolism–related genes (ACADM, BMPR1B, CD1D, CD36, FABP2) and EMT marker N-cadherin in cells transfected with control siRNA (siNC) or siRNAs targeting integrin subunits (ITGB1, ITGAV, ITGAX, ITGB2, ITGB5). Bar plots (right) depict relative expression levels of these genes under the same conditions. **(B)** Cell–cell communication analysis highlighting ligand–receptor interactions involving POSTN^+^ fibroblasts. Circle size represents interaction probability, and color denotes statistical significance. **(C)** Co-immunoprecipitation (Co-IP) confirming interaction between POSTN and ITGB1. IP was performed with Flag antibody in cells transfected with POSTN-Flag or Vector, followed by IB for POSTN-Flag and ITGB1. Input: total cell lysate. **(D)** Pull-down assay showing direct binding of His-tagged POSTN to ITGB1 in macrophage lysates. **(E)** Heatmap of integrin family gene expression in M1 macrophages (PBS vs. rhPOSTN treatment) and M2 macrophages (PBS vs. rhPOSTN treatment). Color scale indicates z-score normalized expression. **(F)** Structural modeling of POSTN–ITGB1 interaction. Left: overall interaction interface (ITGB1, orange; POSTN, green). Right: close-up of the binding pocket with key interacting residues highlighted. **(G)** Western blot analysis of PI3K/AKT/mTOR signal in GC cells with empty vector (−) or POSTN-overexpressing vector (+), showing total and phosphorylated PI3K p85, AKT, and mTOR. **(H)** Western blot showing POSTN, ITGB1, and PPARγ expression in cells transfected with Vector or POSTN-OE vector. GAPDH served as a loading control. **(I)** Western blot analysis of ITGB1 and PPARγ expression in rhPOSTN-treated (+) or untreated (−) cells transfected with siNC or siITGB1. **(J)** Fluorescence imaging of intracellular lipid oxidation (BODIPY) and oxidative status (DCFH-DA) in POSTN-treated GC cells following ITGB1 knockdown. **(K)** Expression of lipid metabolism-related genes and immunosuppressive phenotype-associated genes following PPARγ rescue in ITGB1-knockdown cells. **(L)** Western blot analysis showing PI3K/AKT/mTOR signaling following PPARγ rescue in ITGB1-knockdown cells. **(M)** Western blot analysis of PPARγ expression following treatment with the PI3K inhibitor Alpelisib, the AKT inhibitor Capivasertib, or the mTOR inhibitor Everolimus under POSTN stimulation. **(N)** Western blot analysis of NRF2, NQO1, and GPX4 expression in control and POSTN-overexpressing cells following treatment with N-acetyl-L-cysteine (NAC) or Ferrostatin-1.

Given the pronounced colocalization of POSTN^+^ CAFs and lipid-stressed macrophages, we next sought to delineate the molecular mechanisms underlying their interaction. Prior studies have established integrins as key receptors mediating POSTN signaling [[33], [34], [35]]. To systematically evaluate their involvement in lipid metabolism, we performed a high-throughput siRNA library screen targeting 19 integrin subunits with relatively high expression in GC. Because integrin signaling is a well-established driver of epithelial–mesenchymal transition (EMT), and N-cadherin upregulation is a hallmark of EMT [34], we used N-cadherin as a functional readout. A reduction in N-cadherin expression following integrin knockdown served as a positive control to validate the screen. Under these conditions, integrin subunits whose silencing led to reduced expression of lipid metabolism–related genes were considered potential mediators. This screen identified ITGB1, ITGAV, ITGAX, ITGB2, and ITGB5 as candidates (Fig. 5A).

To refine these candidates, we integrated single-cell communication analysis, which revealed that POSTN^+^ CAF–epithelial cell interactions were dominated by ITGA3–ITGB1, whereas POSTN^+^ CAF–macrophage interactions were primarily mediated by ITGA4–ITGB1, collectively highlighting ITGB1 as the most frequent binding partner (Fig. 5B). Consistent with these predictions, ITGA3 expression was readily detected in YTN16 and AGS cells, while macrophages exhibited ITGA4 expression (Fig. S15a), supporting the biological plausibility of these interaction patterns. Control experiments confirmed that individual knockdown of ITGB1, ITGA3, or ITGA4 did not induce significant apoptosis or impair cell viability (Fig. S15b–d).

Direct binding between POSTN and ITGB1 was confirmed both in tumor cells (Fig. 5C) and macrophages, where His-tagged rhPOSTN successfully pulled down ITGB1 (Fig. 5D). Moreover, rhPOSTN treatment increased ITGB1 expression at the RNA level, particularly in M2 macrophages (Fig. 5E). Protein–protein docking further supported a direct interaction interface between POSTN and ITGB1, identifying several putative amino acid contacts (Fig. 5F).

Given our previous enrichment analyses showing recurrent activation of PI3K–AKT–mTOR signaling in POSTN-exposed cells, we next examined whether ITGB1 mediates this pathway. Indeed, POSTN overexpression increased the phosphorylation levels of PI3K p85, AKT, and mTOR (Fig. 5G). In parallel, POSTN markedly upregulated PPARγ protein expression, whereas ITGB1 knockdown largely abolished this induction (Fig. 5H and I), indicating that ITGB1 functions upstream of PPARγ upregulation. Functionally, ITGB1 depletion significantly attenuated POSTN-induced tumor cell proliferation (Fig. S15e), reduced intracellular ROS accumulation, and diminished lipid oxidation, effects that could be restored by rhPOSTN supplementation (Fig. 5J, S15f). To further establish the specificity of this mechanism, we generated a siRNA-resistant ITGB1 expression construct containing silent mutations within the siRNA-targeting sequence (Fig. S15g). Re-expression of siRNA-resistant ITGB1 effectively restored intracellular lipid droplet accumulation, as demonstrated by BODIPY staining (Fig. S15h), and rescued the elevated lipid oxidation state induced by POSTN stimulation (Fig. S15i). In parallel, ITGB1 silencing also substantially reduced M2-associated macrophage polarization (Fig. S15j). Collectively, these findings indicate that ITGB1 is not merely associated with, but is functionally required for, POSTN-driven metabolic and immunological reprogramming.

To further determine whether PPARγ functions downstream of ITGB1, we performed rescue experiments by ectopically expressing PPARγ following ITGB1 knockdown. Restoration of PPARγ expression partially rescued the suppression of lipid metabolism-related genes and immunosuppressive phenotype-associated genes caused by ITGB1 depletion (Fig. 5K). Importantly, PPARγ re-expression did not restore the reduced p-PI3K/AKT/mTOR signaling activity induced by ITGB1 knockdown (Fig. 5L), supporting the hierarchical positioning of PPARγ downstream of the ITGB1–PI3K/AKT/mTOR axis. Next, cells were treated with the PI3K inhibitor Alpelisib, the AKT inhibitor Capivasertib, or the mTOR inhibitor Everolimus. Pharmacological inhibition of each signaling node markedly suppressed POSTN-induced PPARγ upregulation (Fig. 5M). In particular, PI3K inhibition with Alpelisib also attenuated lipid oxidation, ROS accumulation, and M2-associated macrophage polarization (Fig. S15k–m), phenocopying the effects of ITGB1 depletion. Importantly, ectopic re-expression of PPARγ partially restored the lipid oxidation phenotype suppressed by Alpelisib (Fig. S15n), providing additional evidence that PPARγ functions downstream of PI3K/AKT/mTOR.

Finally, to determine whether lipid peroxidation functionally contributes to the observed redox and immunological phenotypes, cells were treated with the antioxidant N-acetyl-L-cysteine (NAC) or the lipid peroxidation inhibitor Ferrostatin-1. Both interventions substantially reduced the expression of NRF2, NQO1, and GPX4 (Fig. 5N), partially reversed M2-associated macrophage polarization (Fig. S15o), and attenuated lipid oxidation, oxidative-state signals, and mitochondrial dysfunction (Fig. S15p–r). These findings support a model in which POSTN-induced lipid peroxidation acts as a critical downstream effector linking metabolic rewiring to immune suppression.

Together, these data delineate a mechanistic cascade whereby POSTN directly engages ITGB1, activates PI3K/AKT/mTOR-dependent PPARγ signaling, and drives lipid metabolic reprogramming, oxidative stress, and macrophage immunosuppressive polarization, thereby coupling stromal signaling with immune evasion within the TME.

### 3.6 POSTN as a prognostic biomarker and mediator of immunotherapy non-response

**Fig. 6:**
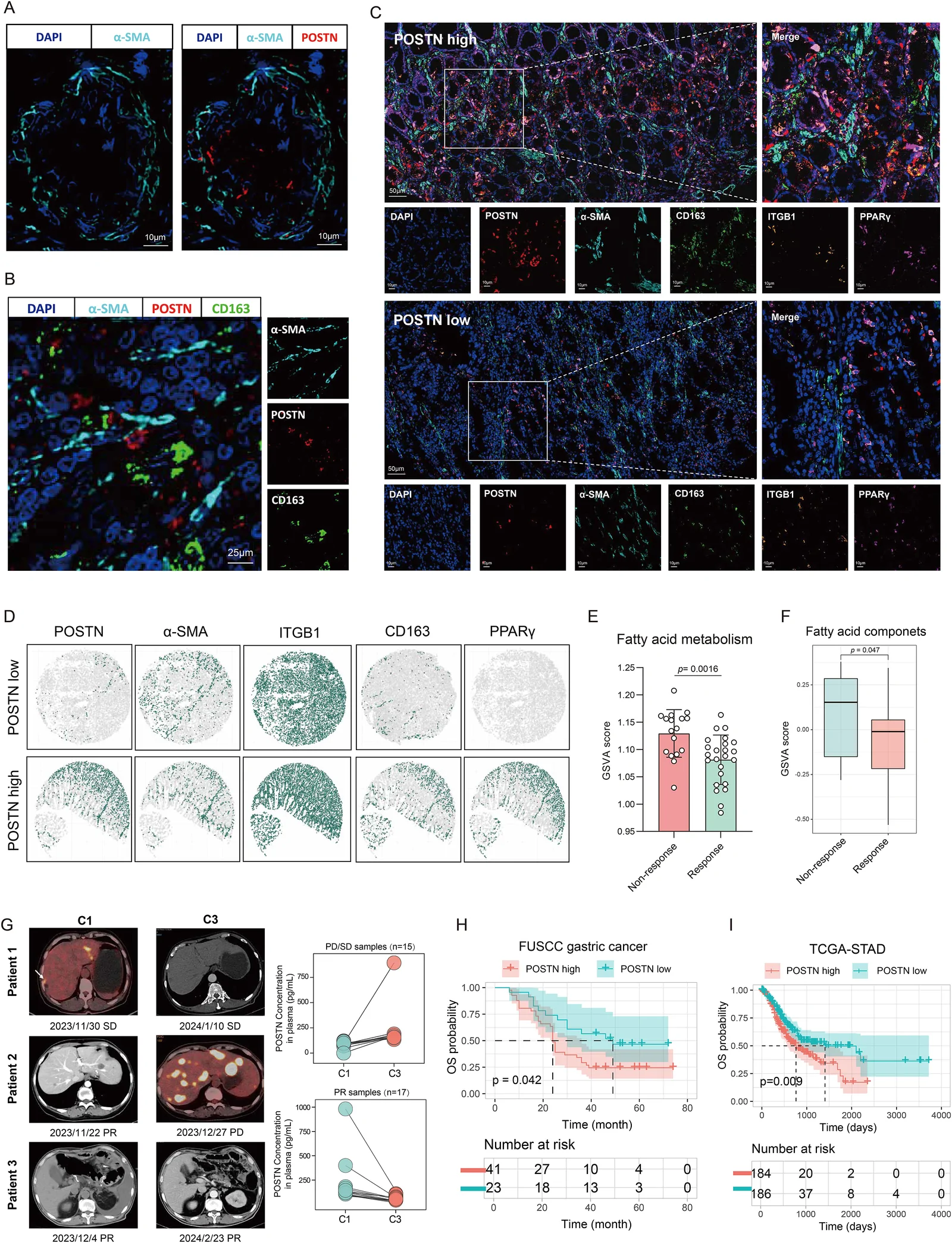
**Clinical validation of POSTN as a prognostic and immunomodulatory factor in GC. (A)** Immunofluorescence (IF) images of gastric cancer tissues stained for DAPI (blue), α-SMA (cyan), and POSTN (red). Left: dual-channel images; right: merged triple-channel images. **(B)** IF images of gastric tissues stained for DAPI (blue), α-SMA (cyan), POSTN (red), and CD163 (green). Left: merged image; right: single-channel views. **(C)** Multiplex IF images of gastric tissues with high (top) and low (bottom) POSTN expression, stained for DAPI (blue), POSTN (red), α-SMA (cyan), CD163 (green), ITGB1 (orange), and PPARγ (purple). **(D)** Representative images of tissue sections with low and high POSTN expression, stained for POSTN, α-SMA, ITGB1, CD163, and PPARG (color intensity indicates expression level). **(E)** Bar plot comparing Hallmark term “fatty acid metabolism” scores between groups. **(F)** Box plot showing differences in fatty acid component scores. **(G)** Computed tomography (CT) images of three GC patients at different treatment time points (left), showing responses (SD, stable disease; PR, partial response; PD, progressive disease). Scatter plots of plasma POSTN levels at Cycle 1 and Cycle 3 treatment in patients with PD/SD (n = 15) versus PR (n = 17) (right). **(H)** Kaplan–Meier survival analysis of overall survival in the FUSCC non-surgical GC cohort, stratified by best cut-off POSTN expression (P = 0.042). Numbers at risk are shown below. **(I)** Kaplan–Meier survival analysis of overall survival in the TCGA-STAD cohort, stratified by POSTN expression (P = 0.009). Numbers at risk are shown below.

Having established the tumor-promoting effects of POSTN in vitro *and* in vivo, we turned to clinical cohorts to evaluate whether POSTN expression is associated with prognosis and response to immunotherapy. To further investigate POSTN distribution in the TME, we performed mIF staining on the FUSCC mIF tissue microarray cohort comprising 180 GC patients. This analysis revealed that CAFs encircled tumor cells and exhibited scattered POSTN expression (Fig. 6A). Notably, POSTN^+^ CAFs were frequently colocalized with CD163^+^ macrophages, implicating their role in recruiting M2 macrophages into the tumor milieu (Fig. 6B). Moreover, tumor regions with higher POSTN abundance exhibited denser M2 macrophage infiltration and increased PPARγ expression (Fig. 6C–D, S16a). Quantitative analyses reinforced these observations: POSTN fluorescence intensity across the whole cohort positively correlated with CD163 (Fig. S16b–c), with significantly higher CD163 signal in POSTN-high regions than in POSTN-low counterparts (Fig. S16d), and this correlation was stronger when specifically examining POSTN^+^ CAFs and CD163^+^ macrophages (Fig. S16e). In parallel, transcriptomic profiling of macrophages under different polarization states revealed that PPARγ expression was substantially higher in M2 macrophages than in M1 macrophages (Fig. S16f), further supporting the association between PPARγ signaling and the immunosuppressive macrophage phenotype observed in POSTN-rich tumors (Fig. S16g). Clinically, POSTN positivity was more frequent in patients with advanced T and overall stage, though no association was observed with N stage (Fig. S16h–j). Enrichment analyses of differentially expressed genes (DEGs) using KEGG, Hallmark, WikiPathways, and Reactome databases consistently highlighted fatty acid metabolism pathways in non-responders, indicating elevated lipid metabolic activity in this group (Fig. S16k). Subsequent analysis of all Hallmark pathways in FUSCC patients receiving immunotherapy revealed markedly reduced lipid metabolism activity in responders compared with non-responders (Fig. S16l), a finding further validated by quantitative comparisons (Fig. 6E). Building on this observation, we systematically evaluated lipid metabolite levels across all patients and found a significant increase in non-responders (Fig. 6F). In FUSCC immunotherapy cohort consisting of 32 paired plasma samples collected longitudinally during immunotherapy, plasma POSTN levels remained stable or increased in individuals with progressive disease (PD) or stable disease (SD), whereas a clear decline was observed in patients achieving partial response (PR) (Fig. 6G). Consistently, both the FUSCC cohort and TCGA-STAD dataset demonstrated that high POSTN expression was associated with significantly poorer prognosis (Fig. 6H and I). These findings collectively indicate that POSTN functions as a key driver of immunosuppressive remodeling, correlates with enhanced lipid metabolism, and serves as a prognostic biomarker in GC clinical cohorts.

### 3.7 LNP-siPostn attenuates lipid metabolic activity and suppresses tumor progression

**Fig. 7:**
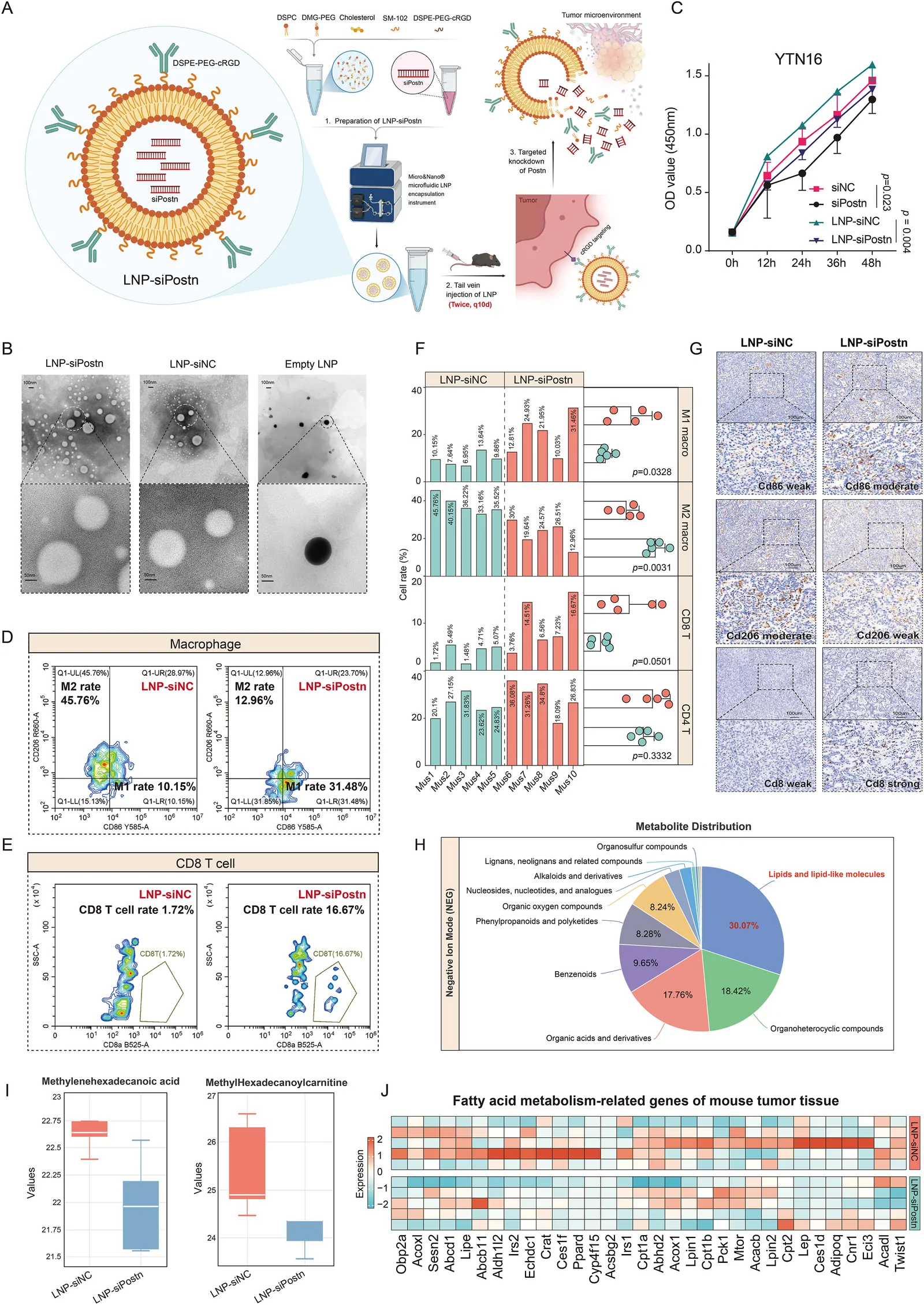
**Therapeutic targeting of POSTN with LNP-siPostn attenuates tumor growth and remodels the immune microenvironment. (A)** Schematic diagram of LNP-siPostn formulation and application, showing LNP composition (DSPC, DMG-PEG, Cholesterol, SM-102, DSPE-PEG-cRGD), preparation process, tumor-targeted delivery, and uptake by tumor cells. **(B)** Transmission electron microscopy images of LNP-siPostn, LNP-siNC, and empty LNP showing particle morphology. **(C)** Cell proliferation curves of YTN16 GC cells treated with siNC, LNP-siNC, or LNP-siPostn over 48 h, measured by CCK-8 assay. **(D)** Representative flow cytometry plots of M1 (CD86^+^) and M2 (CD206^+^) macrophage frequencies. **(E)** Representative flow cytometry plots of CD8^+^ T cells. **(F)** Flow cytometry quantification of immune cell subsets, including M1 macrophages (CD86^+^), M2 macrophages (CD206^+^), CD4^+^ T cells, and CD8^+^ T cells, in tumors treated with LNP-siNC or LNP-siPostn (n = 5 mice per group). **(G)** Immunohistochemistry images of GC tissues grouped by expression of macrophage and T cell markers (Cd206, Cd86, and Cd8). **(H)** Pie charts of metabolite distribution detected in negative ion mode (NEG), classified by compound type. **(I)** Box plots of representative lipid metabolites (Methylenehexadecanoic acid, Methylhexadecanoylcarnitine), showing significant abundance differences between LNP-siNC and LNP-siPostn groups. **(J)** Heatmap of gene expression related to lipid metabolism, with color intensity representing normalized expression levels (red: high; blue: low) in LNP-siNC and LNP-siPostn groups. Unless otherwise indicated, data are presented as mean ± SD from three independent biological replicates (n = 3).

Identifying effective therapeutic targets is essential for advancing personalized treatment in precision medicine. Given the predictive efficacy of POSTN in treatment response, we sought to determine whether targeting the POSTN axis could enhance cancer therapy outcomes. To this end, we designed an LNP-siRNA delivery system for in vivo knockdown of Postn in GC murine models (Fig. 7A). Three interference sequences targeting Postn were constructed, among which siPostn#3 exhibited the most efficient knockdown (Fig. S17a).

Transmission electron microscopy revealed that the formulated nanoparticles exhibited a uniform spherical morphology (Fig. 7B). Dynamic light scattering analysis further demonstrated a narrow size distribution with an average hydrodynamic diameter of approximately 82.30 nm for LNP-siNC and 86.87 nm for LNP-siPostn (Fig. S17b), while zeta-potential measurements showed a moderately negative surface charge (approximately −7.093 mV for LNP-siNC and −6.114 mV for LNP-siPostn), indicative of favorable colloidal stability (Fig. S17c). In addition, the nanoparticles displayed a low polydispersity index (PDI), suggesting homogeneous particle formation and good formulation reproducibility (0.1505 for LNP-siNC and 0.1393 for LNP-siPostn). More than 98% of siRNA was successfully incorporated into the LNP formulation. Consistent with these physicochemical properties, serum incubation assays demonstrated that LNP encapsulation effectively protected siRNA from degradation and maintained formulation stability during in vitro serum incubation (Fig. S17d). We next evaluated the biological activity of the formulation. LNP-mediated delivery efficiently reduced Postn expression (Fig. S17e) and markedly suppressed tumor cell proliferation in vitro (Fig. 7C), achieving an inhibitory effect comparable to that of conventional transfection-based siRNA delivery.

After ensuring comparable baseline tumor sizes between groups (Fig. S18a–b), LNP-siPostn was administered via tail-vein injection and slowed tumor growth compared with controls (Fig. S18c–d). Subsequent analyses confirmed successful Postn silencing within xenograft tissues (Fig. S18e). Notably, modulation of the TME was observed, with an increased proportion of M1 macrophages, decreased M2 macrophages, and elevated CD8^+^ T cells, while CD4^+^ T cell frequencies remained unchanged (Fig. 7D–F, S18f). These findings were further validated by immunohistochemistry (Fig. 7G). To further characterize the macrophage phenotypic changes induced by POSTN silencing, we found a coordinated increase in M1-related genes accompanied by suppression of M2-associated signatures (Fig. S18g). Consistent with our proposed mechanism linking POSTN to metabolic reprogramming, LNP-siPostn treatment also reduced the expression of multiple lipid metabolism–related genes within the tumor microenvironment (Fig. S18h). Together, these findings suggest that therapeutic POSTN inhibition not only restrains tumor growth but also alleviates immunosuppressive and metabolic remodeling within the TME.

To investigate the metabolic alterations induced by Postn silencing in GC, we conducted untargeted metabolomics on samples from the LNP-siNC and LNP-siPostn groups. Principal component analysis revealed distinct global metabolic profiles upon Postn knockdown (Fig. S19a). Metabolite distribution by compound class in negative (NEG) and positive (POS) ion modes showed lipids and lipid-like molecules as the most abundant category (Fig. 7H, S19b). To explore the functional relevance of these changes, KEGG pathway classification indicated enrichment across multiple pathways, with lipid metabolism containing the largest number of metabolites, underscoring the central involvement of lipid metabolic processes (Fig. S19c). At the metabolite level, several representative lipids, such as Methylhexadecanoic acid, Methylhexadecanoylcarnitine, 10Z-Heptadeca-6E-enoylcarnitine, Stearylcarnitine, Isoveratrylglycolic acid, and 15-Deoxy-Δ12,14-Prostaglandin J2, showed significant abundance differences between groups (Fig. 7I, S19d). We identified 123 upregulated and 51 downregulated metabolites in the LNP-siPostn group compared with LNP-siNC (Fig. S19e). Heatmaps further highlighted distinct clustering patterns of metabolite intensities between groups in both NEG and POS modes (Fig. S19f). From a transcriptomic perspective, RNA-seq analysis revealed reduced expression of lipid metabolism-associated genes (Fig. 7J), along with downregulation of lipid metabolism–related pathways, including arachidonic acid metabolism (ES = −1.5891, NP = 0.0071) and alpha linolenic acid metabolism (ES = −1.7376, NP = 0.0033) (Fig. S19g).

Together, these findings demonstrate that therapeutic POSTN silencing suppresses tumor progression and alleviates immunosuppressive metabolic remodeling within the TME. Integrated multi-omics analyses consistently identified attenuation of lipid metabolic activity as a major consequence of Postn ablation. These observations prompted us to further examine whether POSTN inhibition could enhance the efficacy of PD-1 blockade in vivo.

### 3.8 LNP-siPostn enhances anti-PD-1 therapy by remodeling the immunosuppressive TME

**Fig. 8:**
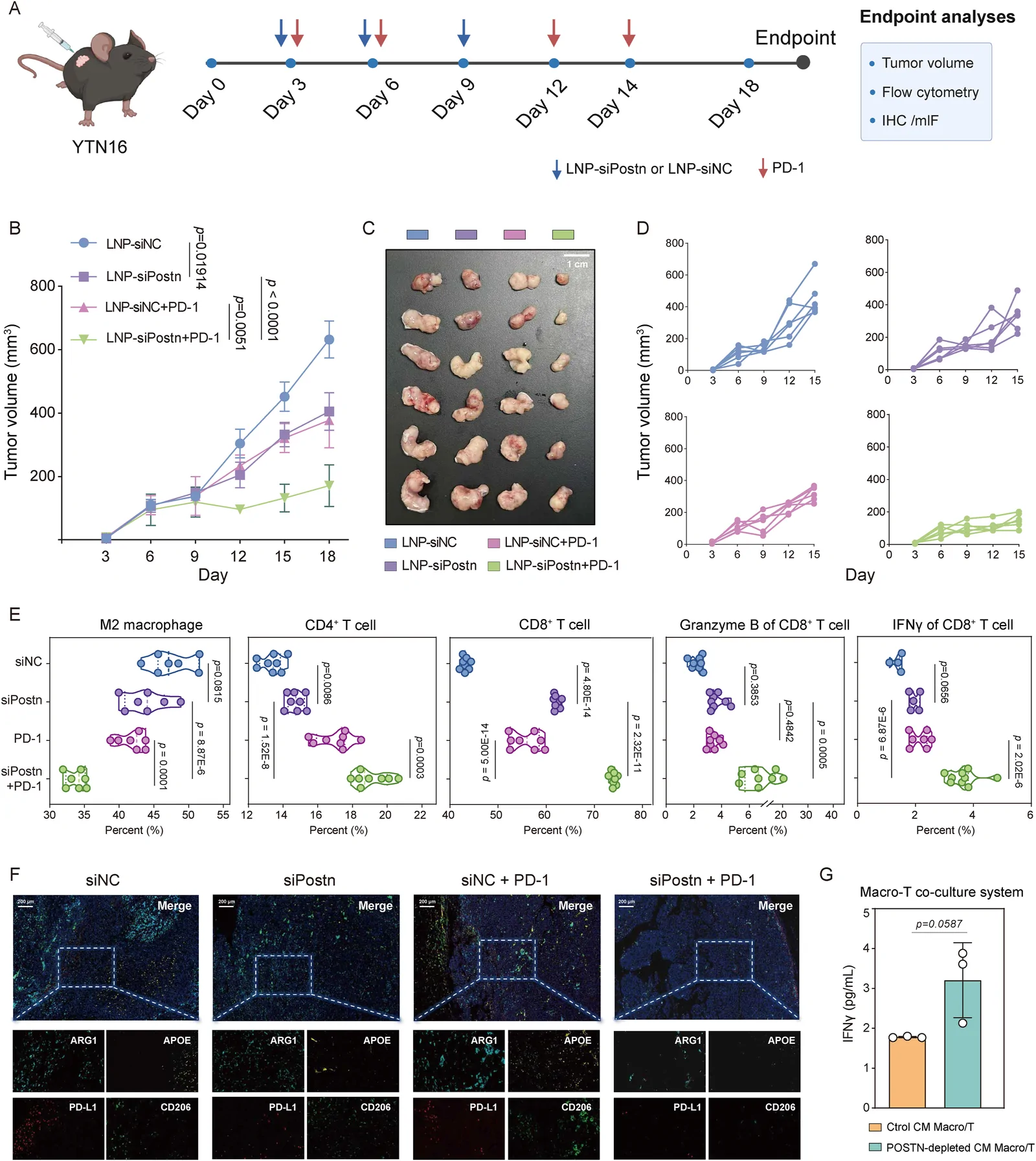
**LNP-siPostn enhances anti-PD-1 therapy by reversing macrophage-mediated immunosuppression and remodeling the tumor microenvironment. (A)** Schematic illustration of the in vivo therapeutic study design. C57BL/6 mice bearing subcutaneous YTN16 tumors were randomized when tumors reached approximately 50 mm^3^ and treated with LNP-siNC, LNP-siPostn, anti-PD-1 plus LNP-siNC, or anti-PD-1 plus LNP-siPostn. LNPs were administered intravenously every 3 days for three total doses, and anti-PD-1 antibody was administered intraperitoneally twice weekly for 2 weeks. Tumor growth was monitored throughout treatment, and tumors were harvested for downstream analyses. **(B-D)** Individual tumor growth trajectories for mice in each treatment group throughout the experimental period (n = 6 mice per group). **(E)** Flow cytometric quantification of tumor-infiltrating CD206^+^ M2-like macrophages, CD4^+^ T cells, CD8^+^ T cells, and Granzyme B^+^ and IFN-γ^+^ immune cell populations in tumors from the indicated treatment groups (n = 6 mice per group). Both siRNA treatments were delivered via LNP formulation. **(F)** Representative mIF staining of macrophage-associated immunosuppressive markers, including APOE, PD-L1, ARG1, and CD206, in tumor tissues following the indicated treatments. **(G)** Quantification of IFN-γ levels in the supernatants of macrophage–T-cell co-culture assays. Unless otherwise indicated, data are presented as mean ± SD from three independent biological replicates (n = 3).

We next evaluated whether POSTN silencing could enhance the therapeutic efficacy of PD-1 blockade in vivo (Fig. 8A). Notably, LNP-siPostn treatment was well tolerated, with no significant changes in body weight (Fig. S20a), hematological parameters, or serum biochemical indices compared with controls (Tables S8 and 9). Furthermore, no detectable immune toxicity was observed following repeated administration (Fig. S20b–c). These results establish LNP-siPostn as a safe and effective platform for therapeutic targeting of POSTN in vivo. While both LNP-siPostn and anti-PD-1 monotherapy moderately suppressed tumor growth, the combination treatment produced the most pronounced antitumor effect, resulting in significantly reduced tumor volume compared with either treatment alone (Fig. 8B–D).

To determine whether this enhanced therapeutic response was associated with immune remodeling, we characterized tumor-infiltrating immune populations following treatment. Notably, combined LNP-siPostn and anti-PD-1 therapy markedly reduced the proportion of CD206^+^ M2-like macrophages, while increasing the infiltration of CD4^+^ and CD8^+^ T cells. Moreover, the abundance of Granzyme B and IFN-γ was significantly elevated in tumors receiving combination treatment (Fig. 8E, S20d, S21a-e), indicating enhanced cytotoxic T-cell activation and effector function within the TME.

Because changes in macrophage abundance do not necessarily reflect alterations in their functional state, we further assessed macrophage-associated immunosuppressive markers. Combination treatment substantially decreased the expression of APOE, PD-L1, ARG1, and CD206 (Fig. 8F). These findings suggest that POSTN blockade primarily reprograms tumor-associated macrophages from an immunosuppressive phenotype rather than simply depleting macrophage populations, thereby alleviating macrophage-mediated immune suppression. To further validate the functional consequences of POSTN-induced macrophage polarization, we performed macrophage–T-cell co-culture assays in vitro. Macrophages were first cultured with conditioned medium from POSTN-overexpressing fibroblasts and then co-cultured with T cells. T cells exposed to these POSTN-educated macrophages exhibited reduced IFN-γ production compared with those co-cultured with control macrophages (Fig. 8G), indicating diminished effector activity. Collectively, these results indicate that targeting POSTN not only enhances the antitumor efficacy of PD-1 blockade but also restores antitumor immunity through reversal of macrophage-mediated immunosuppression.

## 4 Discussion

CAFs are central components of the TME, actively shaping immune cell infiltration and metabolic landscapes [35]. Traditionally, CAFs have been viewed mainly as structural components that remodel the extracellular matrix and secrete growth factors [36]. Beyond their established structural and immunomodulatory roles, our study identifies POSTN^+^ myCAFs as a previously unrecognized stromal population that promotes lipid metabolic reprogramming in both tumor cells and macrophages. Mechanistically, POSTN activates an ITGB1-dependent PI3K/AKT/mTOR–PPARγ signaling axis, driving lipid-stressed macrophage polarization and an immunosuppressive TME. These findings expand the role of CAFs from structural supporters to metabolic regulators of the gastric cancer immune microenvironment.

Previous studies have established POSTN as a CAF-associated matricellular protein that promotes extracellular matrix remodeling, tumor progression, and macrophage recruitment or M2-like polarization in various cancers [35]. POSTN is a key matricellular protein with well-documented roles in facilitating extracellular matrix remodeling, promoting cancer cell invasion and metastasis, and contributing to therapy resistance across diverse cancer types [33,[37], [38], [39]]. Consequently, POSTN overexpression is consistently associated with an immunosuppressive milieu and poor patient prognosis [40]. Although the immunosuppressive role of CAFs is recognized, how the POSTN^+^ myCAF subset drives immunosuppression through lipid metabolic reprogramming remains unclear. Our work further mechanistically links POSTN secretion to lipid metabolic remodeling and macrophage polarization. Here, we reveal that POSTN secretion remodels lipid metabolism in macrophages, inducing lipid droplet accumulation and oxidative stress, thereby promoting an immunosuppressive M2-like polarization. This study establishes a novel stromal-immune metabolic axis driven by POSTN, moving beyond the tumor-intrinsic view of lipid regulation and offering new insights into overcoming immunotherapy non-response.

Lipid metabolism has emerged as a critical determinant of immune cell function within the TME [41]. Prior studies have shown that excess fatty acid uptake and accumulation of lipid peroxidation products, such as 15-deoxy-Δ12,14-prostaglandin J2 and long-chain acylcarnitines, can suppress T-cell proliferation, enhance ROS generation, and skew macrophages toward an M2-like phenotype, collectively fostering an immunosuppressive environment [42,43]. Our data add a new dimension by demonstrating that CAF-derived POSTN acts as an important functional contributor to this process, inducing lipid droplet accumulation and oxidative stress specifically in macrophages, thereby promoting a lipid-stressed M2 state. This finding expands the conventional view that lipid dysregulation is primarily tumor-intrinsic, emphasizing instead the importance of stromal–immune metabolic crosstalk in determining immunotherapy outcomes.

Our study establishes a mechanistic axis in which POSTN secreted by CAFs engages ITGB1 to activate PI3K/AKT/mTOR signaling, culminating in PPARγ upregulation and lipid metabolic reprogramming. Integrins have been widely implicated as canonical receptors for POSTN, with previous studies primarily focusing on the αvβ3 and αvβ5 integrin complexes, in processes such as extracellular matrix remodeling, angiogenesis, and epithelial–mesenchymal transition [[44], [45], [46]]. By combining high-throughput siRNA screening, single-cell communication analysis, and biochemical validation, we identified ITGB1 as the dominant receptor mediating POSTN signaling in both tumor cells and macrophages. Importantly, we demonstrate that the POSTN–ITGB1 interaction is not merely structural but functionally couples to the PI3K/AKT/mTOR cascade, a pathway classically known for integrating growth factor and nutrient signals. This activation converges on PPARγ, a master regulator of lipid metabolism, thereby driving lipid droplet accumulation and enforcing an immunosuppressive macrophage phenotype. Previous studies have highlighted PI3K/AKT/mTOR as a central axis in tumor-intrinsic metabolic adaptation [47,48], whereas our work demonstrates that stromal–immune crosstalk can also hijack this pathway, redirecting it toward immunosuppressive lipid metabolism. The identification of a POSTN-ITGB1-PI3K/AKT/mTOR-PPARγ axis therefore provides a unifying framework to explain how CAF-derived cues intersect with canonical metabolic signaling networks to shape the TME. Importantly, beyond mechanistic novelty, our demonstration that LNP-mediated Postn silencing reverses these effects underscores the therapeutic potential of targeting POSTN. By disrupting this stromal-immune metabolic circuit, it may be possible to remodel the TME toward a more permissive state for immunotherapy.

Importantly, LNP-mediated Postn silencing enhanced the therapeutic efficacy of PD-1 blockade and was accompanied by increased T-cell infiltration, elevated cytotoxic activity, and reduced immunosuppressive macrophage phenotypes within the TME. Together with our macrophage-T-cell co-culture findings, these observations support an association between POSTN inhibition, immune remodeling, and improved response to PD-1 blockade. However, because CD8^+^ T-cell depletion experiments were not performed, the extent to which the antitumor effects are directly dependent on adaptive immunity remains to be determined. Nevertheless, our findings suggest that targeting POSTN represents a promising strategy to remodel the tumor immune microenvironment and enhance the efficacy of PD-1-based immunotherapy in GC.

Several limitations should also be acknowledged. Although our spatial transcriptomic, single-cell, and co-culture analyses consistently identified CAFs as the predominant source of POSTN within the GC microenvironment, POSTN can also be expressed by tumor cells and other stromal compartments. Moreover, the systemic LNP-siPostn strategy used in this study does not selectively target CAFs and therefore likely suppresses POSTN expression across multiple cellular populations. Consequently, while our data strongly support a major contribution of CAF-derived POSTN to metabolic and immunological remodeling, the relative contributions of CAF- versus tumor cell-derived POSTN could not be fully distinguished in the current experimental setting. Future studies employing cell type-specific genetic manipulation or targeted delivery approaches will be required to precisely define the contribution of individual cellular sources of POSTN.

## 5 Conclusion

In conclusion, this study identifies POSTN^+^ CAFs as critical stromal drivers of lipid metabolism in GC. Mechanistically, POSTN engages ITGB1 to activate the PI3K/AKT/mTOR–PPARγ signaling axis, leading to lipid metabolic alterations, macrophage immunosuppression, and non-response to immunotherapy. By revealing this previously unrecognized stromal–metabolic pathway, our findings link fibroblast heterogeneity to tumor lipid metabolism and immune evasion, and highlight POSTN as a potential therapeutic target in GC.

## CRediT authorship contribution statement

**Qin Ding:** Conceptualization, Formal analysis, Methodology, Writing – original draft, Writing – review & editing. **Ziteng Li:** Resources. **Ruiwen Liu:** Software. **Wanjing Feng:** Validation. **Jiayi Wei:** Methodology. **Chengye Liu:** Validation. **Masami Yamamoto:** Resources. **Tetsuya Tsukamoto:** Resources. **Sachiyo Nomura:** Resources. **Shenglin Huang:** Funding acquisition, Project administration, Supervision. **Xiaodong Zhu:** Funding acquisition, Project administration, Supervision.

## Ethics approval and consent to participate

This study was approved by the Ethics Committee of Fudan University Shanghai Cancer Center (No. 2402291-19). All human participants provided informed consent before enrollment. All animal experiments were conducted in accordance with the protocol approved by the Institutional Animal Care and Use Committee (IACUC) of Fudan University. The maximal tumor burden permitted was 2000 mm^3^, and this limit was not exceeded in any animal.

## Consent for publication

Not applicable.

## Availability of data and materials

All data generated or analyzed during this study are included in this article and its Supplementary Information files. The single-cell RNA sequencing dataset used in this study is provided in Table S2. Spatial transcriptomic data were obtained from the Gene Expression Omnibus (GEO) under accession number GSE251950. Publicly available bulk transcriptomic datasets, including TCGA-STAD and PRJEB25780, were obtained from the corresponding repositories as described in the Methods section.

## Declaration of competing interest

The authors declare that they have no known competing financial interests or personal relationships that could have appeared to influence the work reported in this paper.

## Abbreviations

AUC: area under the curve
CAFs: cancer-associated fibroblasts
DEGs: differentially expressed genes
EMT: epithelial-mesenchymal transition
ES: enrichment score
ESTIMATE: Estimation of STromal and Immune cells in MAlignant Tumors using Expression data
FUSCC: Fudan University Shanghai Cancer Center
GC: gastric cancer
ICB: immune checkpoint blockade
iCAFs: inflammatory cancer-associated fibroblasts
KEGG: Kyoto Encyclopedia of Genes and Genomes
LC-MS/MS: liquid chromatography-tandem mass spectrometry
LNP: lipid nanoparticle
LNP-siPostn: lipid nanoparticle encapsulated siRNA targeting Postn
mIF: multiplex immunofluorescence
MNU: N-methyl-N-nitrosourea
myCAFs: myofibroblastic cancer-associated fibroblasts
NAC: N-acetyl-L-cysteine
NEG: negative ion mode
OS: overall survival
PBMCs: peripheral blood mononuclear cells
PD: progressive disease
PFS: progression-free survival
POS: positive ion mode
PR: partial response
QC: quality control
RECIST: Response Evaluation Criteria in Solid Tumors
rhPOSTN: recombinant human POSTN
ROS: reactive oxygen species
RT-qPCR: reverse transcription quantitative polymerase chain reaction
scRNA-seq: single-cell RNA-sequencing
SD: stable disease
siRNA: small interfering RNA
siNC: siRNA negative control
ST: spatial transcriptomics
STAD: stomach adenocarcinoma
TAMs: immunosuppressive tumor-associated macrophages
TCGA: The Cancer Genome Atlas
TIDE: Tumor Immune Dysfunction and Exclusion
TMAs: tissue microarrays
TME: tumor microenvironment
