# Supplementary material for: Cancer-associated fibroblast-derived POSTN as a metabolic “switch” shapes lipid-stressed macrophage polarization in gastric cancer
Source: Redox Biol. 2026 Sep 9;97:104389. doi: 10.1016/j.redox.2026.104389 (PMC13587811; doi:10.1016/j.redox.2026.104389)
Supplement: Supplementary file 1 [file mmc1.pdf]

Supplementary Materials for

Cancer-associated fibroblast-derived POSTN as a metabolic "switch" shapes lipid-stressed macrophage polarization in gastric cancer

Qin Ding[1,2], Ziteng Li[1,2], Ruiwen Liu[1,2], Wanjing Feng[1,2], Jiayi Wei[1,2], Chenye Liu[1,2], Masami Yamamoto[3], Tetsuya Tsukamoto[4], Sachiyo Nomura[5], Shenglin Huang[1,2,6*], Xiaodong Zhu[1,2*]

[1] Department of Medical Oncology, Fudan University Shanghai Cancer Center, Shanghai Key Laboratory of Medical Epigenetics, Institutes of Biomedical Sciences, Fudan University, Shanghai 200032, China.

[2] Department of Oncology, Shanghai Medical College, Fudan University, Shanghai 200032, China.

[3] Laboratory of Physiological Pathology, School of Veterinary Nursing and Technology, Nippon Veterinary and Life Science University, Tokyo, Japan.

[4] Department of Diagnostic Pathology, Fujita Health University School of Medicine, Toyoake, Aichi, Japan.

[5] Department of Gastrointestinal Surgery, Graduate School of Medicine, The University of Tokyo, Tokyo, Japan.

[6] State Key Laboratory of Genetics and Development of Complex Phenotypes, Fudan University, Shanghai, China.

***correspondence:**

Shenglin Huang

Email: slhuang@fudan.edu.cn

Xiaodong Zhu

Email: zhuxd@fudan.edu.cn

Contents for supplementary material

Supplementary Experimental Procedures

Supplementary Figure S1. (related to Fig. 1)

Supplementary Figure S2. (related to Fig. 1)

Supplementary Figure S3. (related to Fig. 1)

Supplementary Figure S4. (related to Fig. 1)

Supplementary Figure S5. (related to Fig. 1)

Supplementary Figure S6. (related to Fig. 2)

Supplementary Figure S7. (related to Fig. 2)

Supplementary Figure S8. (related to Fig. 2)

Supplementary Figure S9. (related to Fig. 3)

Supplementary Figure S10. (related to Fig. 3)

Supplementary Figure S11. (related to Fig. 4)

Supplementary Figure S12. (related to Fig. 4)

Supplementary Figure S13. (related to Fig. 4)

Supplementary Figure S14. (related to Fig. 4)

Supplementary Figure S15. (related to Fig. 5)

Supplementary Figure S16. (related to Fig. 6)

Supplementary Figure S17. (related to Fig. 7)

Supplementary Figure S18. (related to Fig. 7)

Supplementary Figure S19. (related to Fig. 7)

Supplementary Figure S20. (related to Fig. 8)

Supplementary Figure S21. (related to Fig. 8)

Supplementary Table S1. Baseline Characteristics of Patients with Tumor Biopsy Tissue Sources.

Supplementary Table S2. The single-cell datasets included in this study.

Supplementary Table S3. qPCR primer sequences of macrophage markers and internal reference.

Supplementary Table S4. qPCR primer sequences of fatty acid metobolism-related genes.

Supplementary Table S5. qPCR primer sequences of siRNA library.

Supplementary Table S6. Sequence report of the siRNA library.

Supplementary Table S7. Detailed list of antibodies used in this study.

Supplementary Table S8. Hematological parameters in C57 mice following administration of LNP-siPOSTN.

Supplementary Table S9. Serum biochemical parameters in C57 mice following administration of LNP.

Note. Due to the excessive length of the tables, they are provided as an Excel file titled “Supplementary_tables.xlsx”, with only the title displayed here.

Supplementary Experimental Procedures

Cell culture

HGC27 cells (Ethephon Co. Ltd, Cat# YCL-0123) were maintained in RPMI-1640 medium (Gibco) supplemented with 20% fetal bovine serum (FBS; Gibco) and 1% penicillin–streptomycin (PS; Sigma). AGS cells (Ethephon Co. Ltd, Cat# YCL-0348) were cultured in Ham's F-12K medium (Kaighn's modification; Gibco) with 10% FBS and 1% PS. MKN45 cells (BDBIO Co. Ltd, Cat# C5109) were grown in RPMI-1640 medium (Gibco) containing 10% FBS and 1% PS. The murine gastric cancer cell line YTN16, established through the previously published N-methyl-N-nitrosourea (MNU) carcinogenesis model in $p53^{+/-}$ mice[1], was cultured in DMEM (Corning) containing 0.1% $MITO^+$ Serum Extender (Corning).

THP-1 monocytes (Ethephon Co. Ltd, Cat# YCL-0351) were differentiated into macrophages using 150 nM phorbol 12-myristate 13-acetate (PMA; Sigma) for 24 hours, followed by 24 hours resting in PMA-free RPMI-1640. Macrophage polarization was achieved by incubating the cells with 20 ng/ml IFN-γ (PeproTech) and 10 ng/ml LPS (Sigma) for M1 polarization, or with 20 ng/ml IL-4 (PeproTech) and 20 ng/ml IL-13 (PeproTech) for M2 polarization.

The human fibroblast cell line HSF (BDBIO Co. Ltd, Cat #C6028) was cultured in high-glucose DMEM (Gibco) with 10% FBS (Gibco) and 1% PS (Sigma). The murine fibroblast HCAF cell line, derived from primary murine cancer, was immortalized and cultured in high-glucose DMEM (Gibco) with 10% FBS (Gibco) and 1% PS (Sigma).

The identity of each cell line was confirmed by short tandem repeat (STR) profiling. All cell lines were cultured at 37°C in a humidified atmosphere with 5% CO_2. Cells were replenished with fresh medium every 2 days, and trypsinized when cells reached 90% confluency.

Animal model

Specific pathogen free (SPF), female, 6-8 weeks old C57BL/6 mice were purchased from SBF Biotechnology Co. All mice were housed in a pathogen-free environment at 22-25°C with a relative humidity of 45-60% and a 12-hour light/dark cycle. The maximal tumor burden permitted was 2000 mm^3, and this limit was not exceeded in any animal.

Tumor challenge and treatment

YTN16 cells were resuspended in 0.2 mL of phosphate-buffered saline (PBS) and subcutaneously implanted into the flanks of C57BL/6 mice. 5×10^6 YTN16 cells were injected per mouse. MKN45 human GC cells were mixed with HCAF at a 3:1 ratio for subcutaneous implantation. For the experimental group, HCAFs overexpressing POSTN were used, whereas HCAFs transduced with a negative control vector (POSTN-NC) were used for the control group. Cells were resuspended in serum-free medium and Matrigel (1:5) and injected subcutaneously into the flanks of 4–6-week-old female BALB/c nude mice (1×10^6 MKN45 cells per injection site, total volume 100 μL per mouse). Tumor size was measured with calipers at 3-day intervals, and the volume was calculated using the formula: $(\text{length} \times \text{width}^2)/2$, followed by plotting a growth curve over time. All animal experiments were reviewed and approved by the Animal Care and Use Committee and performed in compliance with institutional regulations.

Recombinant POSTN treatment and rescue experiments

Human recombinant POSTN protein (rhPOSTN, MCE company) was dissolved according to the manufacturer's instructions and used at a final concentration of 500 ng/mL unless otherwise indicated. For rescue experiments, cells were transfected with POSTN-targeting siRNA or cultured in POSTN-depleted conditioned medium, followed by supplementation with rhPOSTN. Cells were subsequently subjected to colony formation assays, lipid oxidation measurements, ROS detection, and macrophage polarization analyses.

Macrophage-T cell co-culture assay

To evaluate the functional consequences of POSTN-induced macrophage polarization on T-cell activity, THP1-derived macrophages were first cultured with conditioned medium collected from control fibroblasts or POSTN-overexpressing fibroblasts for 48 h. After stimulation, macrophages were washed with PBS to remove residual conditioned medium and subsequently co-cultured with activated T cells in a transwell co-culture system.

Human peripheral blood T cells were activated using anti-CD3/CD28 stimulation according to the manufacturer's instructions and added to macrophages at the indicated ratio. After 24h of co-culture, T-cell effector function was assessed by measuring IFN-γ production. IFN-γ levels in culture supernatants were determined using ELISA according to the manufacturer's protocol.

RNA isolation, cDNA synthesis, and RT-qPCR

Total RNA was extracted with TRIzol reagent, and first-strand cDNA was synthesized from 1 μg of RNA using the RT PCR Kit (Promega, Cat# LS2052). Quantitative real-time PCR was carried out with SYBR Green Master Mix (Promega, Cat# LS2062). The amplification program consisted of an initial denaturation at 95°C for 30 s, followed by 40 cycles of 95°C for 5 s and 60°C for 30 s. Each sample was analyzed in triplicate. Relative expression levels were determined using the $2^{-\Delta\Delta CT}$ method.

Transcriptome sequencing

Total RNA was isolated from cultured cells or tumor tissues using TRIzol reagent following the manufacturer's instructions. Polyadenylated mRNA was captured with oligo(dT)-attached magnetic beads, fragmented, and subsequently reverse-transcribed to generate cDNA. The obtained cDNA fragments underwent end repair, 3'-adenylation, and adaptor ligation, followed by PCR enrichment to produce sequencing libraries. Libraries were sequenced on an Illumina high-throughput platform.

Western blotting

Cellular proteins were lysed in RIPA buffer containing protease and phosphatase inhibitors. Equivalent protein quantities were resolved by SDS-PAGE and transferred to PVDF membranes. Membranes were blocked with 5% nonfat milk and incubated overnight at 4 °C with primary antibodies specific to target proteins, followed by incubation with HRP-linked secondary antibodies. Protein bands were detected using enhanced chemiluminescence (ECL) reagents.

Mitochondria, fatty acid uptake, and lipid content assays

Mitochondrial membrane potential was assessed by staining cells with 10 nM MitoTracker Green (Beyotime) for 20 minutes at 37°C. After staining, cells were washed and resuspended in PBS for surface marker staining. Fatty acid uptake was measured by incubating cells in medium containing 0.5 mM BODIPY (Beyotime) at 37°C with 5% CO_2 for 20 minutes. Following incubation, cells were washed and resuspended in PBS. For lipid content detection, cells were stained with

BODIPY 581/591 C11 (Beyotime) at 2μM for 30 minutes at 37°C in the dark. Lipid droplets were visualized by staining fixed cells with 500 ng/mL BODIPY 493/503 (Beyotime) in PBS for 20 minutes at room temperature in the dark. Cells were washed, counterstained with DAPI, and imaged using a confocal microscope (Olympus).

Reactive Oxygen Species (ROS) Detection

Cells were incubated with 10 μM DCFH-DA (diluted in PBS or extracellular solution) at 37°C for 20 min. After three washes with PBS to remove excess probe, cells were treated with the experimental compounds for 20-30 min. Fluorescence intensity was measured using a confocal microscope, or flow cytometer (excitation/emission: 488/525 nm). PBS treated cells served as negative controls.

Lipid Peroxidation Assay

Cells were incubated with 2 μM BODIPY 581/591 C11 (diluted in PBS) at 37°C for 20 min. For positive controls, cells were pretreated with 1× LpoUp (1:1000 dilution) for 4 h prior to staining. After two PBS washes, fluorescence was analyzed using a fluorescence microscope or microplate reader. Red fluorescence (excitation/emission: 581/591 nm) indicated reduced lipid peroxidation, while green fluorescence (488/510 nm) reflected oxidized states. The ratio of green-to-red fluorescence was calculated to quantify lipid peroxidation levels.

Mitochondrial Membrane Potential Assay

Cells were incubated with 1× TMRE (tetramethylrhodamine, ethyl ester), diluted in detection buffer at 37°C for 15-45 min. After two washes with pre-warmed culture medium, fluorescence was measured using a confocal microscope, or flow cytometer. PBS treated cells served as negative controls. A decrease in red fluorescence intensity indicated loss of mitochondrial membrane potential.

Cholesterol quantification assay

Total cholesterol and free cholesterol concentrations were measured using commercial cholesterol assay kits (Beyotime company, Cat# S0211S) following the manufacturer's instructions. Cellular

lysates were collected and analyzed spectrophotometrically. Cholesterol ester content was calculated as the difference between total cholesterol and free cholesterol levels.

Oil Red O staining

Cells were seeded in 6-well plates and cultured under the indicated experimental conditions. After treatment, cells were washed twice with phosphate-buffered saline (PBS) and fixed with 4% paraformaldehyde for 15 min at room temperature. The cells were then rinsed with distilled water and stained with freshly prepared Oil Red O working solution (Beyotime, Cat. No. C0158S, China) for 15 min at room temperature. Excess dye was removed by washing with distilled water until the background was clear. Lipid droplets were visualized and imaged using a light microscope. For quantitative analysis, the retained Oil Red O dye was eluted with 60% isopropanol, and the absorbance was measured at 510 nm using a microplate reader.

Enzyme-linked immunosorbent assay (ELISA)

Peripheral blood was obtained from GC patients treated at our institution. Serum was separated by centrifugation at 1,500 × g for 10 min at 4 °C and stored at -80 °C until use. Serum POSTN concentrations were determined with a human POSTN ELISA kit (YaJi Biological, Cat# YS07454B) according to the manufacturer's protocol. In brief, standards and serum samples were loaded into 96-well plates precoated with anti-POSTN antibodies, followed by incubation with the detection antibody and color development using the substrate reagent. Absorbance was read at 450 nm with a microplate reader, and analyte concentrations were derived based on the corresponding standard curve. All samples were analyzed in duplicate, and mean values were used for further analysis.

Immunohistochemistry (IHC)

Paraffin-embedded mouse tumor tissues were sectioned at 4 µm, deparaffinized in xylene, and rehydrated through graded ethanol to water. Antigen retrieval was performed in citrate buffer (10 mM, pH 6.0) by microwave heating (or in EDTA buffer, pH 9.0, depending on the antibody), followed by endogenous peroxidase blocking with 3% H_2O_2 for 10 min and protein blocking with 5% normal serum for 30 min at room temperature. Sections were incubated with primary

antibodies against Cd86, Cd163, Cd8, Postn, F4/80, and α-SMA (dilutions and clones listed in Supplementary Table S7) overnight at 4 °C. After washing, slides were incubated with appropriate biotinylated or HRP-conjugated secondary antibodies for 30–60 min at room temperature, developed with DAB substrate, and counterstained with hematoxylin. Negative controls omitting the primary antibody were included for each staining run. Stained slides were dehydrated, cleared, and mounted prior to imaging with a brightfield microscope. Quantification was performed by calculating the number of positively stained cells per field.

Molecular docking analysis

The three-dimensional structure of the target protein was predicted using AlphaFold3 (DeepMind, Google, 2024 release). The structure of POSTN and ITGB1 was obtained either from the Protein Data Bank (PDB) or predicted using the same approach. The modeled structures were optimized and prepared for docking analysis. Protein–protein docking was performed using AlphaFold3's multimer module, which provides structure prediction of protein complexes based on deep learning algorithms. The resulting models were ranked by confidence scores (pLDDT and ipTM). The top-ranking models were selected for further evaluation.

Visualization and structural analysis of the docking complexes were conducted in PyMOL (version 2.5, Schrödinger, LLC). Hydrogen bonds, hydrophobic interactions, and binding interfaces were examined to identify the potential interaction regions between the candidate proteins.

His pull-down assay

Recombinant human POSTN protein carrying a His-tag (rhPOSTN-His) was incubated with cultured macrophages at 37 °C for 2 h to allow protein–cell interactions. After incubation, cells were harvested, washed three times with ice-cold PBS to remove unbound protein, and lysed in lysis buffer (20 mM Tris-HCl, 150 mM NaCl, 1% NP-40, protease inhibitor cocktail, pH 7.5). Clarified lysates were incubated with pre-equilibrated Ni-NTA agarose beads at 4 °C overnight. The beads were thoroughly washed with buffer (20 mM Tris-HCl, 300 mM NaCl, 20 mM imidazole, 0.1% NP-40, pH 7.5) to minimize nonspecific interactions, and bound proteins were eluted with an imidazole-containing elution buffer (250 mM). The eluted samples were resolved

by SDS-PAGE and subjected to immunoblotting using anti-His antibody to confirm the successful pull-down of rhPOSTN-His and its interacting proteins in macrophages.

Pharmacological inhibition assays

To investigate the functional involvement of the ITGB1–PI3K/AKT/mTOR signaling axis in POSTN-mediated metabolic reprogramming, cells were treated with the PI3K inhibitor Alpelisib, the AKT inhibitor Capivasertib, or the mTOR inhibitor Everolimus at the indicated concentrations prior to rhPOSTN stimulation. For oxidative stress intervention experiments, cells were pretreated with the antioxidant N-acetyl-L-cysteine (NAC) or the lipid peroxidation inhibitor Ferrostatin-1 (Fer-1) before exposure to rhPOSTN.

siRNA library screening by parallel transfection

siRNA-mediated knockdown of nineteen integrin genes (ITGA1, ITGA2, ITGA3, ITGA4, ITGA5, ITGA6, ITGA7, ITGAE, ITGAL, ITGAM, ITGAV, ITGAX, ITGB1, ITGB2, ITGB3, ITGB4, ITGB5, ITGB7, ITGB8) was performed in parallel using a 96-well plate format. The corresponding siRNA sequences are provided in Table S6. Individual siRNAs were arrayed across wells with each target assayed in technical duplicate and in at least three independent biological replicates; non-targeting siRNA served as a negative control. For reverse transfection, siRNAs, final concentration 20 nM, were diluted in Opti-MEM and complexed with Hieff Trans® in vitro siRNA/miRNA Transfection Reagent (Yeasen, Cat# 40806ES03) according to the manufacturer's instructions, incubated at room temperature for 15 min, and dispensed into 96-well plates. Cells were trypsinized, counted and seeded directly onto the siRNA-reagent complexes at a density of 5000 cells/well in 100 µL complete medium. Plates were maintained at 37 °C with 5% CO_2, and knockdown efficiency was assessed by qRT-PCR 48 h after transfection.

For ITGB1 rescue experiments, silent mutations were introduced into the siITGB1 target sequence without altering the amino acid sequence, generating a siRNA-resistant ITGB1 expression construct (ITGB1-resistant). Cells were co-transfected with siITGB1 and ITGB1-resistant plasmids before subsequent lipid metabolism and oxidative stress analyses.

Multiplex Immunofluorescence (mIF) Staining

Tissue microarrays (TMAs) were constructed by Shanghai Outdo Biotech Company (Shanghai, China) according to standard protocols. Human GC TMAs were sectioned, deparaffinized in xylene, and rehydrated through a graded ethanol series. Antigen retrieval was performed in 10 mM citrate buffer (pH 6.0) via microwave treatment. Endogenous peroxidase was inhibited with 3% H_2O_2 for 10 min, and nonspecific binding was blocked with 5% normal serum for 30 min at room temperature. Sections were incubated sequentially with primary antibodies against POSTN, ITGB1, PPARG, CD163, and α-SMA (dilutions and clones listed in Supplementary Table 7), each followed by fluorophore-conjugated secondary antibodies using a tyramide signal amplification (TSA) system. Nuclei were counterstained with DAPI. After staining, slides were mounted with anti-fade medium and imaged using a fluorescence or confocal microscope.

Flow Cytometry of Tumor-Infiltrating Immune Cells in Mice

Tumor tissues from mice were mechanically dissociated, digested with collagenase IV and DNase I at 37 °C, filtered through 70-μm strainers, and treated with red blood cell lysis buffer to obtain single-cell suspensions. After Fc receptor blockade with anti-mouse CD16/32 antibody, cells were stained with fluorochrome-conjugated antibodies using three panels: for $CD8^+$ T cells, eFluor 450 viability dye, Alexa Fluor 700–CD45, APC–CD3, and FITC–CD8a; for $CD4^+$ T cells, eFluor 450 viability dye, Alexa Fluor 700–CD45, APC–CD3, PE-Cy7–CD4, and PE–CD25; for macrophages, eFluor 450 viability dye, Alexa Fluor 700–CD45, FITC–CD11b, APC-Cy7–F4/80, PE–CD86, and Alexa Fluor 647–CD206; for intracellular IFN-γ detection, eFluor 450 viability dye, Alexa Fluor 700–CD45, APC–CD3, FITC–CD8a, PE-Cy7–CD4, and PE–IFN-γ; and for intracellular Granzyme B detection, eFluor 450 viability dye, Alexa Fluor 700–CD45, APC–CD3, FITC–CD8a, PE–CD4, and PE-Cy7–Granzyme B.Data were acquired on a BD LSRFortessa flow cytometer and analyzed with FlowJo software. Live $CD45^+$ leukocytes were selected for analysis, with $CD3^+CD8a^+$ and $CD3^+CD4^+$ populations gated as tumor-infiltrating T cells, $CD25^+$ cells within the $CD4^+$ compartment identified as activated $CD4^+$ T cells, and $CD11b^+F4/80^+$ cells defined as macrophages, which were further subdivided into M1 ($CD86^+$) and M2 ($CD206^+$) subsets. Appropriate fluorescence-minus-one and isotype controls were included to set gates.

TEM Measurement

For TEM analysis, 10 µL of each Lipid nanoparticles (LNP) sample was deposited onto carbon-coated copper grids (Ted Pella, Inc., USA; 300 mesh, 3.0 mm O.D.) pre-treated with argon plasma (420 V, 1 min) for 30 s. Residual solvent was blotted with filter paper, and grids were sequentially washed and stained with 5 µL of 1% (w/v) uranyl formate twice, each for 5 s, before being air-dried at room temperature. Imaging was performed on a JEOL JEM-1100 electron microscope (JEOL, Tokyo, Japan) operating at 80 kV.

Agarose Gel Shift Assay

A 2.5% (w/v) agarose gel was prepared in hot 1× TBE buffer (10.8 g Tris base, 5.5 g boric acid, 0.75 g EDTA, pH 8, per liter) and cooled to approximately 50 °C. GelRed was added for staining, and the solution was cast into a gel tray with a comb, which was removed after solidification. Samples were combined with loading buffer (6 mL glycerol, 1.2 mL 0.5 M EDTA, 2.8 mL H_2O, 0.02 g bromophenol blue) at a 5:1 (v/v) ratio to reach 24 µL, loaded into the gel wells, and electrophoresed in 1× TBE at 120 V for 70 min. Free siRNA in buffer at equivalent concentration served as a positive control, while buffer alone was used as a negative control. To assess serum stability, LNPs were pre-incubated in buffer containing 90% (v/v) FBS for 2 h at 37 °C prior to electrophoresis. Bands were visualized using a UV transilluminator (Biostep, Germany).

LNP characterization

LNPs encapsulating siPostn were prepared using a microfluidic mixing method as previously described. Particle size distribution, polydispersity index (PDI), and zeta potential were measured using dynamic light scattering (DLS) on a Zetasizer Pro instrument (Malvern Panalytical Company).

Single-cell data analysis

ScRNA-seq data were first processed using the Seurat package to filter cells based on the following criteria: (1) detection of 500-6000 genes, (2) fewer than 30,000 UMI counts per cell, and (3) less than 50% mitochondrial gene content. The data were then normalized and scaled, and the top 2000 highly variable genes were identified using the FindVariableFeatures function. Principal component analysis (PCA) was performed using these variable genes, selecting 50 principal components for subsequent clustering. Batch effects across cohorts were removed using the Harmony package. Cell subclusters

were identified with the FindNeighbors and FindClusters functions, setting a resolution of 1 for major cell types and 0.3 for fibroblasts. Finally, UMAP was used for cell visualization. Cell annotations were primarily based on widely accepted cell type- specific markers. Differential expression analyses between cell populations were performed using the Wilcoxon rank-sum test implemented in Seurat. P values were adjusted for multiple comparisons using the Benjamini–Hochberg procedure, and genes with FDR < 0.05 were considered significant. Pathway enrichment analyses were performed using clusterProfiler.

siRNA-LNP Formulation

Preparation of Lipid Stock Solutions

The individual lipid components—SM-102, cholesterol, DSPC, and DMG-PEG—were accurately weighed and separately dissolved in anhydrous ethanol to achieve specific stock concentrations. Cholesterol (CHO-HP, Avanti) was dissolved at 34.48 mg/mL in 5 mL of ethanol, with gentle heating at 55°C to facilitate complete dissolution. DSPC was prepared at 15.9 mg/mL in 5 mL of ethanol, while DMG and SM-102 were dissolved in 3 mL of ethanol to final concentrations of 15.4 mg/mL and 36.8 mg/mL, respectively. All solutions were mixed by inversion until visually clear and stored at -80°C in single-use aliquots to avoid repeated freeze-thaw cycles.

Formulation of Lipid Nanoparticles (LNPs)

For standard LNP preparation, the calculated volumes of each lipid stock solution were combined to achieve a molar ratio of 50:38.5:10:1.5 (SM-102 : cholesterol : DSPC : DMG-PEG). To prepare 1 mL of the organic phase, 74.25 μL of cholesterol, 85.53 μL of DSPC, 42.2 μL of DMG, and 166.03 μL of SM-102 stock solutions were mixed, and the volume was brought to 1 mL with anhydrous ethanol. For cRGD-functionalized LNPs, DMG-PEG was partially substituted with DSPE-PEG-cRGD at varying molar percentages (10%, 15%, 20%, 30%, and 40%), with adjustments made to the respective lipid masses accordingly.

Microfluidic Assembly of siRNA-LNP Complexes

Prior to assembly, siRNA (100 μg) was reconstituted in DEPC water, and the LNP lipid mixture was thawed at 37°C for 15 minutes. The aqueous phase consisted of siRNA diluted in 60 μL of 10× buffer and brought to a total volume of 600 μL with DEPC water. This aqueous phase was mixed with 200 μL of the organic phase (LNP solution) at a 3:1 flow rate ratio using a microfluidic instrument

(MicroANNA system). The system was primed with ethanol and DEPC water before each run, and samples were collected after processing. Between samples, the system was rigorously cleaned using a sequential wash with ethanol and DEPC water to prevent cross-contamination.

Purification and Buffer Exchange

The assembled LNPs were purified using a 100 kDa molecular weight cut-off centrifugal filter. The filter was pre-rinsed with 3 mL of exchange buffer, after which the LNP solution was loaded and centrifuged at 1,200 × g for 45 minutes. The filtrate was discarded, and the step was repeated twice with fresh buffer to remove residual ethanol and unencapsulated siRNA. The final concentrated LNPs were recovered in the desired buffer for downstream applications.

Determination of Encapsulation Efficiency

Encapsulation efficiency was quantified using a Qubit® RNA assay. To measure free RNA, 5 μL of the purified LNP sample was added to 194 μL of Qubit buffer and 1 μL of dye. To determine total RNA, 1 μL of the sample was mixed with 4 μL of 2% Triton X-100 (to disrupt the LNPs), 194 μL of buffer, and 1 μL of dye. The fluorescence was measured, and encapsulation efficiency was calculated as follows: EE% = (1 − free RNA / total RNA) × 100%. All prepared LNPs were stored at 4°C and used within 24 hours to ensure stability.

Untargeted Metabolomics

Metabolite Extraction from Mouse Subcutaneous Xenograft Tumor Tissues

First, 100 mg of mouse subcutaneous xenograft tumor tissue was collected and immediately placed in a prechilled mortar. Liquid nitrogen was continuously added to the mortar during the grinding process to keep the tissue in a frozen state, and the tissue was ground thoroughly into a fine powder to ensure that no obvious tissue fragments remained. The ground tissue powder was transferred to a prechilled Eppendorf tube, and an appropriate volume of prechilled 80% methanol solution was added to the tube. The tube was then placed on a vortex oscillator, and the mixture was vortexed vigorously for 30 seconds to ensure that the tissue powder was fully suspended in the 80% methanol solution and that metabolites in the tissue were sufficiently dissolved into the solution. After vortexing, the Eppendorf tube was placed on ice for incubation, and the incubation time was strictly controlled for 5 minutes; during the incubation period, the tube was gently shaken once every 1 minute to promote the release of metabolites and avoid local precipitation of the tissue mixture.

After the ice incubation, the Eppendorf tube was placed in a high-speed centrifuge, and the centrifuge parameters were set to 15,000×g and 4°C. The centrifugation process lasted for 20 minutes to separate the tissue debris and the supernatant containing metabolites. After centrifugation, the centrifuge was slowly decelerated to avoid disturbing the separated supernatant and precipitate. A sterile pipette tip was used to carefully aspirate the upper supernatant into a new sterile Eppendorf tube; during aspiration, attention was paid to avoiding contact with the lower precipitate to prevent tissue debris from contaminating the supernatant. Then, LC-MS grade water was added to the aspirated supernatant, and the mixture was gently inverted and mixed to dilute the supernatant to a final methanol concentration of 53%. The dilution process was carried out slowly to ensure uniform mixing of the solution and avoid the generation of air bubbles that might affect subsequent centrifugation.

The diluted supernatant was transferred to a fresh Eppendorf tube, which was then placed in the high-speed centrifuge again. The centrifuge parameters were maintained at 15,000×g and 4°C, and centrifugation was performed for another 20 minutes to further remove any tiny tissue debris or impurities that might remain in the diluted supernatant. After the second centrifugation, the supernatant was carefully aspirated once more, and this supernatant was the final metabolite extract to be used for subsequent LC-MS/MS analysis.

UHPLC-MS/MS Analysis

The UHPLC-MS/MS analysis was conducted using a Vanquish UHPLC system (ThermoFisher, Germany) coupled with an Orbitrap Q Exactive™ HF mass spectrometer, Orbitrap Q Exactive™ HF-X mass spectrometer, or Orbitrap Exploris™ 120 mass spectrometer (Thermo Fisher, Germany)[2,3]. Before sample injection, the entire UHPLC-MS/MS system was preheated and stabilized according to the instrument operation manual; the preheating time was no less than 30 minutes to ensure that the column temperature, flow rate, and mass spectrometer parameters reached stable states.

A Hypersil Gold column (100×2.1 mm, 1.9 μm) was used for chromatographic separation. Before sample loading, the column was equilibrated with the initial mobile phase (2% eluent B and 98% eluent A) at a flow rate of 0.2 mL/min for 10 minutes to ensure that the column was fully balanced and that the baseline of the chromatogram was stable. The eluents used for the analysis were eluent A (0.1% formic acid in water) and eluent B (methanol); both eluents were filtered through a 0.22 μm organic phase filter membrane before use and degassed under vacuum for 30 minutes to remove dissolved air, which could prevent the generation of bubbles in the chromatographic system and avoid affecting the

separation effect.

The metabolite extract obtained from the previous step was injected into the UHPLC system using an autosampler, and the injection volume was set to 5 μL. The chromatographic separation was carried out using a 12-minute linear gradient program: from 0 to 1.5 minutes, the proportion of eluent B was maintained at 2% to ensure the initial separation of low-polarity metabolites; from 1.5 to 4.5 minutes, the proportion of eluent B was linearly increased from 2% to 85% to separate metabolites with medium polarity; from 4.5 to 14.5 minutes, the proportion of eluent B was continuously increased from 85% to 100% to elute high-polarity metabolites; at 14.5 to 14.6 minutes, the proportion of eluent B was rapidly decreased from 100% to 2% to quickly restore the initial mobile phase composition; from 14.6 to 26.5 minutes, the proportion of eluent B was maintained at 2% to re-equilibrate the column for the next sample analysis.

The mass spectrometer was operated in both positive and negative polarity modes to detect more types of metabolites. The key parameters of the mass spectrometer were set as follows: the spray voltage was 3.5 kV, which ensured the effective ionization of metabolites; the capillary temperature was 320°C to promote the evaporation of the mobile phase and the ionization of samples; the sheath gas flow rate was 35 psi, which could protect the capillary and improve the ionization efficiency; the aux gas flow rate was 10 L/min to assist in the evaporation of the sample; the S-lens RF level was 60 to enhance the transmission efficiency of ions; the aux gas heater temperature was 350°C to further assist in the evaporation and ionization of the sample. The mass scanning range was set to m/z 70-1050 to cover most of the common metabolites, and the resolution was set to 70,000 for full scanning and 17,500 for secondary scanning to ensure the accuracy of metabolite mass detection and the reliability of fragment ion information. During the analysis process, quality control samples were injected every 10 samples to monitor the stability of the instrument and the reliability of the analysis results; if the signal intensity of the quality control samples changed by more than 10%, the instrument was recalibrated and the analysis was restarted.

Metabolomic data analysis

Peak alignment, retention time correction, and metabolite annotation were performed according to standard workflows. Metabolite intensities were normalized prior to downstream analyses. Principal component analysis (PCA) was used to evaluate global metabolic variation. Differential metabolites between groups were identified using Student's t-test with Benjamini–Hochberg correction. Metabolites

with FDR < 0.05 were considered significantly altered. KEGG pathway enrichment analysis was subsequently performed using identified differential metabolites.

Immune scoring and immune cell quantification

Using the “ESTIMATE” package in R software, immune scores were calculated based on the RNA expression profiles of tumor tissues. This score evaluates the level of immune response in each tumor sample according to immune gene expression characteristics. Next, single-sample gene set enrichment analysis (ssGSEA) was performed using the “GSVA” and “GSEABase” packages in R software to quantify the infiltration of 28 types of immune cells in each STAD sample. The characteristic genes of these 28 types of immune cells were derived from published studies. Finally, the “ggplot2” package in R software was used to generate heatmaps of immune cells to visually display their relative abundance in different STAD samples.

Prediction of immune checkpoint inhibitor treatment response in TCGA dataset

The TIDE algorithm was used to evaluate the potential efficacy of immune checkpoint inhibitor treatment in the TCGA dataset. TIDE (http://tide.dfci.harvard.edu/) predicts the response to immune checkpoint inhibitors by utilizing tumor immune evasion mechanisms, including calculating the levels of infiltrating $CD4^+$ T cells and $CD8^+$ T cells in tumor tissues.

Statistical analysis

Statistical analyses were performed using GraphPad Prism 9.0 (GraphPad Software) and R software (version 4.2.0) unless otherwise specified. Data are presented as mean ± SD unless indicated otherwise in the figure legends. All in vitro experiments were independently repeated at least three times. Statistical significance between two groups was assessed using two-tailed Student’s t-test, whereas comparisons among multiple groups were performed using one-way analysis of variance (ANOVA) followed by appropriate post hoc multiple-comparison tests. For nonparametric datasets, Kruskal–Wallis tests followed by Dunn’s multiple comparison correction were applied. A two-sided P value < 0.05 was considered statistically significant.

Transcriptomic and spatial omics analyses

For single-cell RNA sequencing and spatial transcriptomics analyses, differential gene expression

analyses were performed using the Wilcoxon rank-sum test implemented in Seurat. Multiple hypothesis testing was corrected using the Benjamini–Hochberg method, and genes with an adjusted false discovery rate (FDR) < 0.05 were considered statistically significant.

For bulk RNA-sequencing analyses, differential expression analysis was performed using DESeq2. Genes with an absolute log2 fold change ($|\log 2FC| > 1$) and FDR < 0.05 were considered differentially expressed. Gene set enrichment analysis (GSEA) and pathway enrichment analyses were performed using clusterProfiler, with statistical significance determined based on normalized enrichment scores (NES) and Benjamini–Hochberg-adjusted P values (FDR q-values).

For high-dimensional analyses, including pathway enrichment, CellChat signaling analysis, and gene-level screening approaches, multiple comparisons were corrected using the Benjamini–Hochberg method to control the false discovery rate.

Metabolomic analysis

For metabolomic analyses, peak intensities were normalized before downstream statistical analyses. Differential metabolites were identified using univariate statistical testing with Benjamini–Hochberg correction for multiple comparisons. Metabolites with FDR < 0.05 and predefined fold-change thresholds were considered significantly altered.

Survival and correlation analyses

Survival analyses were performed using Kaplan–Meier methods, and differences between survival curves were evaluated using log-rank tests. Hazard ratios were estimated using Cox proportional hazards regression models where indicated.

Correlation analyses between POSTN expression and stromal/immune-related features were performed using Spearman correlation analysis unless otherwise indicated. Correlation coefficients (r) and corresponding P values were reported. Associations between POSTN expression and clinical outcomes were evaluated using survival analysis models as described above.

References

[1] Yamamoto M, Nomura S, Hosoi A, Nagaoka K, Iino T, Yasuda T, et al. Established gastric cancer cell lines transplantable into C57BL/6 mice show fibroblast growth factor receptor 4 promotion of tumor growth. Cancer Sci 2018;109:1480–92.

[2] Sellick CA, Hansen R, Stephens GM, Goodacre R, Dickson AJ. Metabolite extraction from suspension-cultured mammalian cells for global metabolite profiling. Nat Protoc 2011;6:1241–9.

[3] Yuan M, Breitkopf SB, Yang X, Asara JM. A positive/negative ion-switching, targeted mass spectrometry-based metabolomics platform for bodily fluids, cells, and fresh and fixed tissue. Nat Protoc 2012;7:872–81.

Supplementary figures

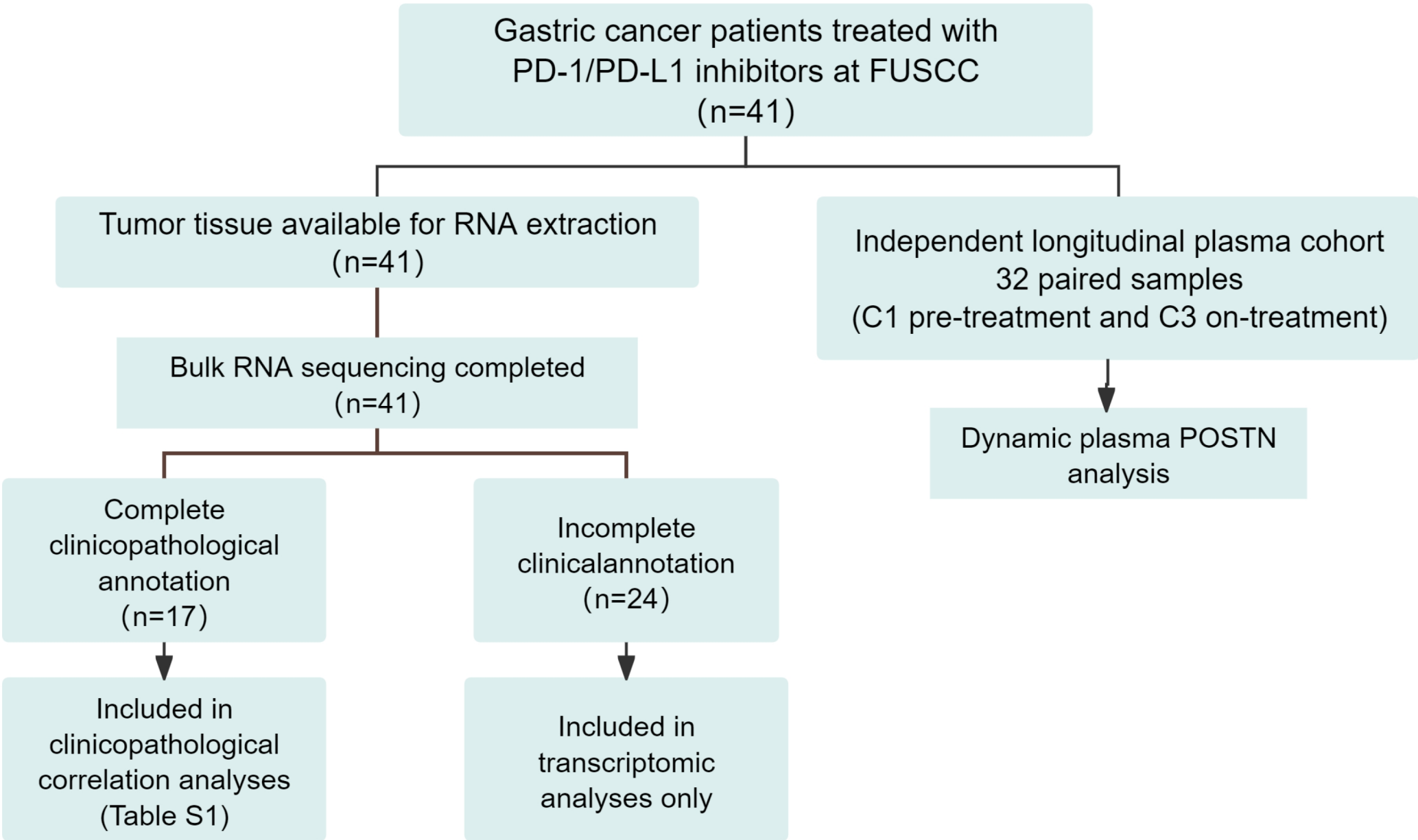

Figure S1. Flowchart of patient enrollment in the clinical cohort.

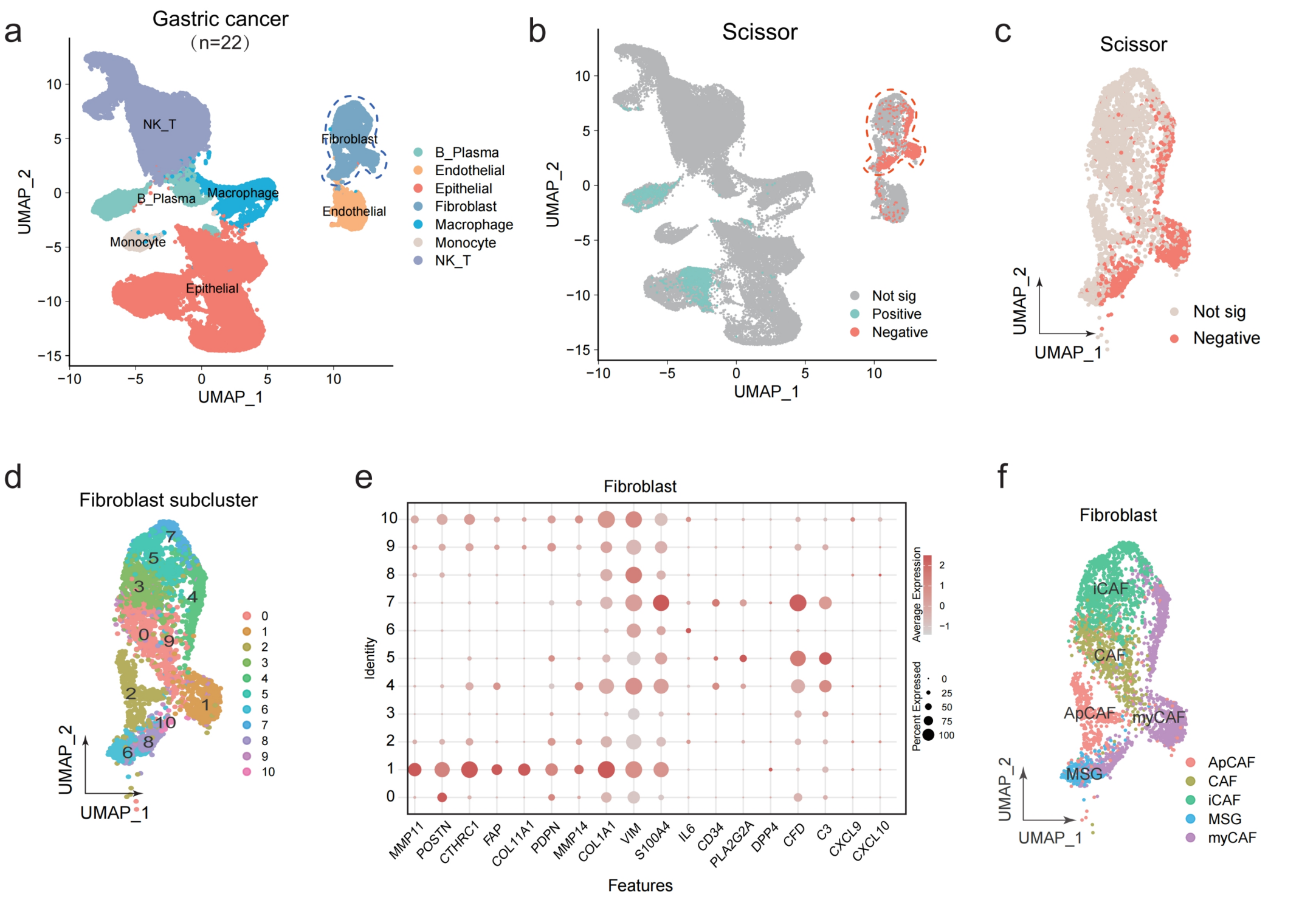

Figure S2. (related to Fig. 1)

Single-cell transcriptomic landscape and fibroblast subpopulation annotation in gastric cancer (GC). (a) UMAP visualization of scRNA-seq data from GC tissues, showing major cell clusters: B_Plasma, Epithelial, Fibroblast, Macrophage, Monocyte, T/NK, and Endothelial cells. **(b)** UMAP plot highlighting "Scissor" subpopulations with positive (Scissor^{+}) and negative (Scissor^{-}) correlation. **(c)** UMAP of the Scissor subpopulation, color-coded by negative (Scissor^{-}) correlation. **(d)** UMAP of fibroblast cells, displaying 10 distinct subclusters. **(e)** Dot plot of marker gene expression across fibroblast subclusters. Dot size represents the percentage of cells expressing each feature, and color intensity indicates average expression levels. **(f)** UMAP of fibroblast subclusters annotation.

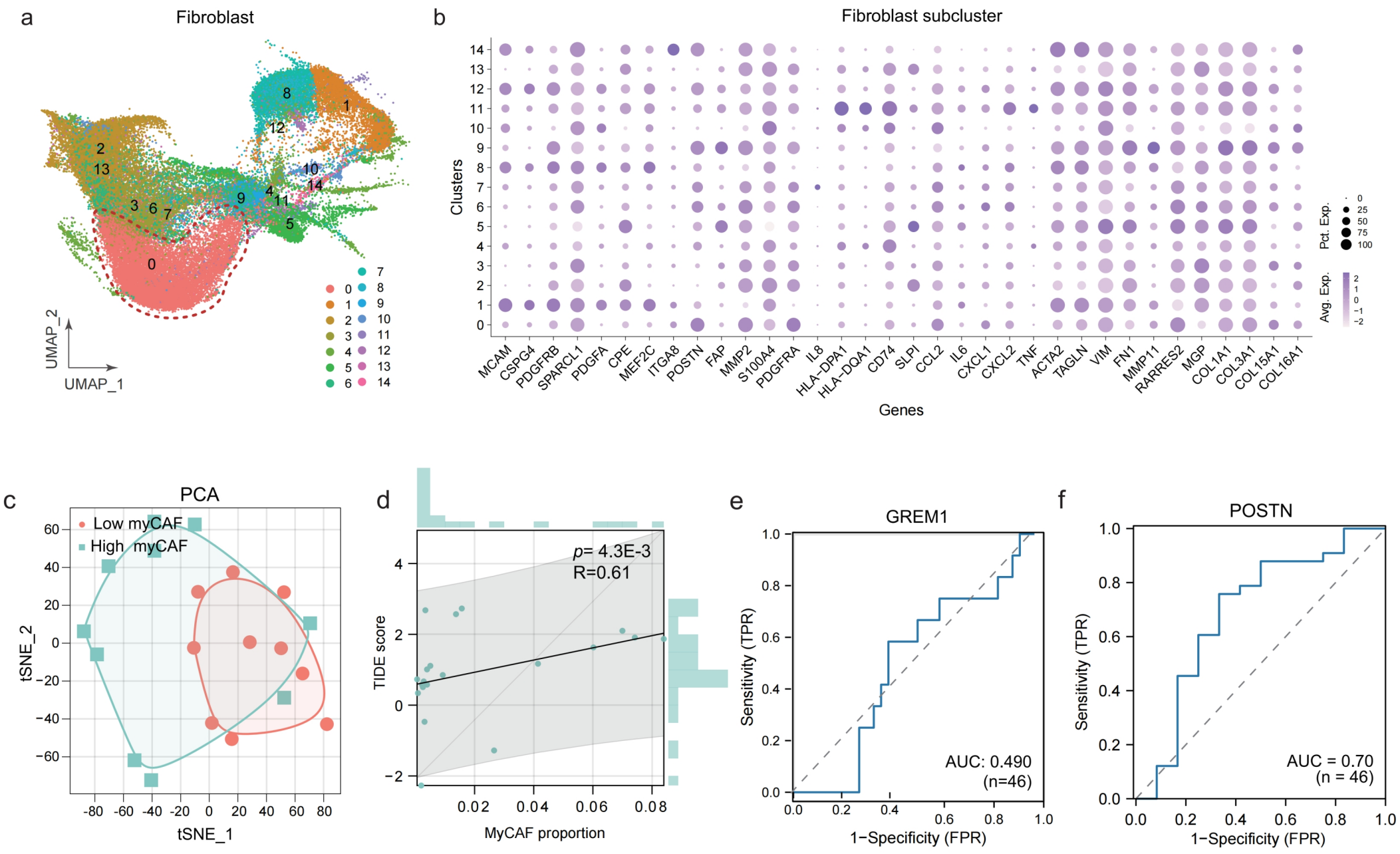

Figure S3. (related to Fig. 1)

Characterization of MyCAF abundance in GC. (a) UMAP visualization of fibroblast subclusters (0–14) color-coded by cluster identity. **(b)** Dot plot displaying the expression of representative marker genes across fibroblast subclusters. **(c)** Principal component analysis (PCA) of samples stratified by low versus high MyCAF abundance, showing distinct clustering patterns. **(d)** Scatter plot with linear regression showing the correlation between MyCAF abundance and TIDE (Tumor Immune Dysfunction and Exclusion) score. **(e)** Receiver operating characteristic (ROC) curve evaluating the predictive ability of GREM1 for MyCAF presence (AUC = 0.490, n = 46). **(f)** ROC curve evaluating POSTN as a predictor of MyCAF presence (AUC = 0.70, n = 46).

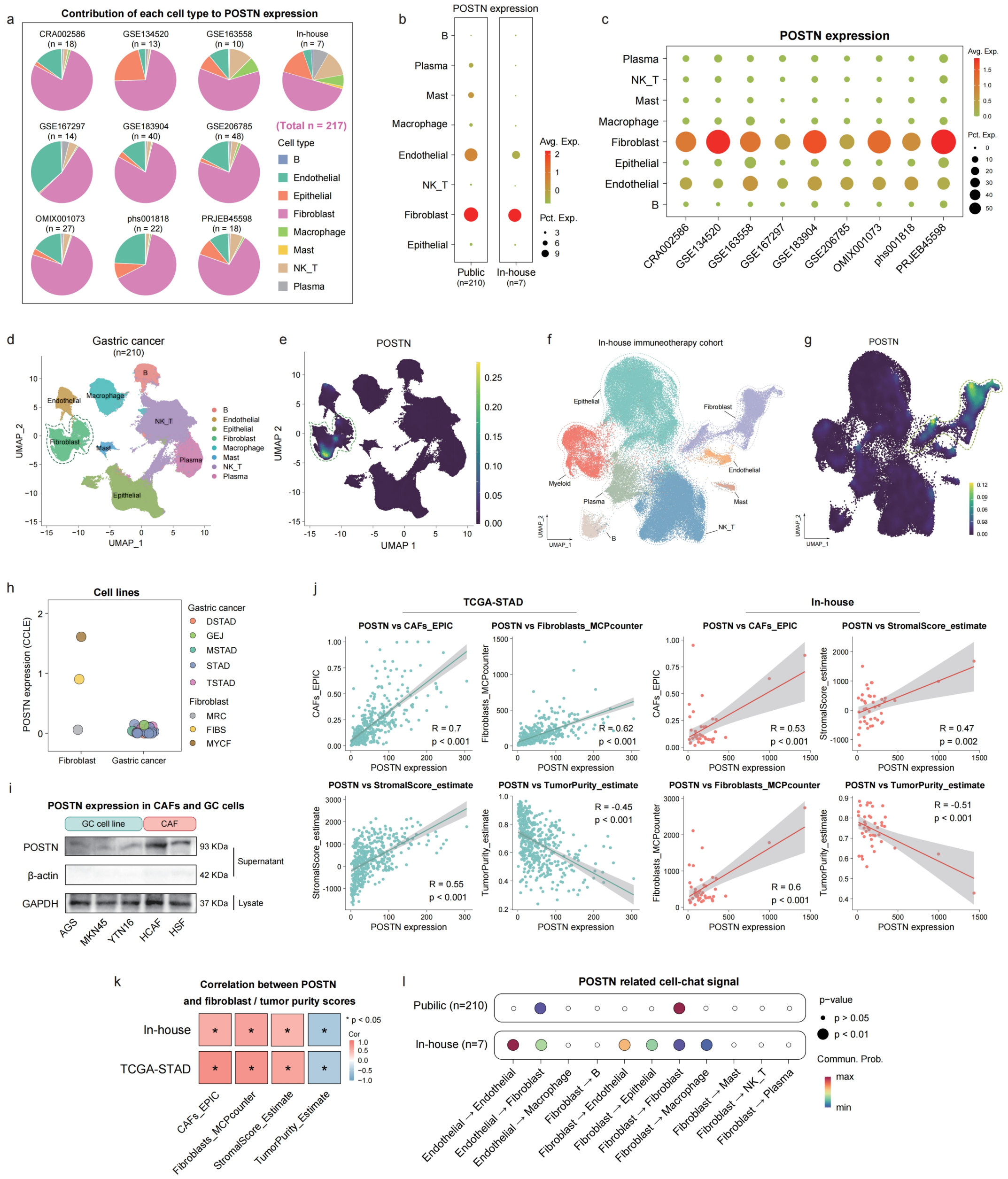

Figure S4. (related to Fig. 1)

Multi-omics evidence supports fibroblasts as the predominant source of POSTN in gastric cancer. (a) Proportion of POSTN-expressing cells across multiple independent public and in-house single-cell RNA sequencing datasets. **(b–c)** POSTN expression patterns across different cell populations in multiple public and in-house single-cell RNA sequencing datasets. **(d–g)** UMAP visualization showing the distribution of POSTN-expressing cells across different cell populations in multiple single-cell RNA sequencing datasets. **(h)** POSTN expression levels in gastric cancer cell lines and fibroblast cell lines. **(i)** POSTN protein concentrations in conditioned

media collected from gastric cancer cells and fibroblasts. **(j–k)** Correlation analysis between POSTN expression and CAF abundance or tumor purity estimated by computational deconvolution approaches. **(l)** CellChat analysis illustrating POSTN-related ligand–receptor signaling interactions among different cell populations.

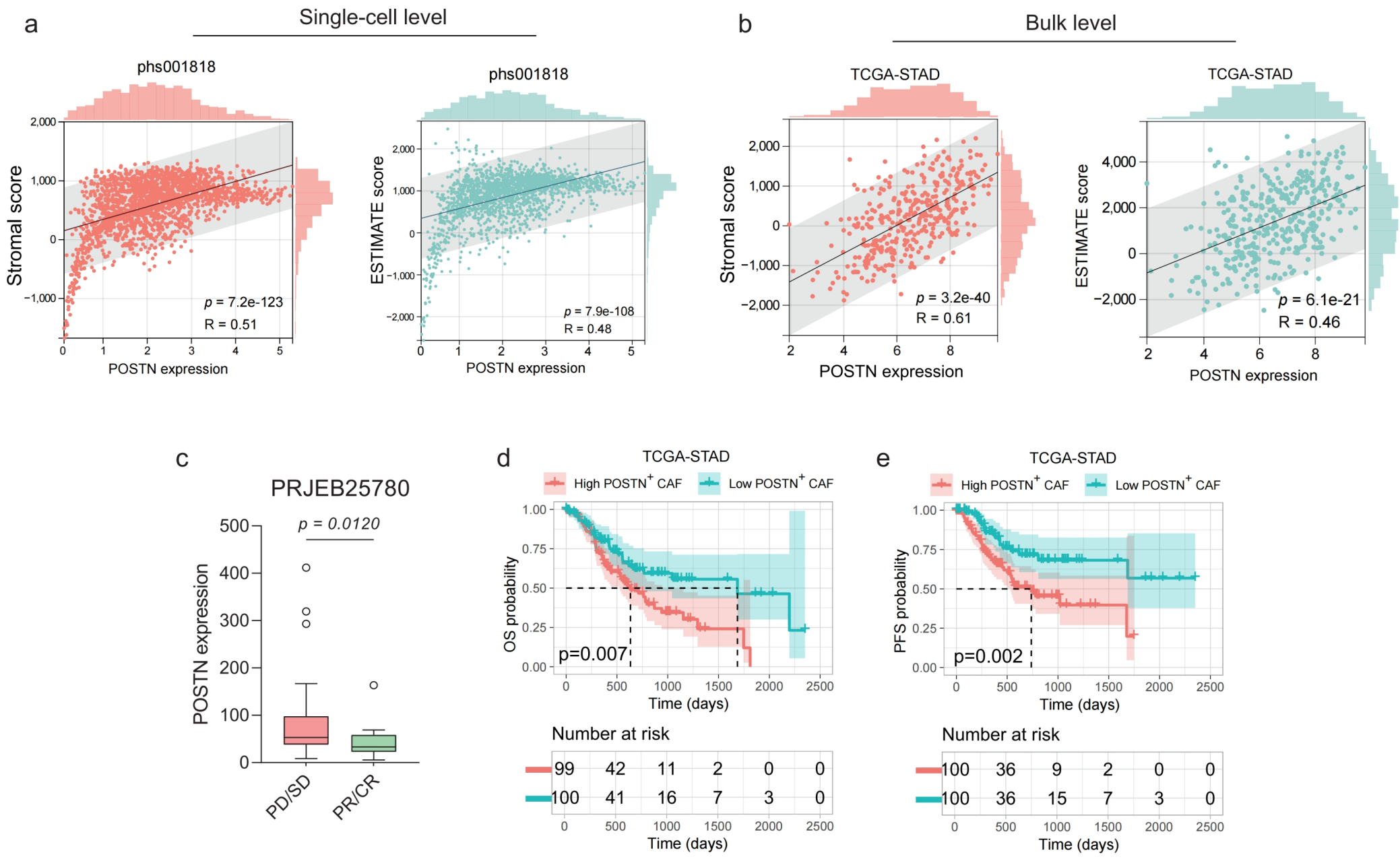

Figure S5. (related to Fig. 1)

Clinical relevance of MyCAF abundance in GC. (a) Single-cell–level scatter plots showing correlations between POSTN expression and stromal score (left) or ESTIMATE score (right) in phs001818 cohort. **(b)** Bulk-level scatter plots demonstrating correlations between POSTN expression and stromal score (left) or ESTIMATE score (right) in TCGA-STAD cohort. **(c)** Box plot comparing POSTN mRNA expression between PD/SD and PR/CR groups in the PRJEB25780 cohort. **(d–e)** Kaplan–Meier survival analysis of overall survival (OS, j) and progression-free survival (PFS, k) in the TCGA-STAD cohort, stratified by POSTN^{+} CAF status. Table below shows number of patients at risk over time.

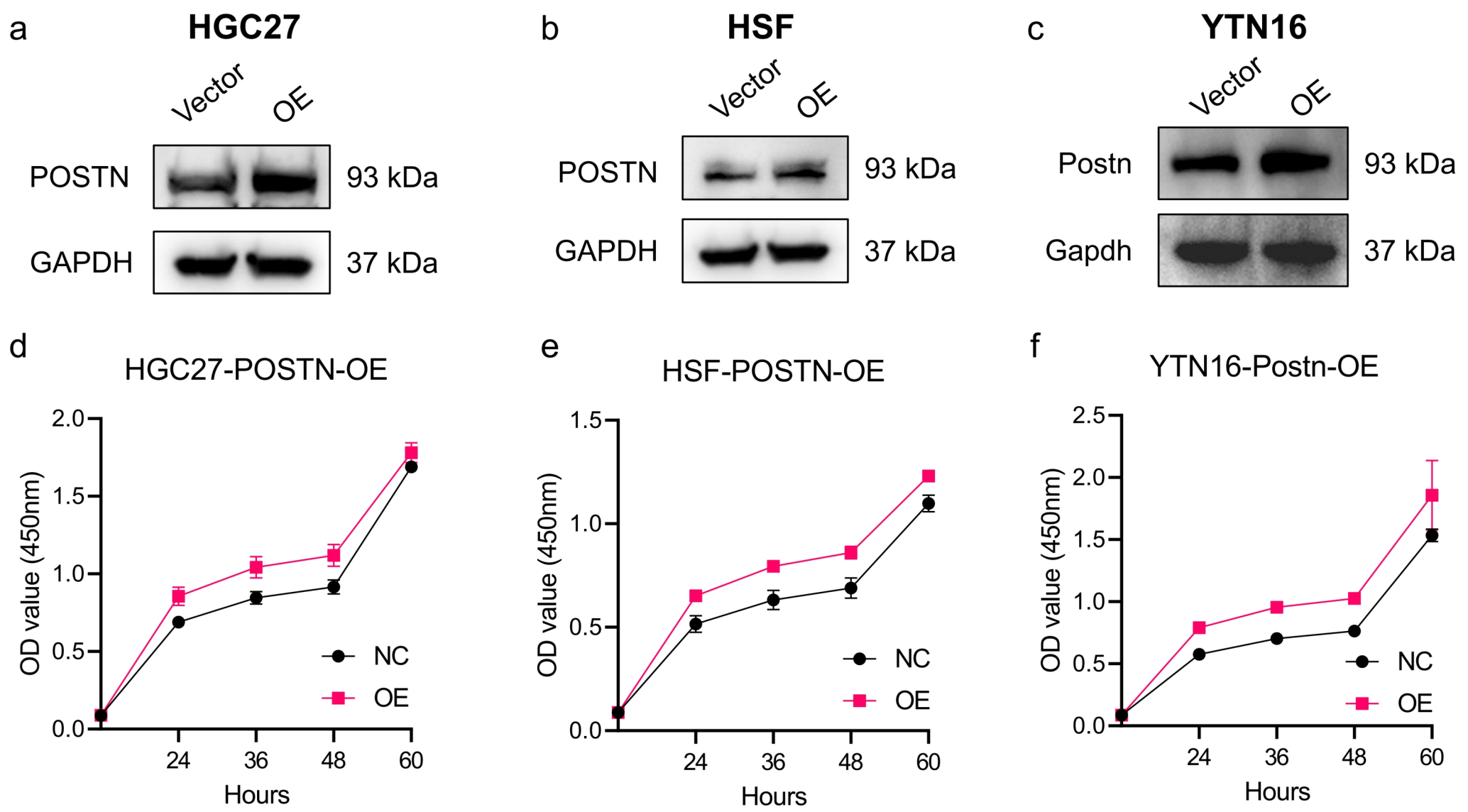

Figure S6. (related to Fig. 2)

Validation of POSTN overexpression and its effect on cell proliferation. (a–c) Western blot analysis confirming POSTN or Postn overexpression in HGC27 (a), HSF (b), and YTN16 (c) cells transfected with either empty vector (Vector) or POSTN-overexpressing vector (OE). GAPDH/Gapdh serves as a loading control. **(d–f)** CCK-8 proliferation assays showing growth curves of POSTN-overexpressing (OE) and negative control (NC) cells in HGC27 (d), HSF (e), and YTN16 (f) at 24, 36, 48, and 60 hours. Data are presented as mean ± SD.

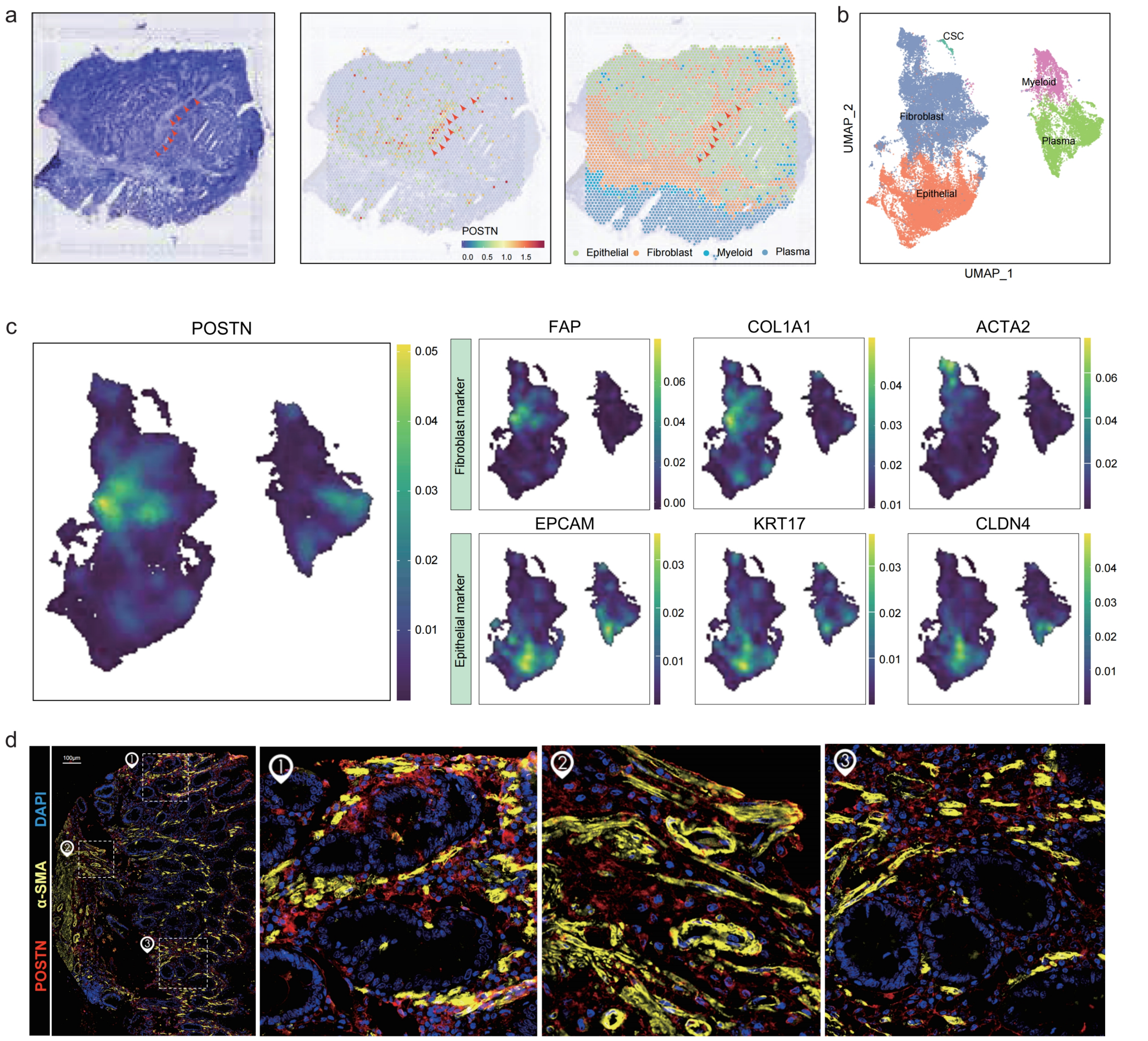

Figure S7. (related to Fig. 2)

Spatial and histological validation of POSTN localization within fibroblast-enriched regions of gastric cancer tissues. (a) Spatial distribution of fibroblast populations and POSTN expression patterns in gastric cancer tissue sections. **(b)** UMAP visualization of single-cell transcriptomic data corresponding to the spatial transcriptomic dataset. **(c)** Expression patterns of POSTN and representative fibroblast and epithelial cell markers in spatial transcriptomic sections. **(d)** Multiplex immunofluorescence staining demonstrating the spatial co-localization of POSTN with the fibroblast marker α-SMA in gastric cancer tissues.

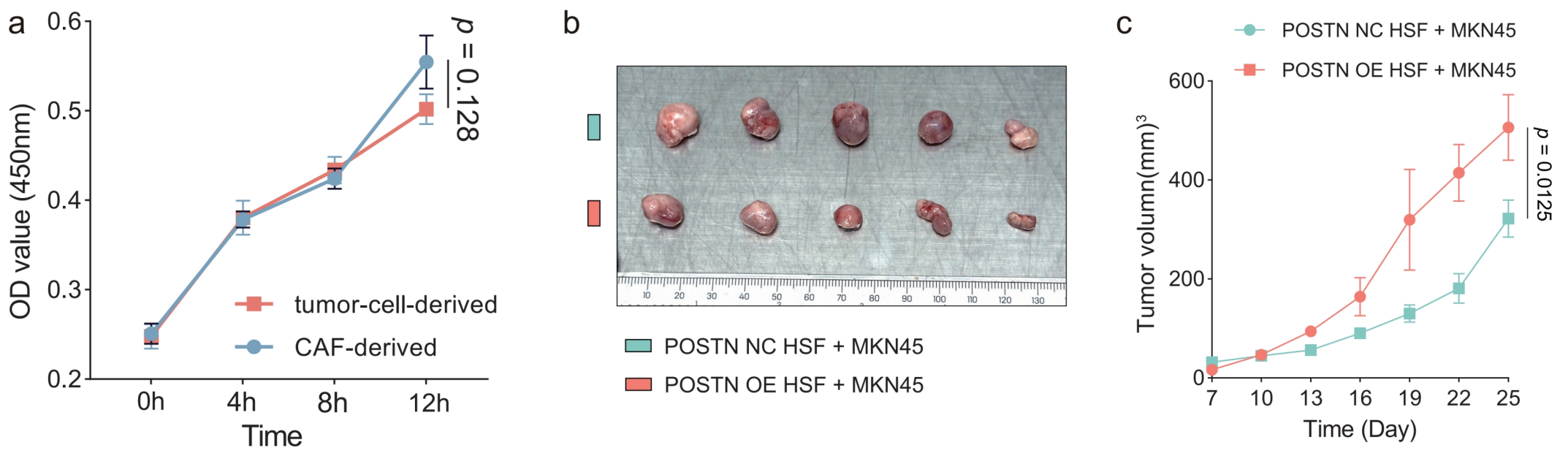

Figure S8. (related to Fig. 2)

Validation of POSTN overexpression and its effect on cell proliferation and tumor growth. (a) Comparison of the proliferative effects induced by tumor cell–derived and CAF-derived POSTN on GC cells. Unless otherwise indicated, data are presented as mean ± SD from three independent biological replicates (n = 3). **(b)** Representative images of excised tumors from mice subcutaneously co-implanted with MKN45 gastric cancer cells and either control HSF fibroblasts (NC) or POSTN-overexpressing HSF fibroblasts (POSTN-OE) (n = 5 mice per group). Species-matched human stromal validation that POSTN overexpression promotes gastric tumor growth in vivo. **(c)** Tumor growth curves of xenograft tumors in the indicated groups. Tumor volumes were measured at the indicated time points and plotted over the course of tumor progression. Data are presented as mean ± SD.

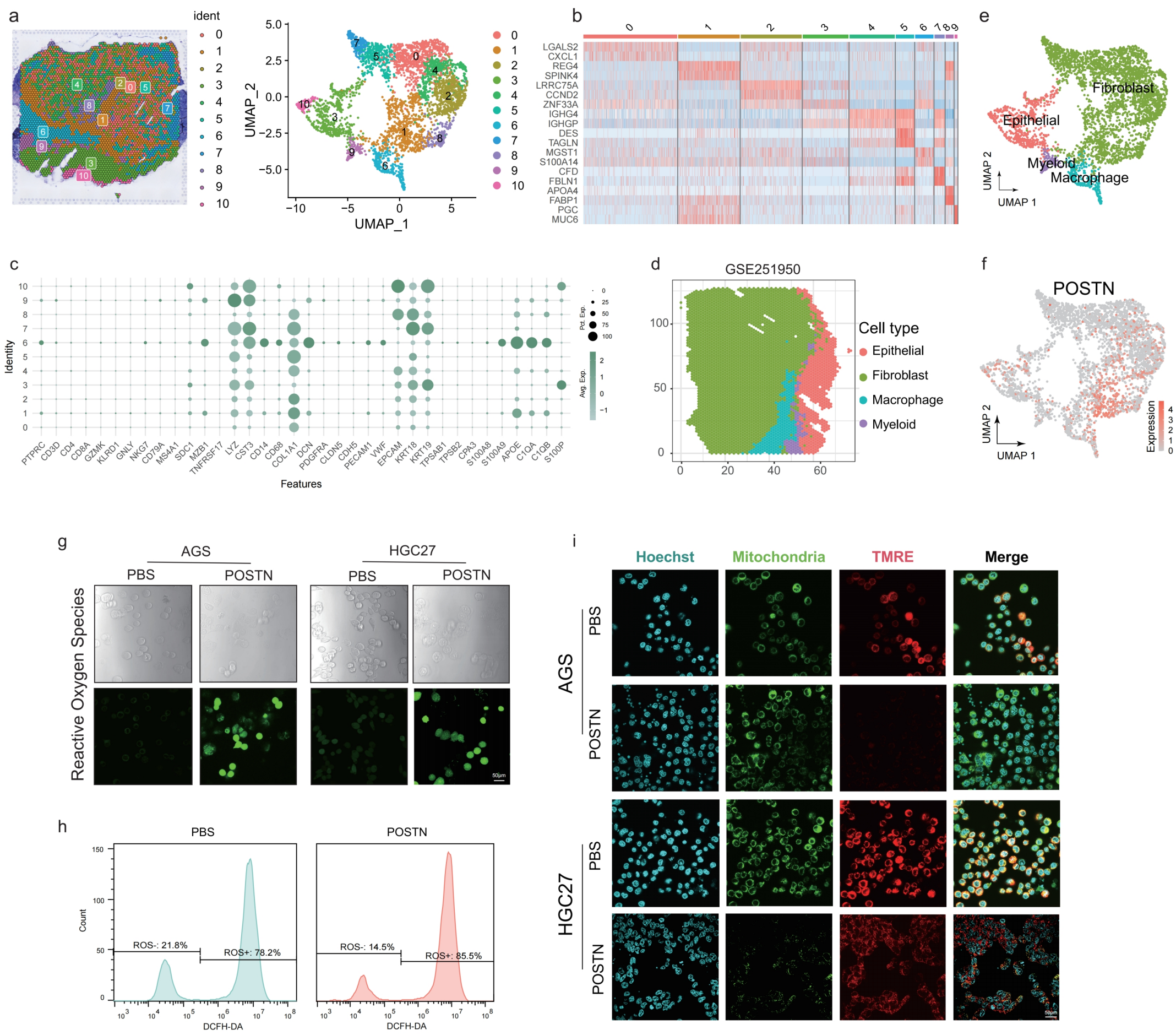

Figure S9. (related to Fig. 3)

Functional enrichment and spatial distribution of GC cell populations. (a) Spatial mapping of cell clusters in a representative GC tissue section (left) and corresponding UMAP visualization of single-cell transcriptomes (right), color-coded by cluster identity 0–10. **(b)** Heatmap of gene expression profiles across identified clusters. Rows represent genes, and columns represent clusters. Color scale indicates z-score–normalized expression. **(c)** Dot plot showing the expression of selected marker genes across cell clusters. Dot size reflects the proportion of expressing cells, while color intensity indicates average expression level. **(d)** Spatial distribution of cell clusters in a GC tissue section from the GSE26190 dataset, annotated by cell type. **(e)** UMAP plot highlighting Fibroblast, Epithelial, and Macrophage populations in the GSE26190 dataset. **(f)** UMAP plot illustrating POSTN expression, with color intensity corresponding to expression levels. **(g)** Representative bright-field (top) and fluorescence (bottom) microscopy images of AGS and HGC27 cells treated with PBS or rhPOSTN, stained for ROS (green). **(h)** Flow cytometry

histograms of DCFH-DA staining showing ROS levels in PBS- versus rhPOSTN-treated cells. Percentages of ROS-positive and ROS-negative populations are indicated. **(i)** Confocal microscopy images of AGS and HGC27 cells treated with PBS or rhPOSTN, stained with Hoechst (nuclei, cyan), MitoTracker (mitochondria, green), and TMRE (mitochondrial membrane potential, red). Merged panels demonstrate colocalization of mitochondrial signals.

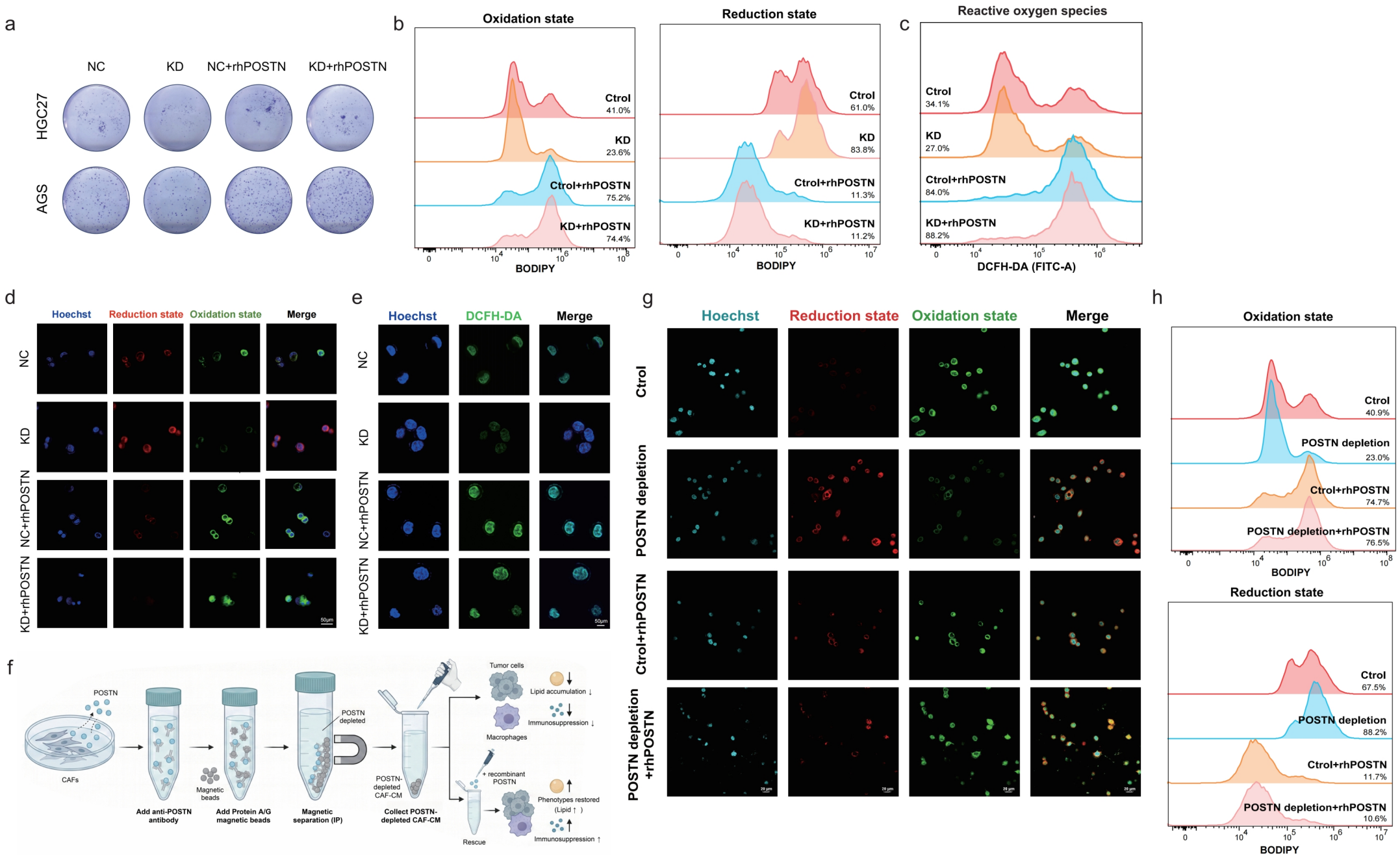

Figure S10. (related to Fig. 3)

Loss-of-function, rescue, and conditioned medium (CM) depletion experiments validate the specific role of POSTN in regulating lipid peroxidation and oxidative stress. (a) Colony formation assays showing the effects of POSTN knockdown (POSTN-KD) and recombinant POSTN (rhPOSTN) rescue on the clonogenic capacity of GC cells. Representative images and corresponding quantitative analyses are shown. **(b)** Flow cytometric quantification of intracellular lipid oxidation status in control, POSTN-KD, and rhPOSTN rescue groups using BODIPY 581/591 C11 staining. Oxidized and reduced lipid fractions are indicated. **(c)** Flow cytometric analysis of intracellular ROS levels in the indicated groups. **(d)** Representative fluorescence images of BODIPY 581/591 C11 staining showing changes in lipid oxidation following POSTN knockdown and rhPOSTN rescue. Green fluorescence represents oxidized lipids, whereas red fluorescence indicates reduced lipids. **(e)** Representative fluorescence images of intracellular ROS detection in control, POSTN-KD, and rhPOSTN rescue groups. **(f)** Schematic illustration of the antibody-mediated POSTN depletion strategy. CM derived from POSTN-overexpressing fibroblasts was incubated with an anti-POSTN antibody followed by Protein A/G magnetic bead capture to remove soluble POSTN. Recombinant POSTN was subsequently added back to generate the rescue condition. **(g)** Representative confocal images of BODIPY 581/591 C11 staining in GC cells treated with fibroblast-derived CM, POSTN-depleted CM, or

POSTN-depleted CM supplemented with rhPOSTN. **(h)** Flow cytometric quantification of lipid oxidation in GC cells following treatment with control CM, POSTN-depleted CM, or rhPOSTN-rescued CM.

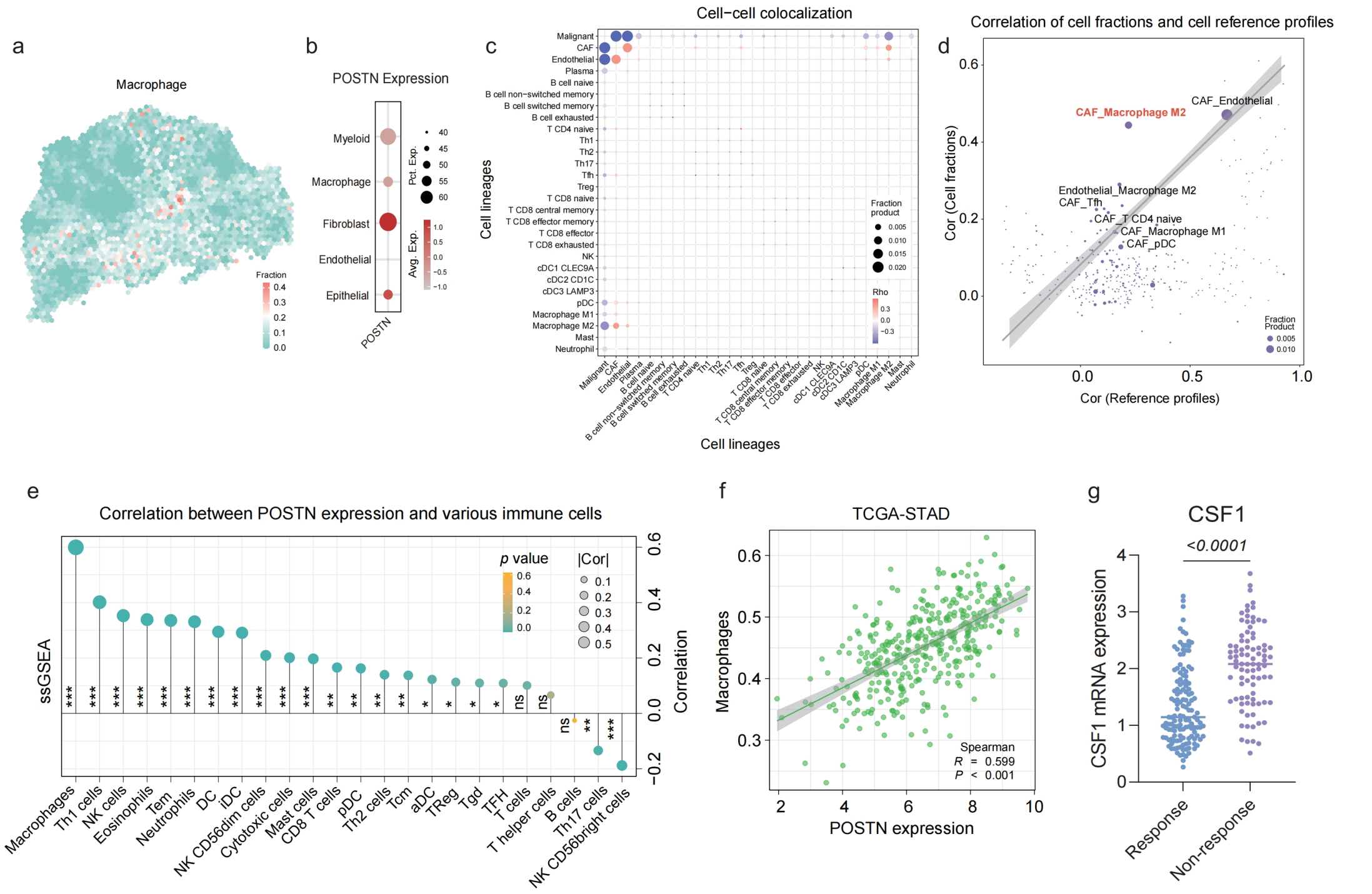

Figure S11. (related to Fig. 4)

Spatial association of POSTN$^+$ CAFs with macrophages and validation of POSTN-induced macrophage metabolic reprogramming. (a) Spatial mapping of macrophages in GC tissue sections, with color intensity representing local cell density. **(b)** Dot plot of POSTN expression across major cell subclusters. Dot size indicates the proportion of POSTN$^+$ cells; color intensity reflects average expression level. **(c)** Heatmap of cell–cell colocalization, with rows and columns denoting cell types. Color intensity indicates Spearman’s correlation coefficient, and dot size represents the product of cell fractions. **(d)** Scatter plot correlating cell fraction similarity (y-axis) with reference profile similarity (x-axis). Each point represents a cell lineage pair; key interactions (e.g., CAF_M2 macrophage) are highlighted. Point size denotes the fraction product. **(e)** Correlations between POSTN expression and immune cell infiltration in the TCGA-STAD dataset. **(f)** Scatter plot illustrating the correlation between POSTN expression and macrophage abundance. **(g)** Violin plot of CSF1 mRNA expression in immunotherapy respondersversus non-responders.

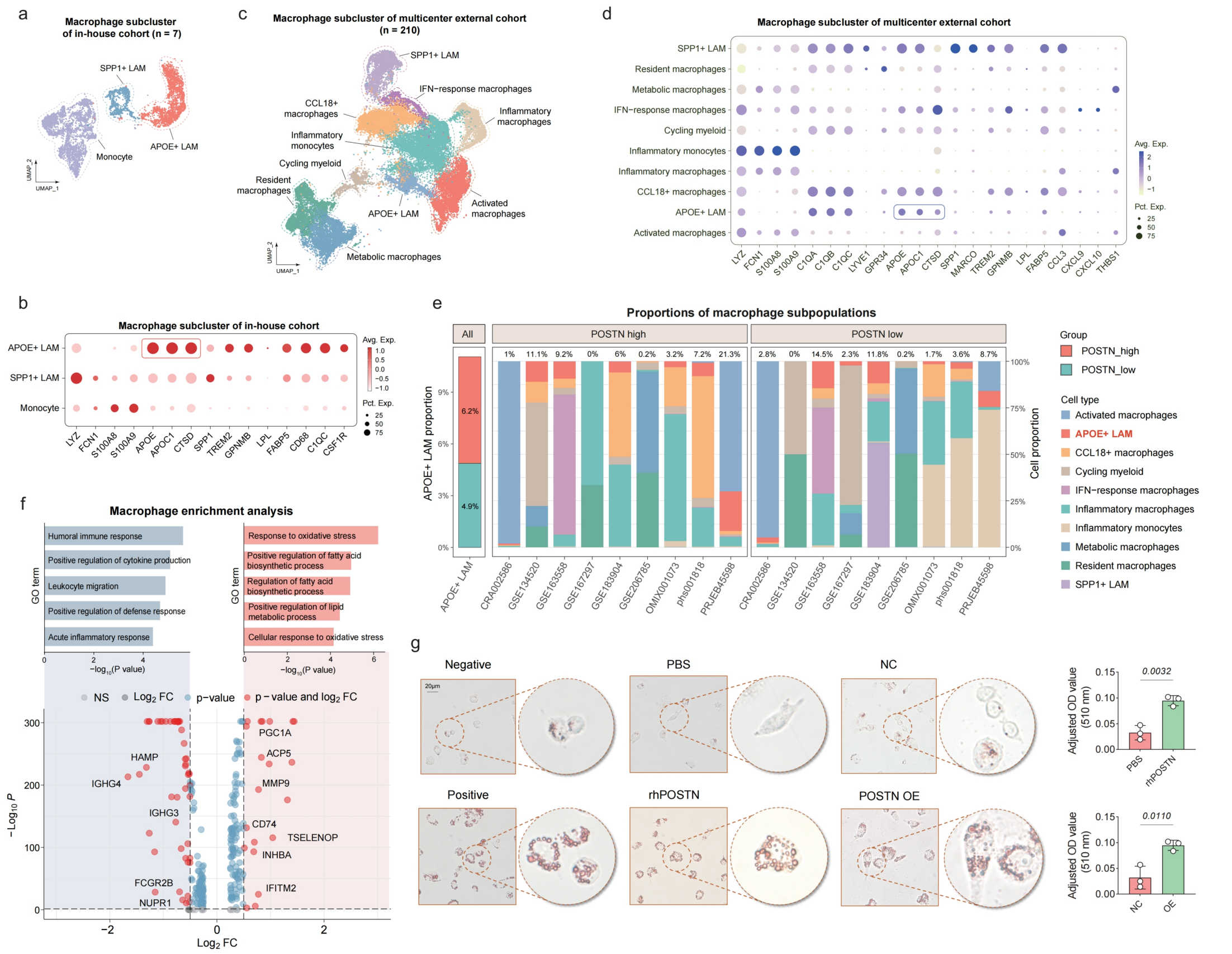

Figure S12. (related to Fig. 4)

Single-cell characterization of POSTN-associated macrophage remodeling and functional validation of POSTN-induced lipid metabolic reprogramming.

(a) UMAP visualization of macrophage subpopulations identified from the in-house single-cell RNA sequencing cohort (n=7). Macrophages were clustered and annotated based on canonical marker expression profiles. **(b)** Dot plot showing the expression patterns of representative marker genes used for macrophage subtype annotation in the in-house cohort (n=7). **(c)** UMAP visualization of macrophage subpopulations identified from the multicenter external single-cell RNA sequencing cohort (n=210). **(d)** Dot plot displaying the expression of representative marker genes across macrophage subpopulations in the multicenter external cohort (n=210). **(e)** Quantification of macrophage subtype composition in the multicenter external cohort. Left panel: proportion of APOE^{+} macrophages in POSTN-high and POSTN-low groups across 210 samples. Right panel: distribution of macrophage subpopulations across individual datasets included in the multicenter cohort. **(f)** Differential gene expression analysis between POSTN-high and POSTN-low groups in macrophages from the in-house single-cell cohort (n=7). The volcano plot

shows differentially expressed genes, and the corresponding enrichment analysis displays the significantly enriched biological pathways associated with POSTN-associated macrophage remodeling. **(g)** Oil Red O staining demonstrating lipid accumulation in macrophages treated with recombinant human POSTN (rhPOSTN) or conditioned medium derived from POSTN-overexpressing fibroblasts.

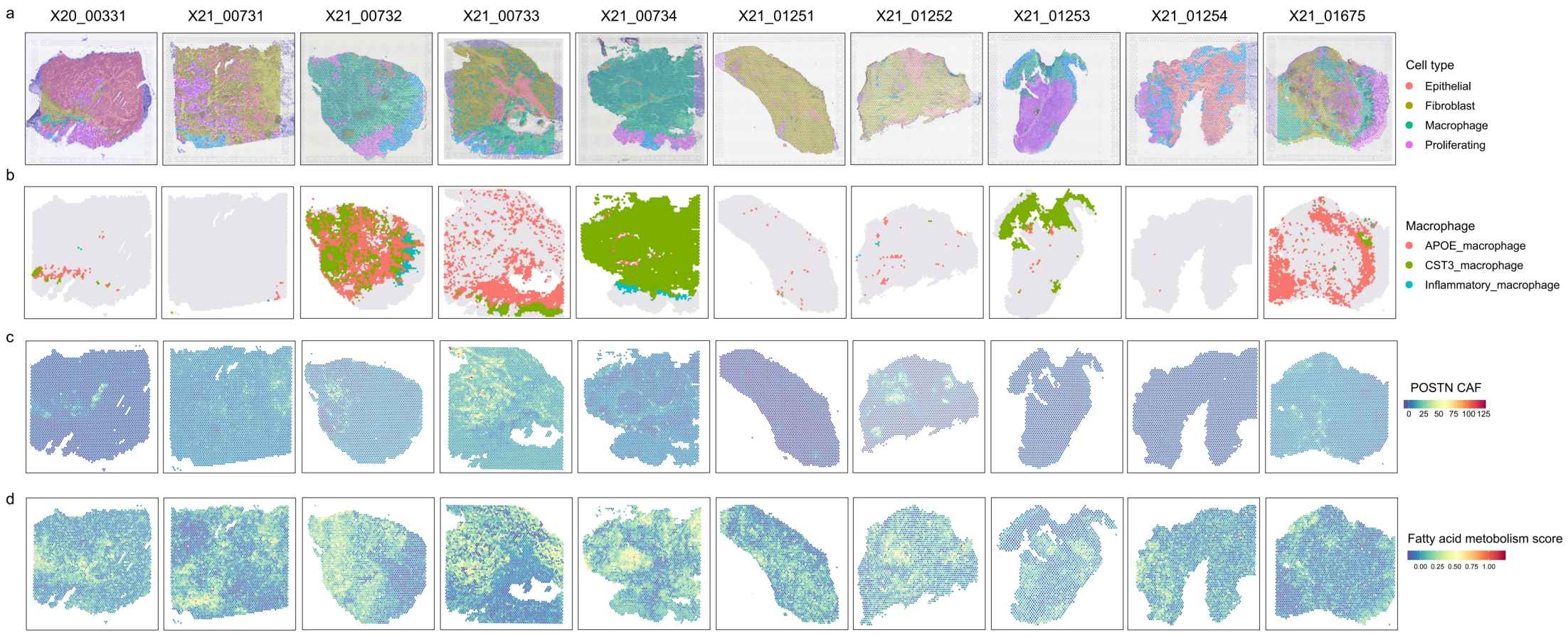

Figure S13. (related to Fig. 4)

Spatial mapping reveals the co-localization of POSTN⁺ CAF niches with macrophage remodeling and lipid metabolic activity. (a) Spatial distribution of major cell populations inferred from spatial transcriptomic data (GSE251950). Cell type annotations were mapped onto individual gastric cancer tissue sections to visualize the spatial organization of the tumor microenvironment. **(b)** Spatial localization and annotation of macrophage subpopulations. **(c)** Spatial distribution of $POSTN^{+}$ CAF enrichment scores across gastric cancer tissue sections. $POSTN^{+}$ CAF enrichment scores were calculated using an established CAF-related gene signature comprising POSTN, COL1A1, COL1A2, DCN, LUM, PDGFRA, and MMP11. **(d)** Spatial distribution of fatty acid metabolism scores across gastric cancer tissue sections. Fatty acid metabolism scores were calculated using a macrophage lipid metabolism-related gene signature comprising APOE, APOC1, CTSD, TREM2, GPNMB, LPL, FABP5, PLIN2, LGALS3, SPP1, MARCO, and MSR1.

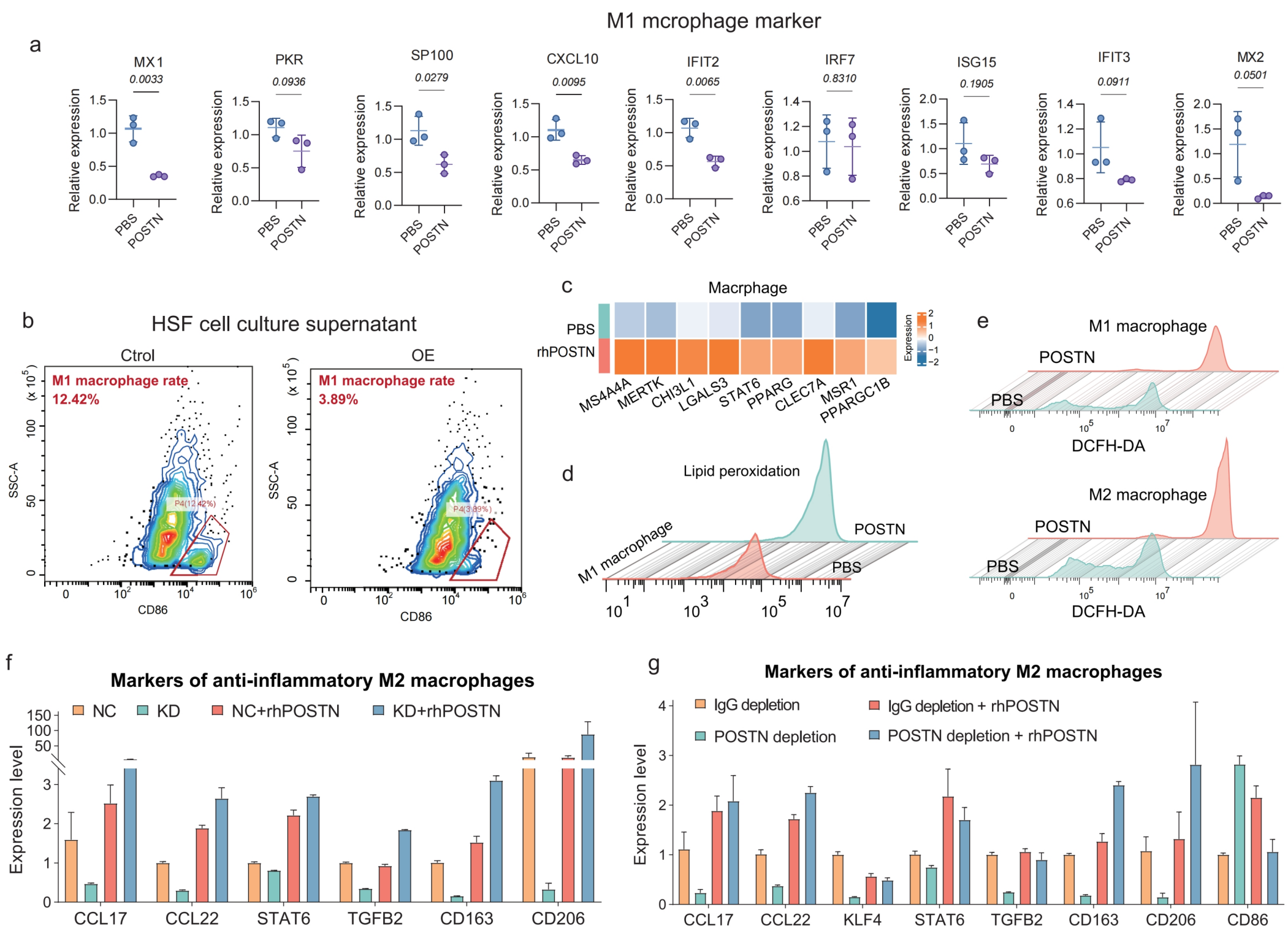

Figure S14. (related to Fig. 4)

Validation of POSTN-induced macrophage metabolic reprogramming. (a) Reduced expression of M1 markers in rhPOSTN-treated THP1 macrophages. **(b)** Flow cytometry plots of M1 macrophage ($CD86^+$) frequency in HSF cell culture supernatants from control (Ctrl, left) and POSTN-overexpressing (OE, right) groups. **(c)** Heatmap of macrophage marker expression in different treatment groups (PBS, rhPOSTN), with color intensity representing expression levels. **(d–e)** Histogram of lipid peroxides (d) and ROS (e) in macrophages after rhPOSTN exposure. **(f)** Expression of macrophage polarization markers following POSTN knockdown and subsequent rhPOSTN rescue. **(g)** Expression of macrophage polarization markers following antibody-mediated depletion of POSTN from fibroblast-CM and subsequent rhPOSTN reconstitution. Unless otherwise indicated, data are presented as mean ± SD from three independent biological replicates (n = 3).

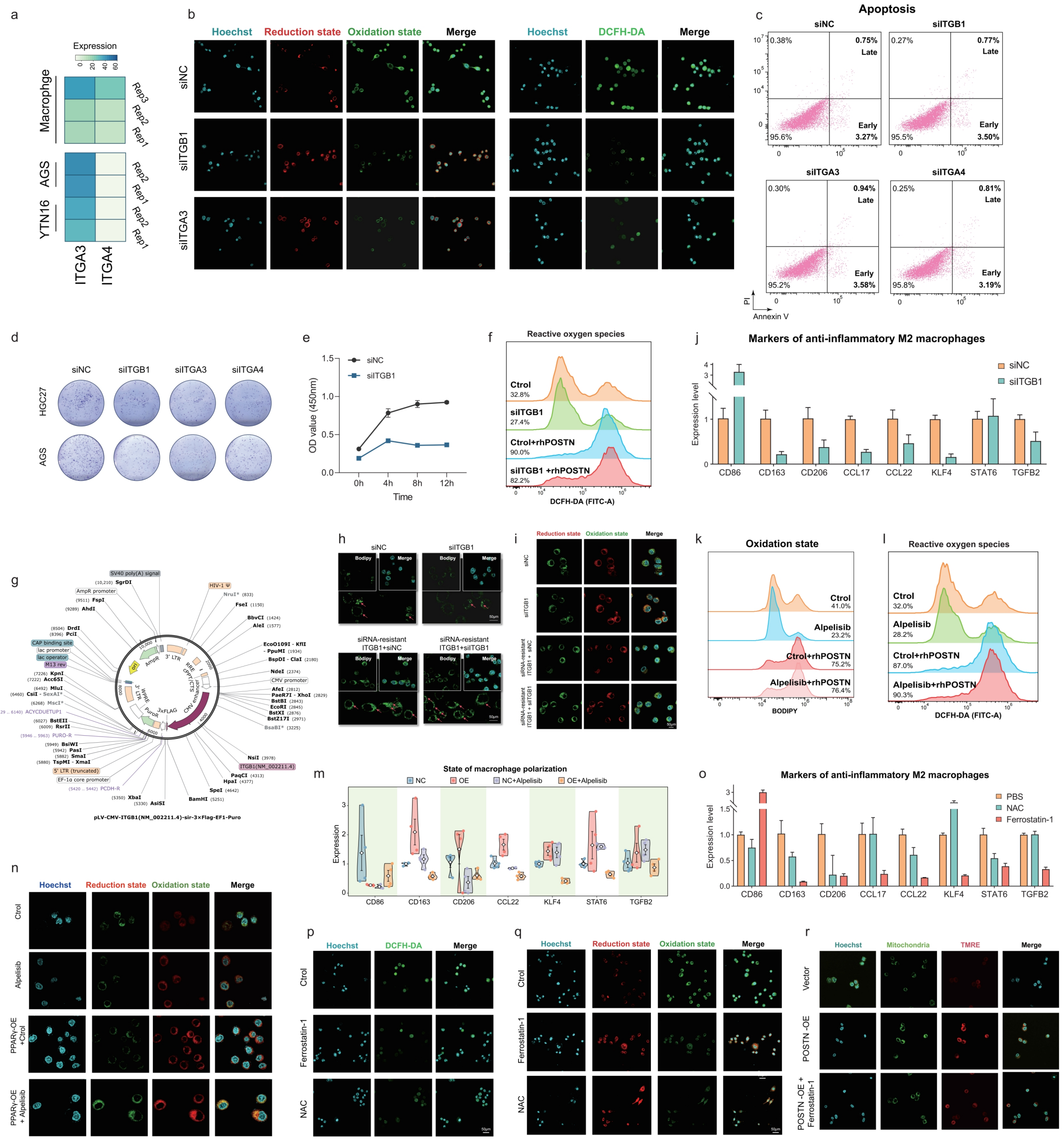

Figure S15. (related to Fig. 5)

Additional validation of the POSTN–ITGB1–PI3K/AKT/mTOR–PPARγ signaling axis and its downstream metabolic and immunological effects. (a) Validation of predicted integrin receptor expression in different cell types. RT-qPCR analysis showing relatively high expression of ITGA3 in AGS and YTN16 gastric cancer cells and ITGA4 expression in macrophages, supporting the ligand–receptor interactions predicted by single-cell communication analysis. **(b)** Representative fluorescence images of lipid oxidation and ROS levels in cells upon ITGB1 or ITGA3 silencing. **(c)** Apoptosis induced by silencing of ITGB1, ITGA3, or ITGA4. **(d)** Colony formation ability of cells after ITGB1, ITGA3, or ITGA4 knockdown. (**e**) CCK-8 proliferation assay showing that ITGB1 knockdown significantly attenuated POSTN-induced tumor cell

proliferation compared with the siNC control group. **(f)** Flow cytometric quantification demonstrating that ITGB1 depletion reduced intracellular ROS accumulation, whereas supplementation with recombinant POSTN restored these phenotypes. **(g)** Schematic map of the siRNA-resistant ITGB1 expression construct, containing silent mutations within the siRNA-targeting sequence while preserving the amino acid coding sequence. **(h)** Representative BODIPY 493/503 fluorescence images showing intracellular lipid droplet accumulation in cells transfected with siNC, siITGB1, siRNA-resistant ITGB1 + siNC, or siRNA-resistant ITGB1 + siITGB1. **(i)** Representative fluorescence images of lipid oxidation status demonstrating that re-expression of siRNA-resistant ITGB1 rescued the reduction in lipid peroxidation caused by ITGB1 silencing. **(j)** Quantification of macrophage polarization markers showing that ITGB1 knockdown significantly reduced M2-associated macrophage polarization. **(k)** Representative lipid oxidation analysis following treatment with the PI3K inhibitor Alpelisib. **(l)** Flow cytometric quantification of intracellular ROS levels in cells treated with Alpelisib. **(m)** Analysis of macrophage polarization markers after Alpelisib treatment ($n = 4$). **(n)** Representative fluorescence images of oxidized lipid accumulation in rescue experiments following Alpelisib-mediated pathway inhibition and re-expression of downstream PPARγ signaling nodes. **(o)** Analysis of macrophage polarization markers following treatment with the antioxidant N-acetyl-L-cysteine (NAC) or the lipid peroxidation inhibitor Ferrostatin-1. **(p)** Representative lipid oxidation profiles showing that NAC and Ferrostatin-1 attenuated POSTN-induced lipid peroxidation. **(q)** Representative fluorescence images of oxidative-state signals after NAC or Ferrostatin-1 treatment. **(r)** TMRE assay showing restoration of mitochondrial membrane potential following Ferrostatin-1 treatment.

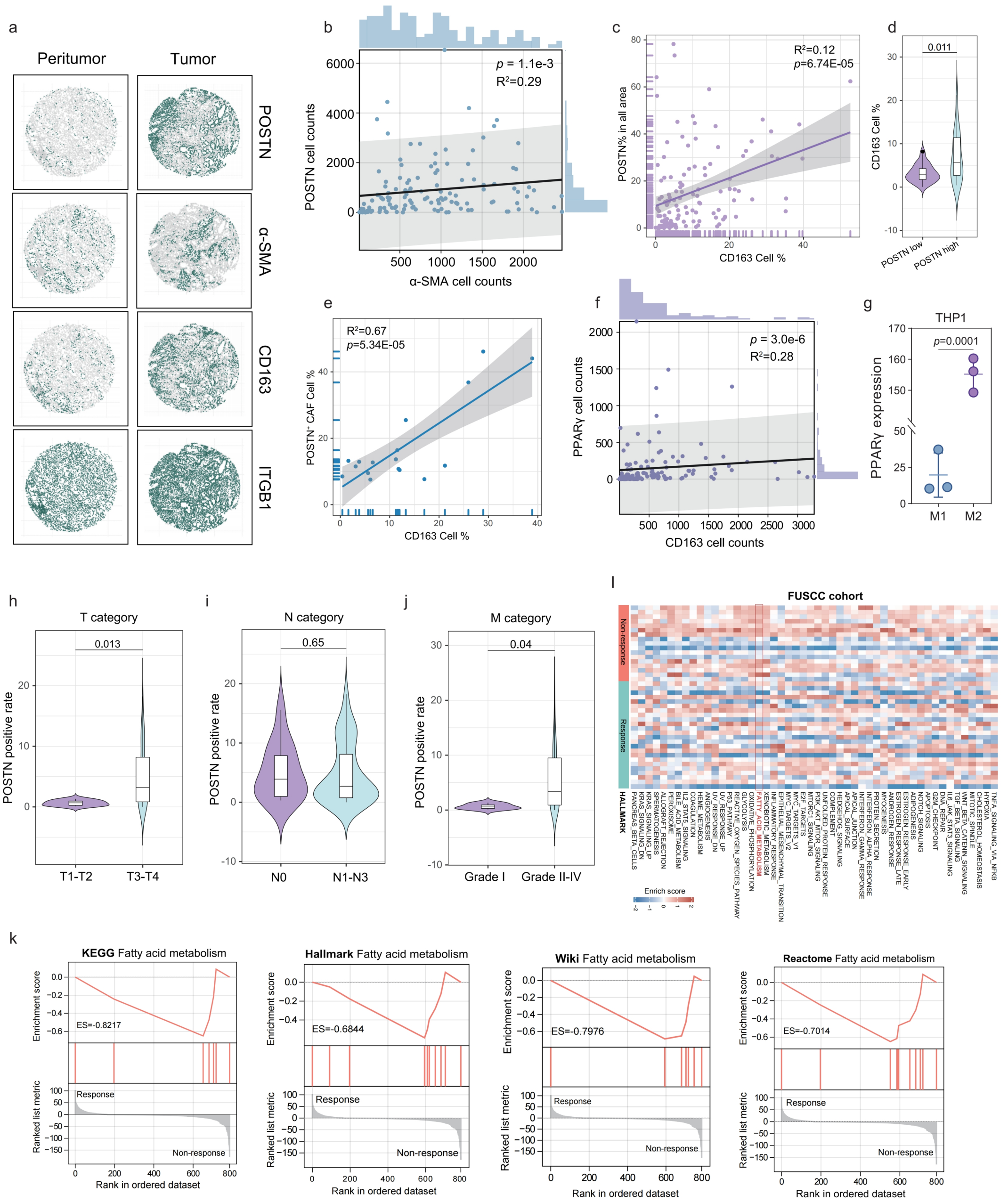

Figure S16. (related to Fig. 6)

Quantitative analysis of POSTN expression and its association with CD163$^+$ macrophages and clinical parameters in GC. (a) Representative images of tumor and peritumor tissue sections, stained for POSTN, α-SMA, CD163, and ITGB1 (color intensity indicates expression level). **(b)** Scatter plot depicting the correlation between the percentage of α-SMA$^+$ cells and POSTN$^+$ area across samples. **(c)** Scatter plot depicting the correlation between the percentage of CD163$^+$ cells and POSTN$^+$ area across samples. Linear regression line (purple) with 95% confidence interval (gray shading). **(d)** Violin plot comparing CD163$^+$ cell percentages between POSTN-low and

POSTN-high groups. **(e)** Scatter plot showing the correlation between CD163$^+$ cell percentage and POSTN$^+$ CAF percentage. Linear regression line (blue) with 95% confidence interval (gray shading). **(f)** Scatter plot depicting the correlation between the percentage of CD163$^+$ cells and PPARγ$^+$ area across samples. **(g)** The box plot illustrates the expression of PPARγ in macrophages with different states of polarization. **(h–j)** Violin plots showing POSTN positive rates across clinical categories: T stage (h), N stage (i), and histological grade (j). **(k)** Gene set enrichment analysis (GSEA) plots for the “Fatty acid metabolism” pathway from the KEGG, Hallmark, WikiPathways and Reactome databases, comparing responder and non-responder groups. **(l)** Heatmap of Hallmark pathway enrichment in the FUSCC GC cohort, stratified by treatment response (non-responder vs. responder). Color scale represents normalized enrichment score (blue, low; red, high). Unless otherwise indicated, data are presented as mean ± SD from three independent biological replicates (n = 3).

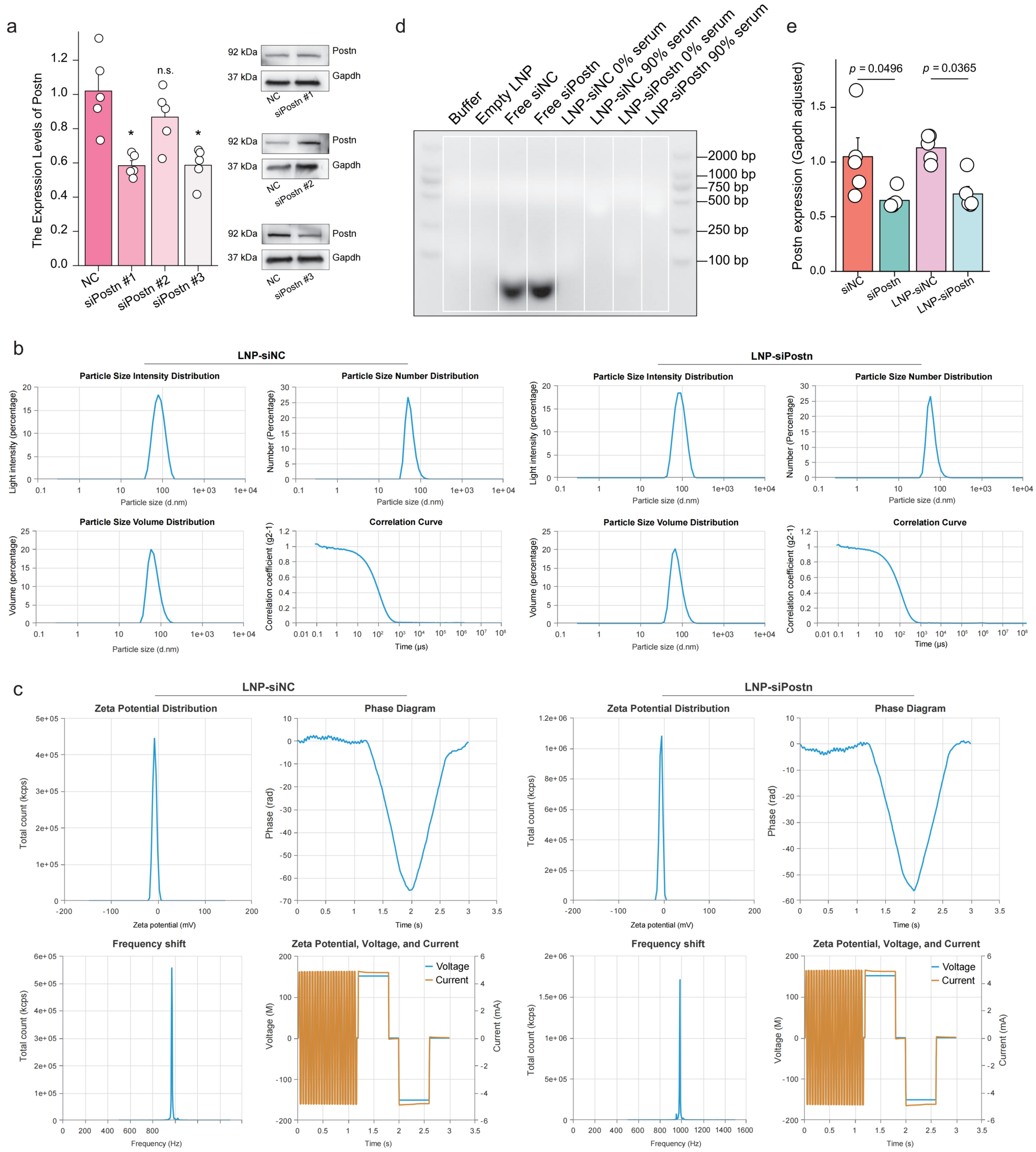

Figure S17. (related to Fig. 7)

Characterization of LNP-siPostn formulation and validation of Postn silencing efficiency. (a) Validation of Postn-targeting siRNAs. Left: qPCR analysis of Postn mRNA expression in GC cells treated with siNC or three Postn-targeting siRNAs (siPostn#1–3, n = 5). Right: Western blot analysis of Postn protein expression, with GAPDH as loading control. **(b)** Dynamic light scattering (DLS) analysis showing the hydrodynamic diameter and particle size distribution of the formulated LNP-siPostn nanoparticles. **(c)** Zeta-potential measurements of LNP-siPostn nanoparticles, demonstrating surface charge characteristics associated with colloidal stability. **(d)** Agarose gel electrophoresis analysis of siRNA stability under different conditions: buffer, empty LNP, free siPostn, LNP-siPostn, and LNP-siPostn incubated with increasing serum concentrations

(0%–90%). **(e)** qPCR quantification of Postn mRNA in GC cells treated with siNC, LNP-siNC, empty LNP, or LNP-siPostn (n = 5).

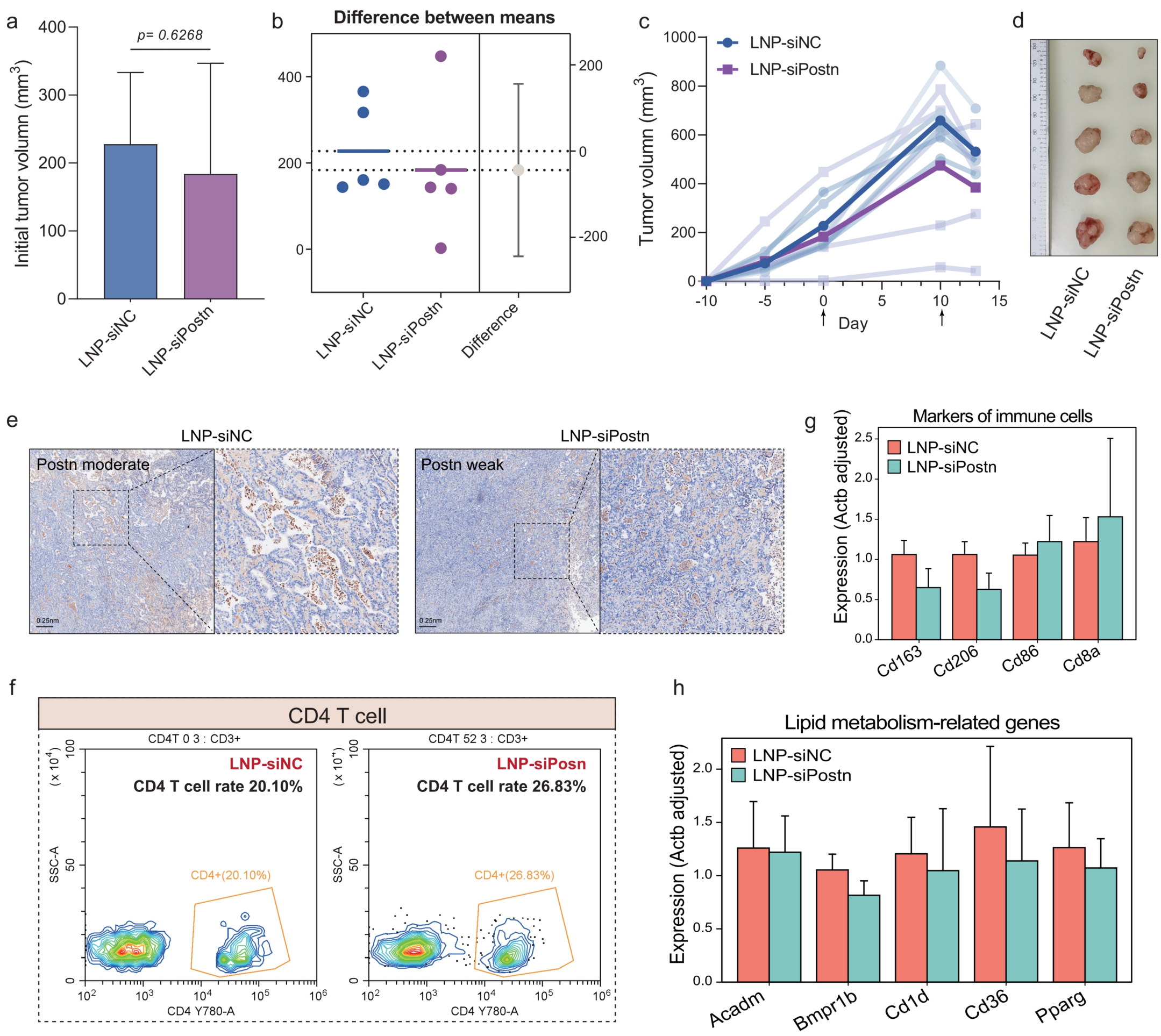

Figure S18. (related to Fig. 7)

Effects of LNP-siPostn treatment on tumor growth and immune remodeling in vivo. (a) Bar graph of initial tumor volume before treatment with LNP-siNC (negative control) or LNP-siPostn (n = 5). **(b)** Dot plot comparing initial tumor volumes between groups; dashed line indicates the mean difference. **(c)** Tumor growth curves of mice treated with LNP-siNC or LNP-siPostn, with arrows marking the time points of intravenous injections. **(d)** Photograph of excised tumors from LNP-siNC and LNP-siPostn treated mice. **(e)** Representative immunohistochemical staining images of Postn in GC tissues from mice treated with LNP-siNC (left) or LNP-siPostn (right). **(f)** Flow cytometry plots showing $CD4^{+}$ T cell frequencies in tumor tissues, with percentages indicated. **(g)** Bar graph of macrophage (Cd163, Cd206, Cd86) and T cell (Cd8a) marker expression in tumor tissues from NC versus siPostn groups. **(h)** Bar graph of lipid metabolism–related gene expression (Acadm, Bmpr1b, Cd1d, Cd36, Pparg) in tumor tissues from NC versus siPostn groups. Unless otherwise indicated, data are presented as mean ± SD from

three independent biological replicates (n = 3).

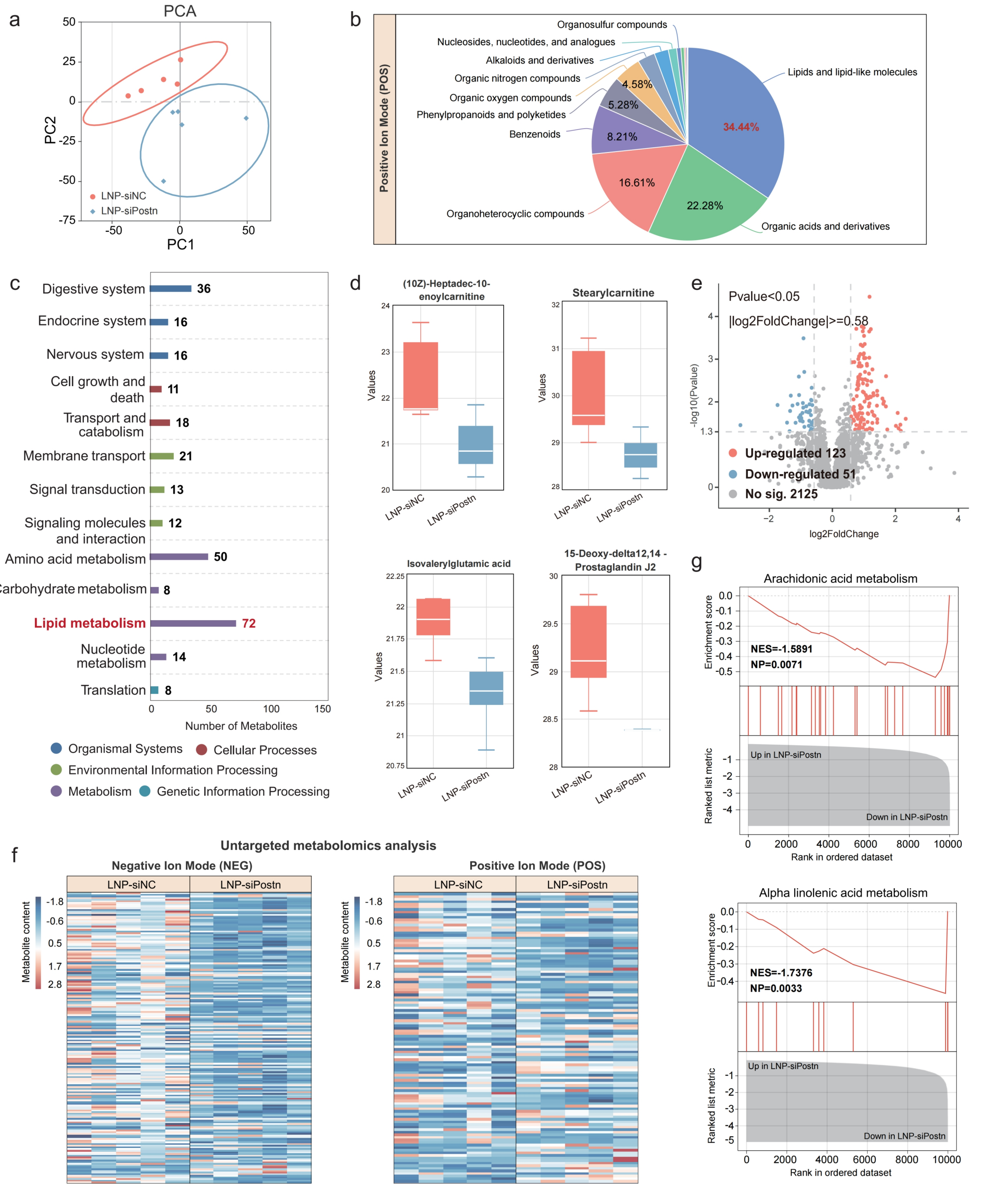

Figure S19. (related to Fig. 7)

Metabolomic profiling of LNP-siPostn treatment in GC. (a) Principal component analysis (PCA) score plot showing distinct metabolic profiles between LNP-siNC (red) and LNP-siPostn (blue) groups (n = 5). **(b)** Pie charts of metabolite distribution detected in positive ion mode (POS), classified by compound type. **(c)** Kyoto Encyclopedia of Genes and Genomes (KEGG) pathway classification bar graph, with metabolite counts in each pathway. **(d)** Box plots of representative lipid metabolites (10Z-Heptadec-10-enoylcarnitine, Stearylcarnitine, Isovalerylglutamic acid, and 15-Deoxy-Δ12,14-Prostaglandin J2), showing significant abundance differences between

LNP-siNC and LNP-siPostn groups. **(e)** Volcano plot of differentially abundant metabolites between groups. Red, upregulated metabolites (n=123); blue, downregulated metabolites (n=51); gray, non-significant metabolites (n=2,125). **(f)** Heatmaps of metabolite intensity in NEG (left) and POS (right) modes. Color scale represents normalized abundance (red, high; blue, low). **(g)** Gene set enrichment analysis (GSEA) plots for arachidonic acid metabolism (ES = -1.5891, NP = 0.0071) and alpha linolenic acid metabolism (ES = -1.7376, NP = 0.0033), showing down-regulation in LNP-siPostn. Unless otherwise indicated, data are presented as mean ± SD from three independent biological replicates (n = 3).

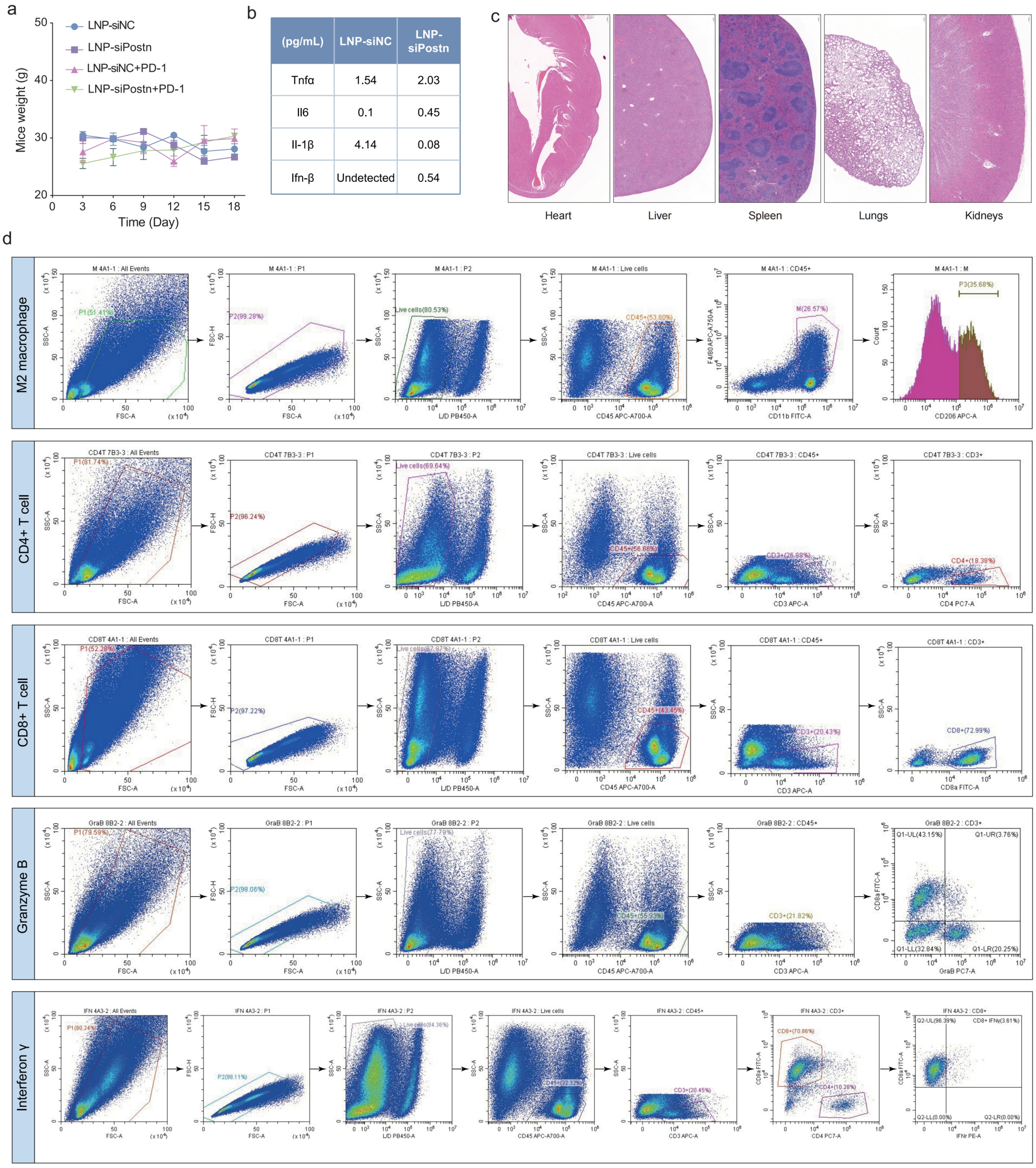

Figure S20. (related to Fig. 8)

Safety evaluation of LNP-siPostn treatment and flow cytometry gating strategy for immune profiling. (a) Body weight changes of mice receiving the indicated treatments throughout the therapeutic study. Body weights were monitored at the indicated time points to assess systemic tolerability (n = 6). **(b)** Serum inflammatory cytokine levels in mice treated with LNP-siPostn. Concentrations of Tnf-α, Il-6, Il-1β, and Ifn-β were measured to evaluate potential systemic immune activation following treatment. **(c)** Representative hematoxylin and eosin (H&E) staining of major organs, including heart, liver, spleen, lung, and kidney, collected from mice after treatment. No overt pathological abnormalities, inflammatory infiltration, or tissue damage were observed. **(d)** Flow cytometry gating strategy used for immune profiling of tumor-infiltrating

immune cells. Representative gating scheme showing sequential selection of singlets and live cells, followed by identification and quantification of major immune cell populations, including M2-like macrophages, $CD4^+$ T cells, $CD8^+$ T cells, and intracellular Granzyme B^+ or IFN-γ^+ effector T-cell populations.

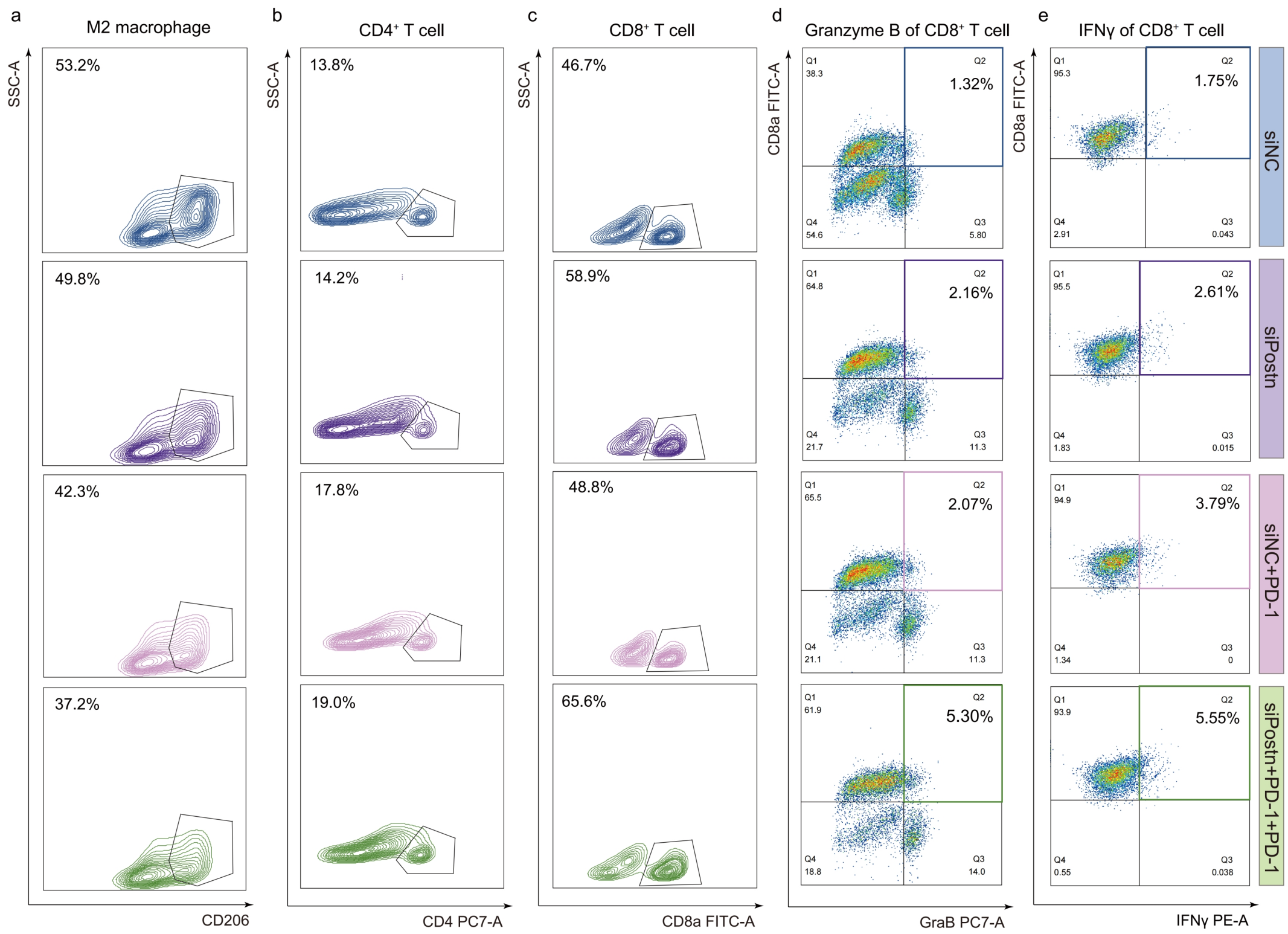

Figure S21. (related to Fig. 8)

Representative flow cytometry plots of tumor-infiltrating immune cell populations after combination therapy. (a) Representative flow cytometry plots showing CD206$^+$ M2-like macrophages in the indicated treatment groups. **(b)** Representative flow cytometry plots showing CD4$^+$ T-cell infiltration in the indicated treatment groups. **(c)** Representative flow cytometry plots showing CD8$^+$ T-cell infiltration in the indicated treatment groups. **(d)** Representative flow cytometry plots showing Granzyme B$^+$ effector T cells in the indicated treatment groups. **(e)** Representative flow cytometry plots showing IFN-γ$^+$ effector T cells in the indicated treatment groups. Treatment groups included LNP-siNC, LNP-siPostn, anti-PD-1 + LNP-siNC, and anti-PD-1 + LNP-siPostn.
